# Software-guided versus nurse-directed blood glucose control in critically ill patients: the LOGIC-2 multicenter randomized controlled clinical trial

**DOI:** 10.1186/s13054-017-1799-6

**Published:** 2017-08-14

**Authors:** Jasperina Dubois, Tom Van Herpe, Roosmarijn T. van Hooijdonk, Ruben Wouters, Domien Coart, Pieter Wouters, Aimé Van Assche, Guy Veraghtert, Bart De Moor, Joost Wauters, Alexander Wilmer, Marcus J. Schultz, Greet Van den Berghe, Dieter Mesotten

**Affiliations:** 1 Clinical Division and Laboratory of Intensive Care Medicine, Academic Department of Cellular and Molecular Medicine, KU Leuven, University Hospitals Leuven, Herestraat 49, B-3000 Leuven, Belgium; 2 Department of Anesthesia & Intensive Care, Jessa Hospital, Salvatorstraat 20, B-3500 Hasselt, Belgium; 3 Department of Electrical Engineering (ESAT), Research Division SCD, iMINDS Future Health Dept, KU Leuven, Kasteelpark Arenberg 10, B-3001 Leuven (Heverlee), Belgium; 4 Department of Intensive Care Medicine, Academic Medical Center, Meibergdreef 9, 1105AZ Amsterdam, The Netherlands; 5 Clinical Department of General Internal Medicine, Medical Intensive Care Unit, University Hospitals Leuven, Herestraat 49, B-3000 Leuven, Belgium

**Keywords:** Blood glucose control, Computer algorithm, Quality of blood glucose control, Glycemic penalty index, Time-in-target, Sepsis, Infection

## Abstract

**Background:**

Blood glucose control in the intensive care unit (ICU) has the potential to save lives. However, maintaining blood glucose concentrations within a chosen target range is difficult in clinical practice and holds risk of potentially harmful hypoglycemia. Clinically validated computer algorithms to guide insulin dosing by nurses have been advocated for better and safer blood glucose control.

**Methods:**

We conducted an international, multicenter, randomized controlled trial involving 1550 adult, medical and surgical critically ill patients, requiring blood glucose control. Patients were randomly assigned to algorithm-guided blood glucose control (LOGIC-C, *n* = 777) or blood glucose control by trained nurses (Nurse-C, *n* = 773) during ICU stay, according to the local target range (80–110 mg/dL or 90–145 mg/dL). The primary outcome measure was the quality of blood glucose control, assessed by the glycemic penalty index (GPI), a measure that penalizes hypoglycemic and hyperglycemic deviations from the chosen target range. Incidence of severe hypoglycemia (<40 mg/dL) was the main safety outcome measure. New infections in ICU, duration of hospital stay, landmark 90-day mortality and quality of life were clinical safety outcome measures.

**Results:**

The median GPI was lower in the LOGIC-C (10.8 IQR 6.2–16.1) than in the Nurse-C group (17.1 IQR 10.6–26.2) (*P* < 0.001). Mean blood glucose was 111 mg/dL (SD 15) in LOCIC-C versus 119 mg/dL (SD 21) in Nurse-C, whereas the median time-in-target range was 67.0% (IQR 52.1–80.1) in LOGIC-C versus 47.1% (IQR 28.1–65.0) in the Nurse-C group (both *P* < 0.001). The fraction of patients with severe hypoglycemia did not differ between LOGIC-C (0.9%) and Nurse-C (1.2%) (*P* = 0.6). The clinical safety outcomes did not differ between groups. The sampling interval was 2.3 h (SD 0.5) in the LOGIC-C group versus 3.0 h (SD 0.8) in the Nurse-C group (*P* < 0.001).

**Conclusions:**

In a randomized controlled trial of a mixed critically ill patient population, the use of the LOGIC-Insulin blood glucose control algorithm, compared with blood glucose control by expert nurses, improved the quality of blood glucose control without increasing hypoglycemia.

**Trial registration:**

ClinicalTrials.gov, NCT02056353. Registered on 4 February 2014.

**Electronic supplementary material:**

The online version of this article (doi:10.1186/s13054-017-1799-6) contains supplementary material, which is available to authorized users.

## Background

Elevated blood glucose levels are very common in critically ill patients, independent of pre-existing diabetes mellitus. This hyperglycemia has been associated with an increased risk of morbidity and mortality [1]. Tight blood glucose control (BGC), targeting blood glucose levels below 110 mg/dL, improved the outcome of critically ill patients only in well-controlled single-center trials and in implementation studies [2–6]. In large multicenter trials, however, this beneficial effect was not reproduced and in the NICE-SUGAR trial mortality even increased in the intensive BGC group [7–9]. While excessive hyperglycemia above 180 mg/dL is no longer accepted, the target range for BGC is still controversial and variable [10]. The American Diabetes Association recommends targeting blood glucose levels below 180 mg/dL, while in Europe most centers use stricter target ranges (below 145 mg/dL), despite a lack of evidence [11, 12].

Excess mortality in patients undergoing BGC has been attributed to the increased incidence of hypoglycemia and unnecessary blood glucose variability [13, 14]. Inaccurate blood glucose measurements and the use of insulin dosing protocols that have not been clinically validated play an important role herein [15]. BGC requires not only frequent measurements of blood glucose, but also difficult calculations of the insulin doses, depending on the protocol. Moreover, BGC has to be done in severely ill patients, who may undergo rapid changes in their insulin sensitivity due to the underlying illness and medication such as parenteral nutrition and steroids. To obtain better (less hyperglycemia) and safer (less hypoglycemia) BGC it has been advocated to use computerized protocols for insulin dosing and timing of blood glucose measurements [16, 17]. However, the current evidence for these computer algorithms consists primarily of implementation studies or small randomized controlled studies [18–22]. Therefore, their use has not yet been widely adopted in general clinical practice although a growing number of ICUs are starting to use validated computer algorithms with a beneficial effect on blood glucose control, such as EndoTool and STAR [23, 24].

The LOGIC-Insulin algorithm, developed and based on the Leuven guideline and practice, had already been clinically validated in a single-center randomized trial, showing improved and safer BGC (below 110 mg/dL), compared to that performed by nurses who were highly experienced in BGC [25]. However, performance of the algorithm in a setting of different blood glucose target ranges, in a wide-ranging patient population, by nurses with less experience in BGC has not yet been evaluated in a pragmatic, multicenter setting. Therefore, we hypothesized that the LOGIC-Insulin algorithm improves the quality of BGC, in comparison with nurse-directed BGC, using two different glucose target ranges (80–110 mg/dL or 90–145 mg/dL) in a heterogeneous population of critically ill adults and in the setting of a nursing staff with variable levels of expertise.

## Methods

### Study design

We conducted a pragmatic, parallel-group, observer-blinded, randomized controlled trial, involving medical and surgical patients admitted to the ICUs of three hospitals: two tertiary referral academic centers (University Hospitals of the KU Leuven, Leuven, Belgium and Academic Medical Center at the University of Amsterdam, Amsterdam, The Netherlands) and one non-academic, teaching hospital (Jessa Hospital, Hasselt, Belgium). All ICUs involved have a closed organization, staffed by full-time intensivists, and represent, respectively, 98, 50, and 36 ICU beds.

The study protocol and informed consent documents were approved by the Belgian Federal Agency for Medicines and Health Products (80 M0563) and the institutional review boards of each participating center. The trial was registered on ClinicalTrials.gov (NCT02056353) on 4 February 2014. No design changes occurred during the trial. Whenever possible, informed consent was asked for from the decision-competent patient before study inclusion. In the case of emergency ICU admissions, deferred proxy consent from the legal representative was obtained. The data and safety monitoring board (DSMB) reviewed the data twice according to the charter and advised continuation according to the initial protocol.

### Participants

**Fig. 1:**
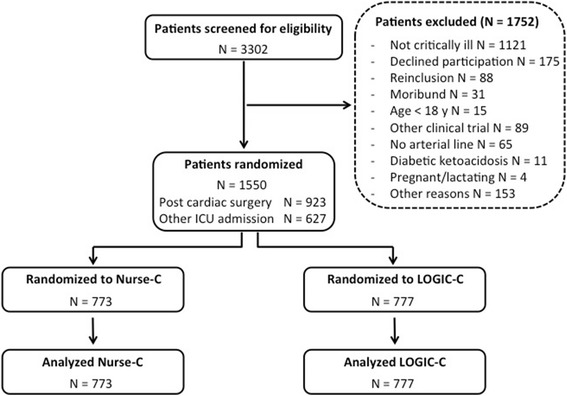
Recruitment of patients into the study. All patients admitted to the ICU in the three participating centers from 24 February 2014 until 17 December 2014, in whom blood glucose control needed to be initiated, were screened for eligibility. This resulted in 1550 patients who were randomized and analyzed (923 patients after cardiac surgery and 627 patients for other reasons as predefined). All patients were included in the primary analysis. *LOGIC*-*C* patients randomized to algorithm-guided blood glucose control, *Nurse*-*C* patients randomized to blood glucose control by trained nurses

Patients were recruited from 24 February 2014 to 17 December 2014. All patients admitted to the ICU with an expected stay of at least 2 days and already receiving or potentially needing insulin for blood glucose control were screened for eligibility (Fig. 1). Exclusion criteria were: not critically ill (monitoring only, not requiring vital organ support), moribund on admission, younger than 18 years of age, enrolled in another intervention trial, no arterial line, pregnant or breast feeding, diabetic ketoacidosis or hyperosmolar state on admission, previously included in the LOGIC-2 trial, or declined participation.

### Randomization

Consecutive patients were randomly allocated into either the nurse-directed (Nurse-C) or the LOGIC-Insulin-guided (LOGIC-C) group by a central computerized system on a 1:1 ratio with permuted blocks of 10 per stratum. Block size was unknown to all collaborators. Randomization was stratified according to type of admission (post-cardiac surgery or other) and study center. Randomization of cardiac surgery patients was stopped after reaching 60% of the total study population across all centers. Outcome assessors, but not clinical staff, were blinded to treatment allocation.

### Intervention

Patients received blood glucose control according to the assigned group: Nurse-C or LOGIC-C. An intravenous infusion of insulin was started on admission in both groups to aim for glycemia in the target range, which in the Leuven and Hasselt ICUs was glycemia 80–110 mg/dL, and in the Amsterdam ICU was glycemia 90–145 mg/dL. Blood glucose targets were identical for both diabetic and non-diabetic patients. In all centers and in both randomization groups insulin treatment was initiated when glycemia exceeded the upper limit of the target range.

BGC was discontinued in both groups when the patients started oral intake of carbohydrates, when the arterial and/or central line was removed, when the patient was discharged to the general ward or to an external ICU, when palliative care was initiated, or when recurrent severe hypoglycemic episodes (glycemia <40 mg/dL) or refractory hyperglycemia (>180 mg/dL and insulin at the aforementioned maximum rate) were observed. Blood glucose concentrations were measured in undiluted arterial blood by an on-site blood gas analyzer, which differed between study sites. Insulin at a concentration of 50 IU in 50 mL of saline was infused through a central venous catheter, preferably via a dedicated lumen, using a syringe pump.

BGC in the Nurse-C group was based on the existing local documented guidelines. All bedside nurses had undergone training in blood glucose management during their schooling as an ICU nurse in the different units. No specific training in BGC was given to nurses performing BGC in the Nurse-C group for the purpose of this study. Hence there was a range of experience in dealing with insulin in the context of BGC. The bedside nurse decided on the timing of blood glucose sampling and the adjustment of the insulin infusion rate according to the best clinical practice.

The LOGIC-insulin algorithm guided BGC in the LOGIC-C group. The LOGIC-Insulin software is an open loop system that advises the nurse on the dose of insulin administration, or on dextrose in the case of hypoglycemia, and on the timing of the subsequent blood glucose measurement. It also entails visual warnings about sampling time, hypoglycemia and potential user entry errors, such as nutrition. All LOGIC-Insulin advices have to be confirmed by the bedside nurse and, if appropriate, can be overruled. The heart of the LOGIC-Insulin algorithm itself is a robust, biphasic, and adaptive patient model. The first phase consists of two main parameters: patient profile on the one hand and admission variables, such as diabetes mellitus and the severity of illness, on the other hand. The second phase comprises five variables: patient profile, blood glucose, insulin dose sequence, the administration of steroids, and nutrition.

Further, the algorithm includes both feedback and predictive mechanisms allowing estimation of the effect of future disturbance factors [25, 26]. The combination of both feedback and predictive mechanisms distinguishes the LOGIC-Insulin algorithm from other computerized protocols such as EndoTool since these are mainly based on feedback mechanisms [23]. The LOGIC-Insulin algorithm can be compared to STAR, a computer algorithm that is also based on feedback and predictive mechanisms and which has already been evaluated in two different ICUs [24].

The LOGIC-Insulin algorithm was designed and configured in the pre-study phase to deal with varying blood glucose target ranges. Since there was a 1:2 to 1:3 nurse-patient ratio in the participating hospitals, nurses may have had to treat patients in the two different groups: Nurse-C and LOGIC-C.

### Outcomes and measures

The primary outcome measure of the LOGIC-2 trial was quality of blood glucose control, assessed by the glycemic penalty index (GPI) per patient, during the intervention period, censored at 14 days. This index gives a penalty to all glucose values falling in the hypoglycemic and hyperglycemic zones with a higher penalty value for larger deviations from normoglycemia [27]. The average of all penalties is summarized as the GPI, ranging from 0 to 100.

The other outcome measures of BGC were in line with the recent consensus recommendations on reporting of glycemia in critically ill patients [28]. The most important safety outcome measure was the incidence of severe hypoglycemia (<40 mg/dL). The incidence per patient and the incidence as a proportion of all blood glucose measurements were reported. The incidence of extended hyperglycemia, defined as three consecutive blood glucose measures >180 mg/dL, was added by the DSMB as a safety outcome measure.

Other secondary endpoints were the incidence of mild hypoglycemia (<70 mg/dL), mean arterial blood glucose concentration, hyperglycemic index (area under the curve in the hyperglycemic zone), time-in-target range, time-to-reach-target range, maximal blood glucose difference (marker of blood glucose variability) and the interval between blood glucose measurements (marker of workload), all reported as per-patient metrics with the exception of the incidence of mild hypoglycemia, which is presented both as the incidence per patient and the incidence per proportion of measurements. In the intervention group protocol compliance (patients in whom the LOGIC-insulin software was not followed for a period of at least 8 h) and overrules (recommendations that were not followed by the bedside nurse) of the software were assessed. A distinction was made between minor (absolute insulin dose difference of > 0.1 IU/h and <1 IU/h) and major (≥1 IU/h) overrules.

Clinical safety endpoints were the incidence of new infections in the ICU (as scored by a blinded infectious disease specialist), ventilator days per patient (censored at 14 days), length of stay in the ICU and in the hospital, mortality in ICU and in hospital, and the landmark 90-day mortality. Quality of life was assessed using the EuroQol 5D-3 L questionnaire at ICU admission, ICU discharge and 90 days post-randomization. Sepsis was diagnosed, using the American College of Chest Physicians-Society of Critical Care Medicine criteria [29].

### Statistical analyses

The study was conceived as a superiority trial for improving the quality of blood glucose control, measured by GPI and the time-in-target range, and it was also powered to detect differences in the incidence of mild hypoglycemia (<70 mg/dL) as a safety outcome variable. The sample size calculations were preregistered at the Belgian Federal Agency for Medicines and Health Products. Better performance in BGC in the Nurse-C group was anticipated, as nurses would feel they were to be watched (Hawthorne effect). On the basis of a 5% confidence level (α error) and a 90% statistical power (β error 10%) the study required 458 patients in each arm to detect a decrease in GPI from mean 21 to 18 (sigma 14) in a two-sided test. An increase in the time-in-target range from 45% to 50% with a sigma 29% would require 707 patients per arm. For a decrease in mild hypoglycemia from 27% to 20% (ARR 7%) the study required 769 patients in each arm. To take into account withdrawals, the study was set up for 1550 patients (775 patients in each group).

A subgroup analysis per study center and blood glucose target was preplanned. The following subgroup analyses for specific patient populations were also planned: cardiac surgery, medical, sepsis on admission, infection on admission, and known diabetes mellitus.

Variables were summarized as frequencies and percentages, mean and standard deviation (SD) or median and interquartile range (IQR), as appropriate. Confidence intervals were computed based on the bootstrap percentile method for the primary and secondary endpoints [30].

All analyses were done on an intention-to-treat basis. Data were compared using the chi-square (χ^2^) (Fisher exact) test, Student *t* test, or nonparametric (Wilcoxon rank sum) test as appropriate. For all endpoints, differences were considered statistically significant whenever the two-sided *P* value was <0.05, without correction for multiple testing. For the statistical analyses, JMP Pro 11 (SAS Institute, Cary, NC, USA) and Matlab (MathWorks, Natick, MA, USA) were used.

## Results

### Study participants

A total of 3302 patients were screened for eligibility, of whom 1550 (47%) gave consent and were randomized to either Nurse-C or LOGIC-C (Fig. 1). None of the patients was lost to follow up. The baseline characteristics of the treatment groups were similar (Table 1). The percentage of admissions after cardiac surgery was 59% and 60% in the Nurse-C and the LOGIC-C group, respectively. On admission, 21.6% of patients had known diabetes mellitus and 28% an infection.

**Table 1:** Baseline characteristics

	Nurse-C	LOGIC-C
Total	773	777
Age, mean (SD), years	66 (15)	66 (14)
Male, *n* (%)	478 (62)	495 (64)
BMI, mean (SD), kg/m^2^	26.4 (5.2)	26.3 (4.9)
Diabetes, *n* (%)	167 (21.6)	168 (21.6)
Chronic dialysis, *n* (%)	17 (2)	11 (1)
APACHE-II score, mean (SD)	20 (9)	21 (9)
Admission type		
Cardiac surgery, *n* (%)	458 (59)	465 (60)
Other surgery, *n* (%)	164 (21)	130 (17)
Medical, *n* (%)	131 (17)	164 (21)
Transplantation, *n* (%)	20 (3)	18 (2)
Adm mechanical ventilation, *n* (%)	661 (86)	657 (85)
Adm insulin infusion, *n* (%)	171 (21)	164 (22)
Adm blood glucose level (mg/dL), median (IQR)	127 (108–153)	128 (106–153)
Adm hypoglycemia (<40 mg/dL), *n* (%)	1 (0.1)	1 (0.1)
Adm blood lactate level (mmol/L), median (IQR)	1.3 (1.0–1.9)	1.3 (1.0–2.0)
Adm infection, *n* (%)	219 (28)	217 (28)
Adm sepsis, *n* (%)	102 (13)	129 (17)
Adm EQ-5D value index (%), median (IQR)	0.72 (0.35–1)	0.71 (0.33–0.90)
Adm EQ-5D VAS score (%), median (IQR)	0.65 (0.50–0.80)	0.65 (0.42–0.80)

Data are mean (SD), median (IQR) or *n* (%) *LOGIC*-*C* patients randomized to algorithm-guided blood glucose control, *Nurse*-*C* patients randomized to blood glucose control by trained nurses, *APACHE*-*II* Acute Physiology and Chronic Health Evaluation-II, *BMI* body mass index, *Adm* admission, *EQ*-*5D* Euroqol-5D, *VAS* visual analog scale

### Blood glucose control

The GPI, the primary outcome measure, was 6.3 points lower in the LOGIC-C group than in the Nurse-C group (*P* < 0.001) (Table 2). Time-in-target range was increased from 47.1% in the Nurse-C group to 67.0% in LOGIC-C group (*P* < 0.001). Mean blood glucose levels and the hyperglycemic index were also lower in the LOGIC-C group (all *P* < 0.001). Moreover, blood glucose variability was decreased in the LOGIC-C group (*P* < 0.001). The proportion of patients experiencing at least one episode of hypoglycemia did not differ between treatment groups (all *P* > 0.07). However, the proportion of blood glucose readings <70 mg/dL and <60 mg/dL was smaller in the LOGIC-C group (both *P* = 0.02). None of the subjects in either randomized group experienced recurrent severe hypoglycemic episodes or refractory hyperglycemia that warranted withdrawal from the study. Workload was higher in the LOGIC-C group, as reflected in a 23% shorter sampling interval (*P* < 0.001).

**Table 2:** Blood glucose control in the two randomized groups

	Nurse-C		LOGIC-C		*P* value
		95% CI		95% CI	
Patients	773		777		
Study period, median (IQR), days	2 (2–4)		2 (2–4))		0.7
Efficacy					
Glycemic penalty index (GPI), median (IQR)	17.1 (10.6–26.2)	16.3–18.1	10.8 (6.2–16.1)	10.0–11.5	<0.001
Blood glucose, mean (SD), mg/dL	119 (21)	118–121	111 (15)	110–112	<0.001
Minimum blood glucose, mg/dL	21		26		
Maximum blood glucose, mg/dL	428		511		
Hyperglycemic index, median (IQR), mg/dL	8.3 (3.4–16.3)	7.5–8.8	3.5 (1.4–7.0)	3.1–4.0	<0.001
Time-in-target range, median (IQR), percentage	47.1 (28.1–65.0)	45.7–49.3	67.0 (52.1–80.1)	65.4–68.6	<0.001
Time to reach target range, median (IQR), h	3.6 (0–9.3)	3.2–4.3	2.2 (0–5.1)	1.8–2.5	<0.001
Mean of maximum delta glycemia per day, median (IQR), mg/dL	36 (27–52)	35–38	34 (24–46)	32–36	<0.001
Safety					
Hypoglycemia, proportion of patients					
<70 mg/dL, *n* (%)	173 (22.4)	151–196	149 (19.2)	128 –171	0.1
<60 mg/dL, *n* (%)	78 (10.1)	62–95	58 (7.5)	44–73	0.07
<40 mg/dL, *n* (%)	9 (1.2)	4–15	7 (0.9)	2–13	0.6
Hypoglycemia, proportion of samples					
<70 mg/dL, *n* (%)	346 (1.8)	311–382	342 (1.5)	306–379	0.02
<60 mg/dL, *n* (%)	123 (0.7)	102–145	105 (0.5)	85–125	0.02
<40 mg/dL, *n* (%)	9 (0.05)	4–15	9 (0.04)	4–15	0.9
Workload					
Sampling interval, mean (SD), h	3.0 (0.8)	2.9–3.0	2.3 (0.5)	2.3–2.3	<0.001

Data are mean (SD), median (IQR) or *n* (%). *LOGIC*-*C* patients randomized to algorithm-guided blood glucose control, *Nurse*-*C* patients randomized to blood glucose control by trained nurses

### Predefined subgroups

**Fig. 2:**
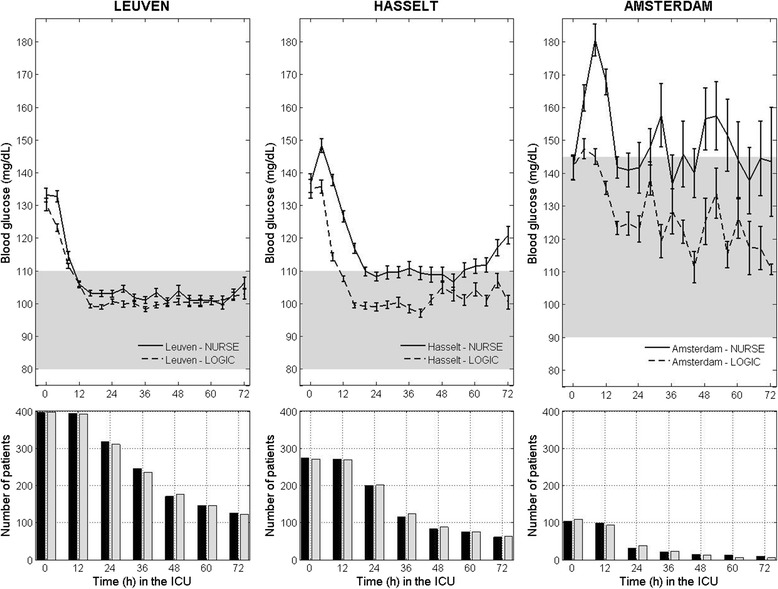
Blood glucose control and numbers of patients in the two randomized groups during the first 3 days in the ICU, per study center. *Upper panels* overall mean blood glucose levels (mg/dL) for both the algorithm-guided (*dashed line*) and the nurse-directed (*solid line*) blood glucose control group per center during the first 72 h in the ICU, which is the median ICU stay. The average blood glucose is computed over all glucose samples (per center) belonging to the previous 4-h time slot. The glycemic target range was 80–110 mg/dL for Leuven and Hasselt, unlike Amsterdam where the glycemic target range was 90–145 mg/dL. *Lower panels* the number of patients for the Nurse-C group (*Nurse*) (*black bars*) and LOGIC-C group (*LOGIC*) (*gray bars*)

The effect of the LOGIC-Insulin algorithm on BGC was more pronounced in Hasselt and Amsterdam (Fig. 2). While the GPI was 3.6 points lower in the Leuven LOGIC-C group, the difference was 10.1 in Hasselt and 15.1 in Amsterdam (Additional file 1: Tables S1-S3). Furthermore, LOGIC-Insulin-guided BGC resulted in mean blood glucose levels in the target range for Hasselt (110 mg/dL) and Amsterdam (134 mg/dL), whereas those in the Nurse-C group were above the target range, at 123 mg/dL and 150 mg/dL, respectively. This was in contrast to Leuven, where the mean blood glucose level was in the target range in both the Logic-C (106 mg/dL) and the Nurse-C (109 mg/dL) groups. While the incidence of mild hypoglycemia decreased in the Leuven LOGIC-C group, it increased in the Hasselt LOGIC-C group, compared with their respective Nurse-C groups. In all three centers, the sampling interval was shorter in the LOGIC-C, compared with the Nurse-C group.

The quality of BGC was improved by the algorithm in the predefined subgroups of cardiac surgery (Additional file 1: Table S4), medical admission (Additional file 1: Table S6), sepsis on admission (Additional file 1: Table S8), infection on admission (Additional file 1: Table S10) and known diabetes mellitus (Additional file 1: Table S12).

### Factors interfering with blood glucose control and compliance with the LOGIC-Insulin software during the study period

Patients in the LOGIC-C group received a larger daily insulin dose (median 32 IU/day IQR 18–48 versus median 27 IU/day IQR 14–43 in the Nurse-C group) (*P* < 0.001). The median daily amount of carbohydrates was 42 g (IQR 31–68) in the LOGIC-C and 42 g (IQR 30–70) in the Nurse-C group (*P* = 0.88). The number of LOGIC-C patients who received steroids (24.1%) was comparable to the Nurse-C group (21.7%) (*P* = 0.28). Also the proportion of patients on mixed-bag parenteral nutrition did not differ between the LOGIC-C (7.3%) and the Nurse-C (10.0%) groups (*P* = 0.07). The number of days patients received inotropics/vasopressors (2 days, IQR 1–3 versus 2 days, IQR 1–3) and antibiotics (1 day, IQR 1–3 versus 2 days, IQR 1–3) did not differ between treatment groups (both *P* > 0.39).

In 80/777 patients (10.3%) in the LOGIC-C group, the software had not been used for more than 8 h. In total 240 minor and 147 major overrules occurred in the LOGIC-C group, representing 1.15% and 0.70% of blood glucose measurements, respectively. Of the major overrules, 14 (9.5%) were justified, and of these only 2 overrules were made to avoid hypoglycemia, representing 2/777 LOGIC-C patients (0.3%). In Amsterdam the proportion of overrules (minor 19 (1.71%), major 20 (1.80%) for insulin dosing advice given in 1114 instances) was higher than in Leuven (minor 155 (1.30%), major 89 (0.75%) for insulin dosing advice given in 11926 instances) and Hasselt (minor 66 (0.84%), major 38 (0.48%) for insulin dosing advice given in 7892 instances) (*P* < 0.001).

### Clinical safety outcomes

The clinical outcomes did not differ between the treatment groups (Table 3 and Additional file 1: Table S16). However, in patients with sepsis on admission the incidence of new infections was lower in the LOGIC-C (20.16%) than in the Nurse-C group (33.33%) (*P* = 0.034). In patients with an infection on admission, the incidence of new infections was 23.04% in the LOGIC-C, compared with 31.96% in the Nurse-C group (*P* = 0.042). For all other predefined subgroups (cardiac surgery, medical admission, and diabetes mellitus) the incidence of new infections was comparable between treatment groups.

**Table 3:** Clinical safety outcome measures

	Nurse-C	LOGIC-C	*P* value
Patients	773	777	
Length of ICU stay, median (IQR), days	3 (2–6)	3 (2–6)	0.39
Length of hospital stay, median (IQR), days	12 (8–23)	12 (8–23)	0.18
Mortality in the ICU, *n* (%)	41 (5.30)	47 (6.05)	0.61
Mortality in the hospital, *n* (%)	84 (10.81)	72 (9.31)	0.35
Mortality at 90 days, *n* (%)	89 (11.71)	91 (11.51)	0.93
Incidence of new infections in the ICU, *n* (%)	117 (15.14)	104 (13.38)	0.35
Ventilator days, median (IQR), days	1 (1–2)	1 (1–3)	0.85
EQ-5D index value at ICU discharge, median (IQR)	0.28 (0.13–0.58)	0.29 (0.13–0.58)	0.59
EQ-5D index value at 90 days, median (IQR)	0.73 (0.56–1)	0.73 (0.56–1)	0.58
EQ-5D VAS score at ICU discharge, median (IQR)	0.60 (0.50–0.70)	0.60 (0.50–0.70)	0.42
EQ-5D VAS score at 90 days, median (IQR)	0.70 (0.60–0.80)	0.70 (0.60–0.80)	0.97

Data are mean (SD), median (IQR) or *n* (%). *LOGIC*-*C* patients randomized to algorithm-guided blood glucose control, *Nurse*-*C* patients randomized to blood glucose control by trained nurses, *EQ*-*5D* Euroqol-5D, *VAS* visual analog scale

## Discussion

Using the LOGIC-Insulin algorithm improved the quality of BGC reflected by a reduction in the GPI, an increase in time-in-target range and a reduction in blood glucose variability, without increasing the incidence of hypoglycemia.

The beneficial effects of software-guided BGC were independent of the chosen target blood glucose range and of the center’s expertise in BGC. Morbidity and mortality did not differ between patients in whom BGC was done either by expert nurses or aided by the LOGIC-Insulin algorithm in the context of similar blood glucose target ranges.

These data confirm the findings from our single center trial, in which LOGIC-Insulin improved tight BGC and lowered the incidence of mild hypoglycemia [25]. However, the treatment effect in the pragmatic multicenter LOGIC-2 trial was twofold that in the LOGIC-1 trial and that for which it was statistically powered. This was explained by more pronounced benefits of algorithm-guided BGC in centers with looser BGC. The performance of the Leuven nurses in BGC, incorporated in the LOGIC-Insulin algorithm, may also have blunted the effect in Leuven. Similar blood glucose profiles in the software groups in Leuven and Hasselt, using the same target range, demonstrate that the LOGIC-Insulin software can be generalized outside the context of centers with extensive BGC experience. The fact that more than 98% of all insulin dosing advice provided by the LOGIC-Insulin algorithm were followed by the nurses in all centers in the algorithm-guided BGC group indicates high protocol compliance and indicates that the results are protocol-induced and not a representation of the nurses’ BGC performance in the intervention group. This further underpins the generalizability of the study findings and the potential for implementation of the LOGIC-Insulin software, independent of the chosen blood glucose target range. This is important as the interaction between the BGC algorithm and patient glucose dynamics may be influenced by the performance of the bedsides nurses, who are the ultimate “controllers” in open-loop BGC software.

The larger treatment effect in the broader target range of 90–145 mg/dL (delta GPI 15.1) than in the narrow target range of 80–110 mg/dL (delta GPI 5.7) was therefore not anticipated. One might have expected that BGC is easier for nurses when the target is broader. This indicates that nurses, in the context of a broader target range, tend to be more lenient towards the upper target limit. The higher incidence of extended hyperglycemia above 180 mg/dL reflects this, in spite of the consensus in all guidelines on BGC in the ICU that excessive hyperglycemia should be avoided. Nevertheless, the higher incidence of overrules in Amsterdam, possible practical differences in the insulin administration, and greater statistical uncertainty due to the smaller patient numbers may have contributed as well. Most likely, BGC will be even looser outside study settings. In contrast, use of the narrower target range results in greater incidence of mild hypoglycemia. Algorithm-guided BGC did not increase the incidence of potentially harmful, severe hypoglycemia, which should always be strictly avoided. A recent randomized controlled trial used computerized insulin-dosing algorithms in the intensive BGC group, but did not show a clinical difference with more conservative, nurse-directed BGC [31].

Algorithm-guided BGC increased the workload, similar to other algorithms [32]. Ten instead of eight blood glucose measurements per day were needed to improve BGC in one patient by software use. The workload of blood glucose measurements may be offset by lowering of the cognitive burden for the nurses in deciding on the right insulin dose [33]. Hence, this slight increase in workload may be inevitable in obtaining safe and effective blood glucose control, especially in the initial phase, aiming for a short time to reach the chosen target range combined with a high time-in-target range, low glucose variability, and avoidance of hypoglycemia, and this in a setting with rapid changes in patients insulin sensitivity and in the presence of external confounding factors such as use of steroids and parenteral nutrition. There is now a consensus that a minimal measurement frequency is needed to provide safe glycemic control without adverse effects on the outcome [28]. Whether the increased workload resulted in more attention paid to the patient (collateral benefit) or less attention to the patient (added risk) cannot be delineated from this study.

The improvement in BGC provided by the software was comparable in all patient populations, such as cardiac surgery, pre-existing diabetes, sepsis, and medical admissions. However, the clinical effects of improved BGC should be the ultimate determinants to evaluate medical interventions. Although the LOGIC-2 trial was not set up to test differences in clinical outcomes, it was monitored. As expected, no clinical differences were seen in the overall patient population. Patients with diabetes mellitus did not benefit more from algorithm-guided BGC, as was seen in a recent trial of BGC in patients after coronary artery bypass graft surgery [34]. However, the level of ﻿pre-admission control of the diabetes mellitus may be the most important factor in determining whether patients benefit from tight BGC.

In patients with infectious problems on admission, improved BGC resulted in lowering of the incidence of new infections. This may be explained by the fact that patients with infectious problems on admission are more severely ill, have greater risk of more severe hyperglycemia, which is more difficult to control, and have greater risk of new infections.

The LOGIC-2 trial has its limitations though. Blood glucose dynamics in the ICU are determined by patient characteristics, by the BGC protocol, and by the experience of the bedside nurses in executing the BGC. Therefore, the differences in the quality of BGC in the Nurse-C group between the centers are most likely multifactorial. The variability in the “baseline” quality of BGC may have affected the performance using the LOGIC-Insulin software. It can also not be excluded that nursing practice in performing BGC changed over time during the study, under the influence of the algorithm. However, participating in a study on BGC had most likely improved the performance of BGC by the nurses over time due to a training effect. The LOGIC-Insulin software was now only tested when using glucose readings from arterial blood measured in accurate blood gas analyzers. To gain further applicability the software should be able to cope with blood glucose measurements from point-of-care, handheld blood glucose meters. Other computerized protocols however, have already used these point-of-care glucometers showing a safe and efficient level of blood glucose control [23, 24]. Efficiency or cost-effectiveness of the LOGIC-Insulin software is still unproven, despite that the present pragmatic multicenter RCT showed that the software works in real-life circumstances.

## Conclusions

In a randomized controlled trial of a mixed critically ill patient population, the use of the LOGIC-Insulin blood glucose control algorithm, compared with blood glucose control by expert nurses, improved the quality of blood glucose control without increasing hypoglycemia. This is demonstrated by an important improvement in all domains of glycemic control: a decrease in GPI, an increase in the time-in-target range, and a decrease in glucose variability. Moreover, the LOGIC-Insulin algorithm was shown to be efficient and safe when used in three different ICUs with local variations in clinical practice, with different blood glucose target ranges, and with different levels of experience in blood glucose control. The slight increase in workload generated by the LOGIC-insulin algorithm should be considered an inevitable trade-off when providing safe and efficient blood glucose control.

## Additional files

Additional file 1: Tables showing predefined subgroup analyses. (DOCX 50 kb)

Additional file 2: Study protocol: Competent Authority submission. (PDF 8786 kb)

## Abbreviations

BGC: Blood glucose control
DSMB: Data and safety monitoring board
GPI: Glycemic penalty index
ICU: Intensive care unit
IQR: Interquartile range
LOGIC-C group: Patients randomized to algorithm-guided blood glucose control
Nurse-C group: Patients randomized to blood glucose control by trained nurses
RCT: Randomized controlled trial
