# Supplementary material for: Software-guided versus nurse-directed blood glucose control in critically ill patients: the LOGIC-2 multicenter randomized controlled clinical trial
Source: Crit Care. 2017 Aug 14;21:212. doi: 10.1186/s13054-017-1799-6 (PMC5557320; doi:10.1186/s13054-017-1799-6)
Supplement: Supplementary file 1 — Tables showing predefined subgroup analyses. (DOCX 50 kb) [file 13054_2017_1799_MOESM1_ESM.docx]

**Table S1: Blood glucose management in the two randomization groups for Leuven**

| **Leuven** |  |  |  |
| --- | --- | --- | --- |
|  | **Nurse-C** | **LOGIC-C** | **P value** |
| Patients | 395 | 396 |  |
| Study period, median (IQR), days | 3 (2-6) | 3 (2-5) | 0.3 |
| **Efficacy** |  |  |  |
| Glycemic penalty index (GPI), median (IQR) | 13.3 (9.0-19.7) | 9.7 (6.2-14.4) | <0.001 |
| Blood glucose, mean (SD), mg/dL | 109 (11) | 106 (9) | <0.001 |
| Minimum blood glucose, mg/dL | 21 | 30 |  |
| Maximum blood glucose, mg/dL | 428 | 323 |  |
| Hyperglycemic index, median (IQR), mg/dL | 5 (3-9) | 3 (1-6) | <0.001 |
| Time in target range, median (IQR), percentage | 56.6 (42.3-71.2) | 69.5 (57.8-80.4) | <0.001 |
| Time to reach target range, median (IQR), hours | 3.3 (0.3-7.3) | 2.0 (0-4.5) | <0.001 |
| Mean of Maximum Delta Glycemia per day, median (IQR), mg/dL | 34 (24-48) | 32 (22-42) | 0.005 |
| **Safety** |  |  |  |
| Hypoglycemia per patient |  |  |  |
| < 70 mg/dL, No (%) | 128 (32.4) | 82 (20.7) | <0.001 |
| < 60 mg/dL, No (%) | 55 (13.9) | 31 (7.8) | 0.006 |
| < 40 mg/dL, No (%) | 8 (2.0) | 2 (0.5) | 0.06 |
| Hypoglycemia per samples |  |  |  |
| < 70 mg/dL, No (%) | 260 (2.4) | 169 (1.3) | <0.001 |
| < 60 mg/dL, No (%) | 93 (0.9) | 49 (0.4) | <0.001 |
| < 40 mg/dL, No (%) | 8 (0.07) | 3 (0.02) | 0.1 |
| **Workload** |  |  |  |
| Sampling interval, mean (SD), hours | 2.9 (0.7) | 2.3 (0.4) | <0.001 |

**Table S2 Blood glucose management in the two randomization groups for Hasselt**

| **Hasselt** |  |  |  |
| --- | --- | --- | --- |
|  | **Nurse-C** | **LOGIC-C** | **P value** |
| Patients | 273 | 271 |  |
| Study period, median (IQR), days | 2 (2-4) | 2 (2-4) | 0.4 |
| **Efficacy** |  |  |  |
| Glycemic penalty index (GPI), median (IQR) | 23.5 (15.9-30.2) | 13.4 (8.8-18.3) | <0.001 |
| Blood glucose, mean (SD), mg/dL | 123 (18) | 110 (12) | <0.001 |
| Minimum blood glucose, mg/dL | 33 | 26 |  |
| Maximum blood glucose, mg/dL | 386 | 511 |  |
| Hyperglycemic index, median (IQR), mg/dL | 15 (8-21) | 5 (2-8) | <0.001 |
| Time in target range, median (IQR), percentage | 34.2 (19.6-52.2) | 59.7 (46.2-73.3) | <0.001 |
| Time to reach target range, median (IQR), hours | 7.0 (0-14.2) | 3.1 (0-6.1) | <0.001 |
| Mean of Maximum Delta Glycemia per day, median (IQR), mg/dL | 39 (30-57) | 36 (26-50) | 0.02 |
| **Safety** |  |  |  |
| Hypoglycemia per patient |  |  |  |
| < 70 mg/dL, No (%) | 41 (15.0 ) | 64 (23.6 ) | 0.01 |
| < 60 mg/dL, No (%) | 21 (7.7 ) | 25 (9.2 ) | 0.5 |
| < 40 mg/dL, No (%) | 1 (0.4 ) | 5 (1.8 ) | 0.1 |
| Hypoglycemia per samples |  |  |  |
| < 70 mg/dL, No (%) | 82 (1.2 ) | 170 (2.1 ) | <0.001 |
| < 60 mg/dL, No (%) | 28 (0.4) | 54 (0.7) | 0.06 |
| < 40 mg/dL, No (%) | 1 (0.01) | 6 (0.07) | 0.2 |
| **Workload** |  |  |  |
| Sampling interval, mean (SD), hours | 2.7 (0.6) | 2.2 (0.4) | <0.001 |

**Table S3 Blood glucose management in the two randomization groups for Amsterdam**

| **Amsterdam** |  |  |  |
| --- | --- | --- | --- |
|  | **Nurse-C** | **LOGIC-C** | **P value** |
| Patients | 105 | 110 |  |
| Study period, median (IQR), days | 1 (1-2) | 2 (1-2) | 0.5 |
| **Efficacy** |  |  |  |
| Glycemic penalty index (GPI), median (IQR) | 21.7 (10.3-34.3) | 6.6 (0.7-15.9) | <0.001 |
| Blood glucose, mean (SD), mg/dL | 150 (24) | 134 (19) | <0.001 |
| Minimum blood glucose, mg/dL | 50 | 41 |  |
| Maximum blood glucose, mg/dL | 362 | 394 |  |
| Hyperglycemic index, median (IQR), mg/dL | 12 (3-24) | 2 (0-8) | <0.001 |
| Time in target range, median (IQR), percentage | 40.2 (15.5-70.4) | 77.3 (54.9-96.0) | <0.001 |
| Time to reach target range, median (IQR), hours | 0 (0-10.8) | 0 (0-4.1) | 0.2 |
| Mean of Maximum Delta Glycemia per day, median (IQR), mg/dL | 49 (25-68) | 38 (29-53) | 0.1 |
| **Safety** |  |  |  |
| Hypoglycemia per patient |  |  |  |
| < 70 mg/dL, No (%) | 4 (3.8) | 3 (2.7) | 0.7 |
| < 60 mg/dL, No (%) | 2 (1.9) | 2 (1.8) | 1 |
| < 40 mg/dL, No (%) | 0 (0) | 0 (0) | 1 |
| Hypoglycemia per samples |  |  |  |
| < 70 mg/dL, No (%) | 4 (0.4) | 3 (0.2) | 0.7 |
| < 60 mg/dL, No (%) | 2 (0.2) | 2 (0.2) | 1 |
| < 40 mg/dL, No (%) | 0 (0) | 0 (0) | 1 |
| **Workload** |  |  |  |
| Sampling interval, mean (SD), hours | 4.0 (1.3) | 2.5 (0.6) | <0.001 |

**Table S4: Blood glucose management in the two randomization groups for cardiac surgery**

| **Cardiac surgery** |  |  |  |
| --- | --- | --- | --- |
|  | **Nurse-C** | **LOGIC-C** | **P value** |
| Patients | 458 | 465 |  |
| Study period, median (IQR), days | 2 (2-3) | 2 (2-3) | 0.6 |
| **Efficacy** |  |  |  |
| Glycemic penalty index (GPI), median (IQR) | 18.1 (11.4-28.1) | 11.2 (6.4-16.3) | <0.001 |
| Blood glucose, mean (SD), mg/dL | 123 (23) | 114 (15) | <0.001 |
| Minimum blood glucose, mg/dL | 33 | 30 |  |
| Maximum blood glucose, mg/dL | 428 | 335 |  |
| Hyperglycemic index, median (IQR), mg/dL | 9 (4-18) | 4 (2-7) | <0.001 |
| Time in target range, median (IQR), percentage | 43.4 (24.3-62.8) | 64.2 (50.3-78.1) | <0.001 |
| Time to reach target range, median (IQR), hours | 3.2 (0-9.8) | 2.0 (0-4.6) | <0.001 |
| Mean of Maximum Delta Glycemia per day, median (IQR), mg/dL | 37 (28-53) | 32 (23-43) | <0.001 |
| **Safety** |  |  |  |
| Hypoglycemia per patient |  |  |  |
| < 70 mg/dL, No (%) | 73 (15.9) | 47 (10.1) | 0.01 |
| < 60 mg/dL, No (%) | 30 (6.6) | 13 (2.8) | 0.008 |
| < 40 mg/dL, No (%) | 2 (0.4) | 3 (0.6) | 1 |
| Hypoglycemia per samples |  |  |  |
| < 70 mg/dL, No (%) | 126 (1.8) | 94 (1.1) | <0.001 |
| < 60 mg/dL, No (%) | 41(0.6) | 30 (0.4) | 0.05 |
| < 40 mg/dL, No (%) | 2 (0.03) | 4 (0.05) | 0.9 |
| **Workload** |  |  |  |
| Sampling interval, mean (SD), hours | 2.8 (0.8) | 2.2 (0.4) | <0.001 |

**Table S5: Blood glucose management in the two randomization groups for non-cardiac surgery**

| **Non-cardiac surgery** |  |  |  |
| --- | --- | --- | --- |
|  | **Nurse-C** | **LOGIC-C** | **P value** |
| Patients | 315 | 312 |  |
| Study period, median (IQR), days | 4 (2-9) | 4 (3-7) | 0.6 |
| **Efficacy** |  |  |  |
| Glycemic penalty index (GPI), median (IQR) | 15.5 (9.4-24.3) | 10.1 (5.8-16.0) | <0.001 |
| Blood glucose, mean (SD), mg/dL | 115 (17) | 108 (15) | <0.001 |
| Minimum blood glucose, mg/dL | 21 | 26 |  |
| Maximum blood glucose, mg/dL | 386 | 511 |  |
| Hyperglycemic index, median (IQR), mg/dL | 7 (3-14) | 3 (1-7) | <0.001 |
| Time in target range, median (IQR), percentage | 53.2 (35.9-69.4) | 70.0 (57.2-81.8) | <0.001 |
| Time to reach target range, median (IQR), hours | 4.6 (0.2-8.9) | 2.5 (0.1-5.3) | <0.001 |
| Mean of Maximum Delta Glycemia per day, median (IQR), mg/dL | 36 (25-52) | 37 (27-50) | 0.9 |
| **Safety** |  |  |  |
| Hypoglycemia per patient |  |  |  |
| < 70 mg/dL, No (%) | 100 (31.7) | 102 (32.7) | 0.9 |
| < 60 mg/dL, No (%) | 48 (15.2) | 45 (14.4) | 0.8 |
| < 40 mg/dL, No (%) | 7 (2.2) | 4 (1.3) | 0.5 |
| Hypoglycemia per samples |  |  |  |
| < 70 mg/dL, No (%) | 220 (1.8) | 248 (1.8) | 0.7 |
| < 60 mg/dL, No (%) | 82 (0.7) | 75 (0.5) | 0.1 |
| < 40 mg/dL, No (%) | 7 (0.06) | 5 (0.04) | 0.6 |
| **Workload** |  |  |  |
| Sampling interval, mean (SD), hours | 3.2 (0.9) | 2.4 (0.5) | <0.001 |

**Table S6: Blood glucose management in the two randomization groups for medical reason of admission**

| **Medical reason of admission** |  |  |  |
| --- | --- | --- | --- |
|  | **Nurse-C** | **LOGIC-C** | **P value** |
| Patients | 131 | 164 |  |
| Study period, median (IQR), days | 5 (2-11) | 4 (3-7) | 0.3 |
| **Efficacy** |  |  |  |
| Glycemic penalty index (GPI), median (IQR) | 15.4 (9.0-25.0) | 10.2 (5.9-15.9) | <0.001 |
| Blood glucose, mean (SD), mg/dL | 117 (19) | 107 (16) | <0.001 |
| Minimum blood glucose, mg/dL | 21 | 28 |  |
| Maximum blood glucose, mg/dL | 375 | 511 |  |
| Hyperglycemic index, median (IQR), mg/dL | 7 (3-15) | 3 (1-6) | <0.001 |
| Time in target range, median (IQR), percentage | 51.1 (34.5-68.7) | 68.4 (55.7-81.8) | <0.001 |
| Time to reach target range, median (IQR), hours | 4.3 (0-9.1) | 2.1 (0-5.4) | <0.001 |
| Mean of Maximum Delta Glycemia per day, median (IQR), mg/dL | 37 (23-54) | 38 (27-51) | 1 |
| **Safety** |  |  |  |
| Hypoglycemia per patient |  |  |  |
| < 70 mg/dL, No (%) | 48 (36.6) | 58 (35.4) | 0.9 |
| < 60 mg/dL, No (%) | 25 (19.1) | 30 (18.3) | 0.9 |
| < 40 mg/dL, No (%) | 5 (3.8) | 3 (1.8) | 0.5 |
| Hypoglycemia per samples |  |  |  |
| < 70 mg/dL, No (%) | 112 (2.1) | 170 (2.4) | 0.3 |
| < 60 mg/dL, No (%) | 48 (0.9) | 60 (0.8) | 0.8 |
| < 40 mg/dL, No (%) | 5 (0.09) | 4 (0.05) | 0.7 |
| **Workload** |  |  |  |
| Sampling interval, mean (SD), hours | 3.5 (1.1) | 2.5 (0.5) | <0.001 |

**Table S7: Blood glucose management in the two randomization groups for non-medical reason of admission**

| **Non-medical reason of admission** |  |  |  |
| --- | --- | --- | --- |
|  | **Nurse-C** | **LOGIC-C** | **P value** |
| Patients | 642 | 613 |  |
| Study period, median (IQR), days | 2 (2-3) | 2 (2-3) | 0.2 |
| **Efficacy** |  |  |  |
| Glycemic penalty index (GPI), median (IQR) | 17.5 (10.8-26.7) | 11.0 (6.3-16.2) | <0.001 |
| Blood glucose, mean (SD), mg/dL | 120 (21) | 113 (15) | <0.001 |
| Minimum blood glucose, mg/dL | 33 | 26 |  |
| Maximum blood glucose, mg/dL | 428 | 354 |  |
| Hyperglycemic index, median (IQR), mg/dL | 8 (4-17) | 4 (1-7) | <0.001 |
| Time in target range, median (IQR), percentage | 46.2 (27.3-64.5) | 66.8 (51.5-79.8) | <0.001 |
| Time to reach target range, median (IQR), hours | 3.6 (0-9.3) | 2.2 (0-5.0) | <0.001 |
| Mean of Maximum Delta Glycemia per day, median (IQR), mg/dL | 36 (27-52) | 33 (23-45) | <0.001 |
| **Safety** |  |  |  |
| Hypoglycemia per patient |  |  |  |
| < 70 mg/dL, No (%) | 125 (19.5) | 91 (14.8) | 0.03 |
| < 60 mg/dL, No (%) | 53 (8.2) | 28 (4.6) | 0.008 |
| < 40 mg/dL, No (%) | 4 (0.6) | 4 (0.7) | 1 |
| Hypoglycemia per samples |  |  |  |
| < 70 mg/dL, No (%) | 234 (1.8) | 172 (1.1) | <0.001 |
| < 60 mg/dL, No (%) | 75 (0.6) | 45 (0.3) | <0.001 |
| < 40 mg/dL, No (%) | 4 (0.03) | 5 (0.03) | 0.9 |
| **Workload** |  |  |  |
| Sampling interval, mean (SD), hours | 2.9 (0.7) | 2.2 (0.5) | <0.001 |

**Table S8: Blood glucose management in the two randomization groups for sepsis on admission**

| **Sepsis on admission** |  |  |  |
| --- | --- | --- | --- |
|  | **Nurse-C** | **LOGIC-C** | **P value** |
| Patients | 102 | 129 |  |
| Study period, median (IQR), days | 5 (3-12) | 4 (2.5-9) | 0.2 |
| **Efficacy** |  |  |  |
| Glycemic penalty index (GPI), median (IQR) | 14.7 (10.8-24.6) | 9.7 (5.2-15.7) | <0.001 |
| Blood glucose, mean (SD), mg/dL | 116 (20) | 106 (14) | <0.001 |
| Minimum blood glucose, mg/dL | 31 | 34 |  |
| Maximum blood glucose, mg/dL | 375 | 511 |  |
| Hyperglycemic index, median (IQR), mg/dL | 7 (3-14) | 3 (1-6) | <0.001 |
| Time in target range, median (IQR), percentage | 53.3 (34.9-66.7) | 70.5 (59.0-82.0) | <0.001 |
| Time to reach target range, median (IQR), hours | 4.4 (0.09-9.4) | 1.9 (0-5.1) | 0.003 |
| Mean of Maximum Delta Glycemia per day, median (IQR), mg/dL | 37 (25-50) | 34 (23-47) | 0.4 |
| **Safety** |  |  |  |
| Hypoglycemia per patient |  |  |  |
| < 70 mg/dL, No (%) | 36 (35.3) | 43 (33.3) | 0.8 |
| < 60 mg/dL, No (%) | 23 (22.6) | 24 (18.6) | 0.5 |
| < 40 mg/dL, No (%) | 4 (3.9) | 2 (1.6) | 0.4 |
| Hypoglycemia per samples |  |  |  |
| < 70 mg/dL, No (%) | 100 (2.2) | 136 (2.2) | 1 |
| < 60 mg/dL, No (%) | 49 (1.1) | 47 (0.8) | 0.09 |
| < 40 mg/dL, No (%) | 4 (0.09) | 2 (0.03) | 0.4 |
| **Workload** |  |  |  |
| Sampling interval, mean (SD), hours | 3.3 (0.8) | 2.5 (0.5) | <0.001 |

**Table S9: Blood glucose management in the two randomization groups for non-sepsis on admission**

| **Non-sepsis on admission** |  |  |  |
| --- | --- | --- | --- |
|  | **Nurse-C** | **LOGIC-C** | **P value** |
| Patients | 671 | 648 |  |
| Study period, median (IQR), days | 2 (2-4) | 2 (2-3) | 0.6 |
| **Efficacy** |  |  |  |
| Glycemic penalty index (GPI), median (IQR) | 17.5 (10.6-26.8) | 11.1 (6.3-16.3) | <0.001 |
| Blood glucose, mean (SD), mg/dL | 120 (21) | 112 (15) | <0.001 |
| Minimum blood glucose, mg/dL | 21 | 26 |  |
| Maximum blood glucose, mg/dL | 428 | 394 |  |
| Hyperglycemic index, median (IQR), mg/dL | 8 (3-17) | 4 (2-7) | <0.001 |
| Time in target range, median (IQR), percentage | 46.2 (27.6-64.8) | 66.4 (51.6-79.7) | <0.001 |
| Time to reach target range, median (IQR), hours | 3.6 (0-9.3) | 2.2 (0-5.1) | <0.001 |
| Mean of Maximum Delta Glycemia per day, median (IQR), mg/dL | 36 (27-52) | 34 (24-46) | <0.001 |
| **Safety** |  |  |  |
| Hypoglycemia per patient |  |  |  |
| < 70 mg/dL, No (%) | 137 (20.4) | 106 (16.4) | 0.06 |
| < 60 mg/dL, No (%) | 55 (8.2) | 34 (5.2) | 0.04 |
| < 40 mg/dL, No (%) | 5 (0.7) | 5 (0.8) | 1 |
| Hypoglycemia per samples |  |  |  |
| < 70 mg/dL, No (%) | 246 (1.7) | 206 (1.3) | 0.002 |
| < 60 mg/dL, No (%) | 74 (0.5) | 58 (0.4) | 0.05 |
| < 40 mg/dL, No (%) | 5 (0.03) | 7 (0.04) | 0.9 |
| **Workload** |  |  |  |
| Sampling interval, mean (SD), hours | 2.9 (0.8) | 2.3 (0.5) | <0.001 |

**Table S10: Blood glucose management in the two randomization groups for infection on admission**

| **Infection on admission** |  |  |  |
| --- | --- | --- | --- |
|  | **Nurse-C** | **LOGIC-C** | **P value** |
| Patients | 219 | 217 |  |
| Study period, median (IQR), days | 5 (3-11) | 4 (3-9) | 0.05 |
| **Efficacy** |  |  |  |
| Glycemic penalty index (GPI), median (IQR) | 14.4 (9.4-23.8) | 10.2 (6.1-15.9) | <0.001 |
| Blood glucose, mean (SD), mg/dL | 115 (18) | 107 (15) | <0.001 |
| Minimum blood glucose, mg/dL | 31 | 26 |  |
| Maximum blood glucose, mg/dL | 375 | 511 |  |
| Hyperglycemic index, median (IQR), mg/dL | 7 (3-13) | 3 (1-7) | <0.001 |
| Time in target range, median (IQR), percentage | 53.1 (36.4-68.5) | 68.7 (57.9-79.9) | <0.001 |
| Time to reach target range, median (IQR), hours | 3.9 (0-8.8) | 2.4 (0.1-5.3) | 0.006 |
| Mean of Maximum Delta Glycemia per day, median (IQR), mg/dL | 36 (25-53) | 37 (27-50) | 0.7 |
| **Safety** |  |  |  |
| Hypoglycemia per patient |  |  |  |
| < 70 mg/dL, No (%) | 75 (34.2) | 73 (33.6) | 0.9 |
| < 60 mg/dL, No (%) | 38 (17.4) | 33 (15.2) | 0.6 |
| < 40 mg/dL, No (%) | 5 (2.3) | 4 (1.8) | 1 |
| Hypoglycemia per samples |  |  |  |
| < 70 mg/dL, No (%) | 182 (1.9) | 193 (1.9) | 1 |
| < 60 mg/dL, No (%) | 70 (0.7) | 60 (0.6) | 0.2 |
| < 40 mg/dL, No (%) | 5 (0.05) | 5 (0.05) | 0.9 |
| **Workload** |  |  |  |
| Sampling interval, mean (SD), hours | 3.3 (0.9) | 2.5 (0.5) | <0.001 |

**Table S11: Blood glucose management in the two randomization groups for non-infections on admission**

| **Non-infections on admission** |  |  |  |
| --- | --- | --- | --- |
|  | **Nurse-C** | **LOGIC-C** | **P value** |
| Patients | 554 | 560 |  |
| Study period, median (IQR), days | 2 (2-3) | 2 (2-3) | 0.7 |
| **Efficacy** |  |  |  |
| Glycemic penalty index (GPI), median (IQR) | 18.3 (11.1-27.5) | 11.1 (6.2-16.3) | <0.001 |
| Blood glucose, mean (SD), mg/dL | 121 (22) | 113 (15) | <0.001 |
| Minimum blood glucose, mg/dL | 21 | 30 |  |
| Maximum blood glucose, mg/dL | 428 | 345 |  |
| Hyperglycemic index, median (IQR), mg/dL | 9 (4-18) | 4 (2-7) | <0.001 |
| Time in target range, median (IQR), percentage | 44.7 (25.0-64.3) | 65.8 (51.0-80.2) | <0.001 |
| Time to reach target range, median (IQR), hours | 3.6 (0 – 9.5) | 2.1 (0-5) | <0.001 |
| Mean of Maximum Delta Glycemia per day, median (IQR), mg/dL | 37 (27-52) | 33 (23-45) | <0.001 |
| **Safety** |  |  |  |
| Hypoglycemia per patient |  |  |  |
| < 70 mg/dL, No (%) | 98 (17.7) | 76 (13.6) | 0.07 |
| < 60 mg/dL, No (%) | 40 (7.2) | 25 (4.5) | 0.05 |
| < 40 mg/dL, No (%) | 4 (0.7) | 3 (0.5) | 0.7 |
| Hypoglycemia per samples |  |  |  |
| < 70 mg/dL, No (%) | 164 (1.7) | 149 (1.2) | 0.002 |
| < 60 mg/dL, No (%) | 53 (0.6) | 45 (0.4) | 0.04 |
| < 40 mg/dL, No (%) | 4 (0.04) | 4 (0.03) | 1 |
| **Workload** |  |  |  |
| Sampling interval, mean (SD), hours | 2.8 (0.8) | 2.2 (0.5) | <0.001 |

**Table S12: Blood glucose management in the two randomization groups for known diabetes mellitus on admission**

| **Diabetes mellitus on admission** |  |  |  |
| --- | --- | --- | --- |
|  | **Nurse-C** | **LOGIC-C** | **P value** |
| Patients | 167 | 168 |  |
| Study period, median (IQR), days | 2 (2-3) | 2.5 (2-4) | 0.3 |
| **Efficacy** |  |  |  |
| Glycemic penalty index (GPI), median (IQR) | 23.1 (14.3-32.0) | 15.3 (9.8-20.6) | <0.001 |
| Blood glucose, mean (SD), mg/dL | 126 (27) | 114 (19) | <0.001 |
| Minimum blood glucose, mg/dL | 21 | 30 |  |
| Maximum blood glucose, mg/dL | 416 | 364 |  |
| Hyperglycemic index, median (IQR), mg/dL | 12 (6-21) | 6 (3-10) | <0.001 |
| Time in target range, median (IQR), percentage | 42.3 (25.9-56.9) | 59.4 (45.7-73.2) | <0.001 |
| Time to reach target range, median (IQR), hours | 4.7 (1.0-10.4) | 3.7 (1.5-6.8) | 0.02 |
| Mean of Maximum Delta Glycemia per day, median (IQR), mg/dL | 48 (35-68) | 41 (32-57) | 0.003 |
| **Safety** |  |  |  |
| Hypoglycemia per patient |  |  |  |
| < 70 mg/dL, No (%) | 51 (30.5) | 44 (26.2) | 0.4 |
| < 60 mg/dL, No (%) | 19 (11.4) | 20 (11.9) | 1 |
| < 40 mg/dL, No (%) | 2 (1.2) | 3 (1.8) | 1 |
| Hypoglycemia per samples |  |  |  |
| < 70 mg/dL, No (%) | 83 (2.4) | 98 (2.1) | 0.4 |
| < 60 mg/dL, No (%) | 26 (0.7) | 33 (0.7) | 0.9 |
| < 40 mg/dL, No (%) | 2 (0.06) | 4 (0.09) | 0.7 |
| **Workload** |  |  |  |
| Sampling interval, mean (SD), hours | 2.7 (0.8) | 2.2 (0.4) | <0.001 |

**Table S13: Blood glucose management in the two randomization groups for not known diabetes on admission**

| **Not known diabetes on admission** |  |  |  |
| --- | --- | --- | --- |
|  | **Nurse-C** | **LOGIC-C** | **P value** |
| Patients | 606 | 609 |  |
| Study period, median (IQR), days | 3 (2-5) | 2 (2-4) | 0.3 |
| **Efficacy** |  |  |  |
| Glycemic penalty index (GPI), median (IQR) | 16.0 (9.8-25.0) | 9.7 (5.6-14.8) | <0.001 |
| Blood glucose, mean (SD), mg/dL | 118 (19) | 111 (14) | <0.001 |
| Minimum blood glucose, mg/dL | 31 | 26 |  |
| Maximum blood glucose, mg/dL | 428 | 511 |  |
| Hyperglycemic index, median (IQR), mg/dL | 7 (3-15) | 3 (1-6) | <0.001 |
| Time in target range, median (IQR), percentage | 48.8 (29.3-68.5) | 68.6 (55.3-81.8) | <0.001 |
| Time to reach target range, median (IQR), hours | 3.3 (0-9.0) | 1.7 (0-4.6) | <0.001 |
| Mean of Maximum Delta Glycemia per day, median (IQR), mg/dL | 34 (24-48) | 32 (22-44) | 0.003 |
| **Safety** |  |  |  |
| Hypoglycemia per patient |  |  |  |
| < 70 mg/dL, No (%) | 122 (20.1) | 105 (17.2) | 0.2 |
| < 60 mg/dL, No (%) | 59 (9.7) | 38 (6.2) | 0.03 |
| < 40 mg/dL, No (%) | 7 (1.2) | 4 (0.7) | 0.4 |
| Hypoglycemia per samples |  |  |  |
| < 70 mg/dL, No (%) | 263 (1.7) | 244 (1.4) | 0.02 |
| < 60 mg/dL, No (%) | 97 (0.6) | 72 (0.4) | 0.006 |
| < 40 mg/dL, No (%) | 7 (0.05) | 5 (0.03) | 0.6 |
| **Workload** |  |  |  |
| Sampling interval, mean (SD), hours | 3.0 (0.9) | 2.3 (0.5) | <0.001 |

**Table S14: Blood glucose management in the two randomization groups for 80-110 mg/dL blood glucose target range**

| **80-110 mg/dL** |  |  |  |
| --- | --- | --- | --- |
|  | **Nurse-C** | **LOGIC-C** | **P value** |
| Patients | 668 | 667 |  |
| Study period, median (IQR), days | 3 (2-5) | 3 (2-5) | 0.7 |
| **Efficacy** |  |  |  |
| Glycemic penalty index (GPI), median (IQR) | 17.0 (10.7-25.4) | 11.3 (6.8-16.2) | <0.001 |
| Blood glucose, mean (SD), mg/dL | 115 (16) | 108 (11) | <0.001 |
| Minimum blood glucose, mg/dL | 21 | 26 |  |
| Maximum blood glucose, mg/dL | 428 | 511 |  |
| Hyperglycemic index, median (IQR), mg/dL | 8 (3-16) | 4 (2-7) | <0.001 |
| Time in target range, median (IQR), percentage | 48.2 (30.9-64.5) | 66.1 (52.1-77.9) | <0.001 |
| Time to reach target range, median (IQR), hours | 4.0 (0.02-9.2) | 2.4 (0-5.1) | <0.001 |
| Mean of Maximum Delta Glycemia per day, median (IQR), mg/dL | 36 (27-50) | 33 (24-45) | <0.001 |
| **Safety** |  |  |  |
| Hypoglycemia per patient |  |  |  |
| < 70 mg/dL, No (%) | 169 (25.3) | 146 (21.9) | 0.2 |
| < 60 mg/dL, No (%) | 76 (11.4) | 56 (8.4) | 0.08 |
| < 40 mg/dL, No (%) | 9 (1.3) | 7 (1.0) | 0.8 |
| Hypoglycemia per samples |  |  |  |
| < 70 mg/dL, No (%) | 342 (1.9) | 339 (1.6) | 0.02 |
| < 60 mg/dL, No (%) | 121 (0.7) | 103 (0.5) | 0.02 |
| < 40 mg/dL, No (%) | 9 (0.05) | 9 (0.04) | 0.9 |
| **Workload** |  |  |  |
| Sampling interval, mean (SD), hours | 2.8 (0.6) | 2.3 (0.4) | <0.001 |

**Table S15: Blood glucose management in the two randomization groups for 90-145 mg/dL blood glucose target range**

| **90-145 mg/dL** |  |  |  |
| --- | --- | --- | --- |
|  | **Nurse-C** | **LOGIC-C** | **P value** |
| Patients | 105 | 110 |  |
| Study period, median (IQR), days | 1 (1-2) | 2 (1-2) | 0.5 |
| **Efficacy** |  |  |  |
| Glycemic penalty index (GPI), median (IQR) | 21.7 (10.3-34.3) | 6.6 (0.7-15.9) | <0.001 |
| Blood glucose, mean (SD), mg/dL | 150 (24) | 134 (19) | <0.001 |
| Minimum blood glucose, mg/dL | 50 | 41 |  |
| Maximum blood glucose, mg/dL | 362 | 394 |  |
| Hyperglycemic index, median (IQR), mg/dL | 12 (3-24) | 2 (0-8) | <0.001 |
| Time in target range, median (IQR), percentage | 40.2 (15.5-70.4) | 77.3 (54.9-96.0) | <0.001 |
| Time to reach target range, median (IQR), hours | 0 (0-10.8) | 0 (0-4.1) | 0.2 |
| Mean of Maximum Delta Glycemia per day, median (IQR), mg/dL | 49 (25-68) | 38 (29-53) | 0.1 |
| **Safety** |  |  |  |
| Hypoglycemia per patient |  |  |  |
| < 70 mg/dL, No (%) | 4 (3.8) | 3 (2.7) | 0.7 |
| < 60 mg/dL, No (%) | 2 (1.9) | 2 (1.8) | 1 |
| < 40 mg/dL, No (%) | 0 (0) | 0 (0) | 1 |
| Hypoglycemia per samples |  |  |  |
| < 70 mg/dL, No (%) | 4 (0.4) | 3 (0.2) | 0.7 |
| < 60 mg/dL, No (%) | 2 (0.2) | 2 (0.2) | 1 |
| < 40 mg/dL, No (%) | 0 (0) | 0 (0) | 1 |
| **Workload** |  |  |  |
| Sampling interval, mean (SD), hours | 4.0 (1.3) | 2.5 (0.6) | <0.001 |

**Table S16: Incidence of new infections in the two randomization groups, according to the predefined subgroups**

| Incidence of new infections in the ICU, No (%) | **Nurse-C** | **LOGIC-C** | **P value** |
| --- | --- | --- | --- |
| Total population | 117/773 (15.14) | 104/777 (13.38) | 0.35 |
| Cardiac surgery | 25/458  (5.46) | 25/465  (5.38) | 1.00 |
| Medical admission | 40/131 (30.53) | 41/164  (25) | 0.30 |
| Sepsis on admission | 34/102 (33.33) | 26/129 (20.16) | 0.034 |
| Infection on admission | 70//219 (31.96) | 50//217 (23.04) | 0.042 |
| Known diabetes mellitus | 20/167 (11.98) | 17/168 (10.12) | 0.61 |
