# Supplementary material for: Software-guided versus nurse-directed blood glucose control in critically ill patients: the LOGIC-2 multicenter randomized controlled clinical trial
Source: Crit Care. 2017 Aug 14;21:212. doi: 10.1186/s13054-017-1799-6 (PMC5557320; doi:10.1186/s13054-017-1799-6)
Supplement: Supplementary file 2 — Study protocol: Competent Authority submission. (PDF 8786 kb) [file 13054_2017_1799_MOESM2_ESM.pdf]

Request for a randomised, controlled multi-centre intervention study:

Nurse-directed versus algorithm-assisted tight blood glucose control in adult critically ill patients: the LOGIC-2 multi-centre randomised controlled trial

Ethics Committee number: ML9517

Belgian Number: B322201318068

KULeuven number:S55613

Class IIb, Non-Implantable, Non-Invasive Medical Device

Principal Investigators:

Prof. Dr. Dieter Mesotten - KU Leuven, Leuven (BE) - Coordinating principal investigator
Prof. Dr. Greet Van den Berghe - KU Leuven, Leuven (BE)
Prof. Dr. Alexander Wilmer - KU Leuven, Leuven (BE) - Co-investigator
Dr. Jasperina Dubois - Jessa Hospital, Hasselt (BE)
Dr. Aimé Van Assche - Jessa Hospital, Hasselt (BE) - Co-investigator
Prof. Dr. Marcus Schultz - Academic Medical Center (AMC), Amsterdam (NL)
Dr. Roosmarijn van Hooijdonk - Academic Medical Center (AMC), Amsterdam (NL) (Co-investigator)

Daily management:

Prof. Dr. Dieter Mesotten [1]
Dr. ing. Tom Van Herpe [1,2] - KU Leuven, Leuven (BE)
MSc. Guy Veraghtert [1,2] - KU Leuven, Leuven (BE)

Advisors:

Prof. Dr. Greet Van den Berghe [1]
Prof. Dr. ir. Bart De Moor [2]

(1) KU Leuven
Dept. Intensive Care Medicine - University Hospitals Leuven
Herestraat 49
B-3000 Leuven
Belgium
{dieter.mesotten, greet.vandenberghe}@med.kuleuven.be
(2) KU Leuven
Dept. Electrical Engineering Department (ESAT-STADIUS)
Kasteelpark Arenberg 10
B-3001 Heverlee
Belgium
{tom.vanherpe, guy.veraghtert, bart.demoor}@esat.kuleuven.be

Table of Contents

Introduction 5

1. General information 8

1.1 Sponsor name 8

1.2 – 1.3 Number of submissions 8

1.4 Other Member States and non European countries 8

1.5 Signed statement 8

1.6 Copy of the Ethics committee opinion 9

2. INVESTIGATOR BROCHURE: Identification 10

2.1 Details allowing device to be identified 10

2.2 – 2.3 – 2.4 Trade name / Generic / Model name of device. 11

2.5 Model number 11

3. INVESTIGATOR BROCHURE: Other Device Details 12

3.1 Classification 12

3.2 Description of the intended clinical performance (ISO 14155). 12

3.3 Device description 12

3.4 Identification of any features of design that are different from a previously similar marketed product (if relevant). 14

3.5 Details of any new or previously untested features of the device 14

3.6 Clinical data on device in question 19

3.7 Benefit/Risk analysis 22

3.8 Summary and analysis of pre-clinical testing 22

3.9 Description of materials coming into contact with the body 23

3.10 Description of biocompatibility and biological safety 23

3.11 Identification of any pharmacological components of device 24

3.12 Design drawings 24

3.13 Description of software 24

3.14 Method of sterilisation, validation, cleaning, disinfection, … 26

3.15 Identification of any tissues of animal origin 26

3.16 Identification of any special manufacturing conditions required 26
3.17 List of relevant Standards 26
3.18 Instructions for use including risks, contra indications and warnings 27
3.19 Provisions for the recovering of the device and prevention of unauthorised use 28
4. CLINICAL INVESTIGATION PLAN: General information 29
4.1 Investigator team 29
4.2 – 4.3 Institution, Sponsor, Manufacturer 35
4.4 Synopsis of the clinical investigation plan 36
4.5 Summary of training experience 40
4.7 Informed consent 41
4.8 Case Report Form (CRF) 41
4.9 Clinical study 41
5. CLINICAL INVESTIGATION PLAN: Investigation Parameters and Design 42
5.1 Objectives of clinical investigation 42
5.2 Investigation design 42
5.3 Numbers of subjects 44
5.4 Duration of study 45
5.5 Criteria for subject selection 45
5.6 Criteria for withdrawal from study 46
5.7 Description and justification of hazards caused by invasive procedures that are not medically required (if applicable). 46
5.8 Description of general methods of diagnosis or treatment of the medical condition for which the investigation testing is being proposed. 46
5.9 Monitoring arrangements during the clinical investigation including request for direct access to source documents including extent of source data verification 46
6. CLINICAL INVESTIGATION PLAN: Data Collection/Analysis/Statistics 48
6.1 Description of endpoints 48
6.2 Description and justification of statistical design, method and analytical procedures 49

6.3 Procedures for data collection, review, cleaning, and issuing and resolving queries, if appropriate. ... 53

6.4 Recording and reporting procedures of clinical investigation plan deviations, if appropriate ... 56

7. CLINICAL INVESTIGATION PLAN: Safety Reporting ... 57

7.1 Definitions of adverse events and adverse device effects ... 57

7.2 Definitions of serious adverse events and serious adverse device effects ... 57

7.3 Details of procedures of those events to be reported to the relevant Ethics ... 58

Committees and Competent Authority, including timelines ... 58

7.4 List of foreseeable adverse events and adverse device effects, likely incidence, mitigation and/or treatment. ... 59

7.5 Emergency contact details for reporting serious adverse events and serious adverse device effects to the Sponsor. ... 60

8. CLINICAL INVESTIGATION PLAN : Other ... 61

8.1 Reference to Insurance coverage in case of injury ... 61

8.2 Device accountability procedures ... 61

8.3 Ethics and informed consent procedures ... 62

8.4 Procedure for early termination or suspension of the investigation giving criteria and risk analysis ... 63

8.5 Procedure for clinical investigation plan amendments ... 63

8.6 Final report and publication policy. ... 63

Appendix 1: Signed statement

Appendix 2: Copy of the Ethics committee approval

Appendix 3: Risk management analysis

Appendix 4: Incorporation of human blood

Appendix 5: Dutch Manual

Appendix 6: Identification of any tissues of animal origin

Appendix 7: Informed consent (UZ Leuven)

Appendix 8: Informed consent (Jessa Hospital Hasselt)

Appendix 9: Insurance document

Appendix 10: Notification fee payment

References

Introduction

While stress hyperglycaemia has traditionally been regarded as an adaptive, beneficial response, it is clear from observational studies that hyperglycaemia, as well as hypoglycaemia, are associated with increased risk of death in critically ill patients. The association between blood glucose levels and mortality risk follows a J-curved relationship with the nadir roughly between 80-140 mg/dL (4.4-7.8 mmol/L). However, in order to show a causal relationship between hyperglycaemia and mortality risk, randomised controlled trials (RCT) that target and achieve different blood glucose levels are required[1-3].

The first RCT that lowered blood glucose levels by insulin therapy during critical illness was performed in Leuven[4]. As one would expect from a seminal study, it had a proof-of-concept design: single centre, a homogeneous patient population (mainly cardiac surgery and high risk/complicated non-cardiac surgery), standardised arterial blood glucose measurements and central venous continuous insulin infusion using an accurate syringe pump. The study targeted a “strictly normal level for fasting blood glucose”, i.e. 80-110 mg/dL (4.4-6.1 mmol/L) versus treating hyperglycaemia only when it exceeded the renal threshold of 215 mg/dL (12.0 mmol/L). The insulin dose-adaptations were based on a guideline to stimulate intuitive and anticipating decision making by bedside nurses. Extensive training sessions were organised to optimise the execution of tight glycaemic control. The study showed that maintaining strict normoglycaemia by “intensive insulin therapy” lowered mortality in the intensive care unit (ICU) from 8.0% to 4.6% (absolute risk reduction (ARR) 3.4%) and in-hospital mortality from 10.9% to 7.2% (ARR 3.7%). It also reduced morbidity by preventing organ failure. This was reflected in a shorter duration of mechanical ventilation, a decreased incidence of acute kidney failure, severe infections and polyneuropathy and less blood transfusions.

Generalising to the medical and paediatric critically ill patient population, these results were confirmed in the same context of a well-controlled single centre expert setting[5,6] and in implementation studies[7-11]. In contrast, large multi-centre studies (VISEP, GLUCONTROL and NICE-SUGAR) could not confirm this survival benefit[12-14].

Besides the differences in the target level for blood glucose in the control groups (much lower for GLUCONTROL and NICE-SUGAR than in the Leuven trials), methodological disparity in the execution of the complex intervention of tight glycaemic control may have contributed significantly to the contradicting results[15,16]. Firstly, allowing inaccurate blood glucose measurement devices, in combination with different blood sampling sites and types of infusion pumps, may have led to unnoticed swings in blood glucose levels. Secondly, the level of expertise of the intensive care nurses with the therapy may have been variable due to low study patient numbers per centre. Hence, these multi-centre trials failed to reach the predefined blood glucose targets in the intervention group, resulting in a significant overlap with the control group.

Now everyone acknowledges that the implementation and evaluation of complex interventions is a stepwise, gradual process that consumes vast amounts of time and resources [17]. And clearly in the case of tight glycaemic control the fringe conditions were overlooked, when this complex intervention made the step from proof-of-concept study to large multi-centre, effectiveness trials. The scientific community has hinted that the focus of future studies should shift to the accuracy of blood glucose measurements[18], glycaemic management protocols [19] and the validation of markers of quality of glycaemic control[20-23].

Besides the issue of efficacy in steering blood glucose levels in the predefined target range, strategies to avoid hypoglycaemia, inherently associated with tight glycaemic control, are a burning question[24]. The incidence of severe hypoglycaemia < 40 mg/dL (2.2 mmol/L) increased in all studies 5-13 fold[16]. Even in an experienced centre the incidence of hypoglycaemia raised to 11.3% in the adult ICU population[25] and 25% in the paediatric critically ill patients[6]. Severe or prolonged hypoglycaemia can cause convulsions, coma, and irreversible brain damage as well as cardiac arrhythmias. The risk of hypoglycaemia is a concern when intensive insulin therapy is implemented in the ICU because early hypoglycaemic symptoms are not easily recognised in sedated critically ill patients. However, provided that hypoglycaemia is diagnosed and treated promptly and adequately, the latter implying prevention of over-correction, the benefit of preventing hyperglycaemia in ICU patients seems to outweigh the short-term risk of hypoglycaemia[26]. In the studies with less attention to the methodological aspects and implementation process of tight glycaemic control the association between hypoglycaemia and poor outcome is more pronounced[27,28].

Computerised algorithms may improve glycaemic control and lower hypoglycaemic episodes[29,30]. This computer-assisted blood glucose control software may in the future be combined with accurate, continuous blood glucose monitoring devices, preferably in closed-loop systems to avoid hypoglycaemia and large blood glucose swings[31].

The LOGIC (Lova Glucose control for Intensive Care) - Insulin algorithm is such a computer-assisted blood glucose control software. It was developed at the KU Leuven and advises the nurse on the insulin dosage (or a dextrose bolus in case of hypoglycaemia) as well as on the next blood sampling interval. The LOGIC-Insulin control system is founded on a robust, biphasic and adaptive patient model comprising two main phase-I variables (patient profile and on-admission variables) for the initial phase and five main phase-II variables (patient profile, blood glucose, insulin dose sequence, nutrition and steroid drugs) for the second phase. The patient profile is defined by the reason for ICU admission, the prior history of diabetes and the body mass index, whereas the on-admission variables are set by the severity of illness, the blood glucose level and the nutrition, all on admission. Further, the model coefficients corresponding to the phase-II variables are adapted based on the incoming closed-loop measurements (every sampling episode) and, if appropriate, on an internal glucose control performance evaluation system (every 24 hours). This control system assesses the level of blood glucose control and the required blood sampling frequency, in the previous 24 hours. Visual

alarms on sampling time, hypoglycaemia and nutrition dose entry errors are also included in the software.

In the first step of clinical validation, the LOGIC-Insulin algorithm was tested in a single-centre randomised controlled trial with 300 patients[32]. In this study the LOGIC-Insulin algorithm improved the efficacy of blood glucose control (avoiding persistent hyperglycaemia) without increasing the rate of hypoglycaemia in comparison with blood glucose control by the expert Leuven nursing team. These results are described in more detail further in this document. This study was only the first step in the clinical validation. Due to its single-centre design, the external validity and generalisability of the LOGIC-1 results are lower.

Therefore, the research proposal presented in this document describes the request for the LOGIC-2 study in which the improved LOGIC-Insulin software will be tested in a large, pragmatic multi-centre clinical trial. As in the LOGIC-1 trial, two manners of intensive insulin therapy in the ICU are compared: *glucose control by the bedside nurse* versus *glucose control by an advising computer algorithm*. However, in LOGIC-2 the study will take place *in three different hospitals*: UZ Leuven (Belgium), Jessa Hospital (Hasselt) and Academic Medical Center (AMC Amsterdam, The Netherlands). Also, to comply with recent recommendations on blood glucose control, *two different target ranges* will be included in the software[31]. The study is statistically powered to detect differences in the incidence of hypoglycaemia, as this is the major concern of intensive care nurses and physicians. This document is structured based on the European Commission formulations as described in the “Guidelines on medical devices: Guide for competent authorities in making an assessment of clinical investigation notification” (MEDDEV 2.7/2, December 2008).

1. General information

1.1 Sponsor name

The sponsor of this study is the KU Leuven:

KU Leuven
Leuven Research & Development
Waaistraat 6
B-3000 Leuven
Belgium

The contact person is Prof. Dr. Dieter Mesotten:

Dieter Mesotten, MD, PhD
KU Leuven
Dept. Intensive Care Medicine
University Hospitals Leuven
Herestraat 49
B-3000 Leuven
Belgium
Tel: +32 16 344021
Fax: +32 16 344015
dieter.mesotten@med.kuleuven.be

1.2 – 1.3 Number of submissions

This is the first submission of the LOGIC-2 study.

1.4 Other Member States and non European countries

This randomised, controlled multicentre intervention study will take place in Belgium and the Netherlands.

1.5 Signed statement

The medical software presented here for the scope of this study complies with the essential clinical requirements, except with regard to the aspect of the software which will be investigated (i.e. the performance of the medical device with respect to blood glucose control). Every precaution has been taken to protect the health and safety of the patient. The medical device gives an insulin dose *advice* to the nurse which can be simply overruled. Further, an *alarm* system is included in order to warn the nurse when a blood sample should be taken. Finally, the system also advises the nurse to administer a bolus of *glucose* calories in case of (potentially dangerous) hypoglycaemic episodes.
The signed statement is added in **Appendix 1**.

1.6 Copy of the Ethics committee opinion

A copy of the Ethics committee's approval for the presented study is added in **Appendix 2**.

2. INVESTIGATOR BROCHURE: Identification

2.1 Details allowing device to be identified

The medical device under study is an improved version of the software that was tested in the LOGIC-1 study. The new software comprises a sophisticated algorithm to normalise the blood glucose levels of critically ill patients with an adjustable blood glucose target range and a modern object-oriented graphical user interface, meeting European legislation criteria regarding software development and testing.

The algorithm computes the insulin flow each time a new blood glucose value is available or when important changes regarding the type or amount of nutrition calories are expected. The algorithm aims at reaching normoglycaemia as quickly as possible, avoiding hypo- and hyperglycaemic episodes and avoiding high blood glucose variability (i.e. blood glucose swings). Although the main part of the algorithm was kept constant (as glucose control in LOGIC-1 study was found to be effective and efficient), specific parts of the algorithm were further improved based on the glucose trajectories observed in the LOGIC-1 study. Further, compared to the previous version, the algorithm has now an adjustable blood glucose target range as no general consensus on the 'ideal' blood glucose target range exists (e.g. 80-110 mg/dL (4.4-6.1 mmol/L) for UZ Leuven and Jessa Hospital Hasselt, 90-145 mg/dL (5.0-8.0 mmol/L) for AMC Amsterdam). Like the previous version, the new algorithm gives an advice on insulin dose and glucose bolus. As the device is still under study as well as for liability reasons, confirmation by the nurse is mandatory. Based on expertise, the nurse evaluates the suggested insulin dose / glucose bolus and may overrule if appropriate.

Next, the confirmed insulin dose is administered to the patient and the software computes the time instant when the next blood sample (i.e. the next glucose measurement) is expected. An alarm system is activated when the waiting period for the next sample has been passed.

The new LOGIC-Insulin graphical user interface (Figure 1) is now coded into a modern object-oriented code language (Java) by a professional IT-expert (Mr. Guy Veraghtert). The previous version was coded in the engineering language Matlab and meant for research study purpose only. The design of the interface is further optimized by the gathered feedback of the ICU nurses from the three different centres with whom we closely collaborate.

In the context of future CE-mark approval for the medical software under study it is mandatory that this reprogramming of the LOGIC-Insulin software also includes a quality system, which encompasses all procedures for software design and distribution. During the Java software development process the harmonised standard IEC62304 has been followed. Further, ISO-norm 14971 has formed the base of the risk management procedures applicable in the new software. All regulatory aspects have been handled under guidance of Mr. Peter Frederickx from the consultancy company Think & Do,

Klappijstraat 29, B-3294 Diest, Belgium. Further details of the algorithm and the corresponding software are presented below.

2.2-2.3-2.4 Trade name / Generic / Model name of device.

The name of the algorithm and the corresponding graphical user interface is LOGIC-Insulin (**Lo**va **G**lucose control for **I**ntensive **C**are). At the time of filing no differentiation with respect to the trade name, generic name and model name exists.

2.5 Model number

"LOGIC-Insulin - version 2"

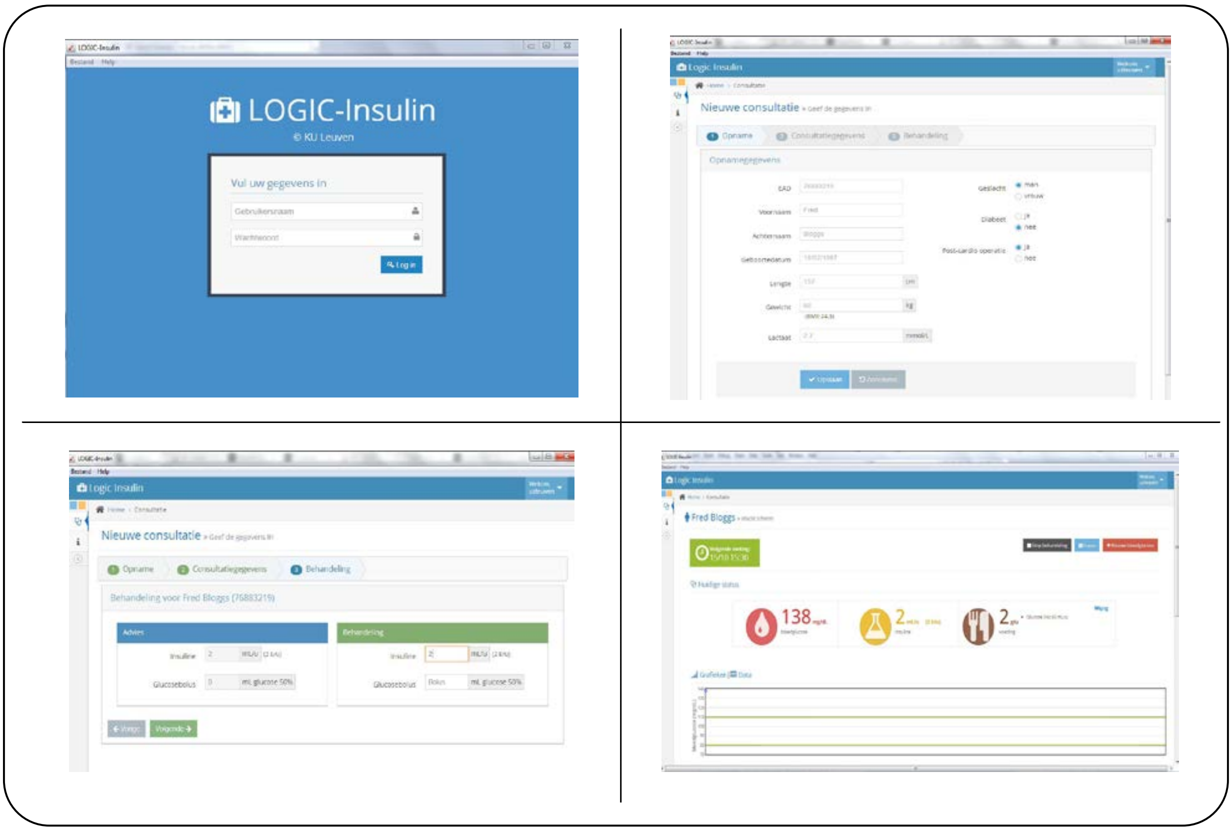

Figure 1. Screenshots of the new version of the LOGIC-Insulin software. LOGIC-Insulin advises the nurses on the insulin flow or glucose bolus (only in case of hypoglycaemic events).

3. INVESTIGATOR BROCHURE: Other Device Details

3.1 Classification

Based on the medical device directives 93/42/EEC and 2.4/1 rev.9, the LOGIC-Insulin software will resort under a **class IIb**: *Software intended to be used to administer and/or remove medicines … that is potentially hazardous … This would apply for instance to software used for calculating delivery dosages.*

3.2 Description of the intended clinical performance (ISO 14155).

The intended clinical performance of the medical device under study is the normalisation of the blood glucose levels in critically ill patients by administering the appropriate insulin dose sequences. It is the aim of the device to compute the most optimal insulin dosage (or glucose bolus in the event of hypoglycaemia) such that hypoglycaemic or hyperglycaemic episodes are avoided.

3.3 Device description

The concept of controlling blood glucose levels in critically ill patients comprises three main parts:

- a **sensor** that produces a signal indicative of the blood glucose level in a critically ill patient:
  In this study, arterial blood glucose levels will be exclusively measured with an on-site blood gas analyser: the ABL 90 Flex blood gas analyser (Radiometer) for UZ Leuven, the Rapid Point 500 blood gas analyser (Siemens) for Jessa Hospital Hasselt and the Rapid Lab 1265 blood gas analyser (Siemens) for AMC Amsterdam.

- an accurate programmable **actuator** delivers insulin to the patient at an administration rate:
  The Perfuser Space syringe pump from B. Braun will be used in UZ Leuven and AMC Amsterdam, whereas the Alaris GH Plus syringe pump from Carefusion will be used in Jessa Hospital Hasselt. Insulin is the blood glucose regulator. Human soluble insulin (Actrapid - NovoNordisk for UZ Leuven and Jessa Hospital Hasselt; Novorapid – NovoNordisk for AMC Amsterdam) is selected for this study.

- a **control system** regulates the administration rate of insulin. The medical device that forms the scope of the proposed RCT is the LOGIC-Insulin software which is medical software to regulate blood glucose levels of critically ill patients in a predefined (narrow) target range. This software system (including the algorithm) and the corresponding IT environment are further described in detail.

We opted to disconnect the software system under study from the PDMS (Patient Database Management System) which serves as database for clinical patient data. As much as possible LOGIC-Insulin is aiming to be *independent of* and *non-influencing* current clinical practice. Therefore the patient data that are necessary inputs for LOGIC-Insulin (e.g. nutrition calories) need to be manually submitted by the nurse. Next, the LOGIC-Insulin algorithm computes the insulin dose (or

glucose bolus in case of hypoglycaemia) which is defined as an ‘advice’. In a following step, this advised insulin dose needs to be confirmed by the nurse after which he/she manually adapts (if appropriate) the insulin flow of the insulin infusion pump. Finally, the timing for the next blood sampling is calculated and suggested to the nurse.

A general overview of the full system is presented in Figure 2. The accessories necessary for the study can be listed as follows:

- a bedside computer, connected to the LOGIC-Insulin cloud or server, for running the LOGIC-Insulin algorithm,
- a blood glucose sensor,
- a syringe pump,
- a blood glucose regulator: Human soluble insulin.

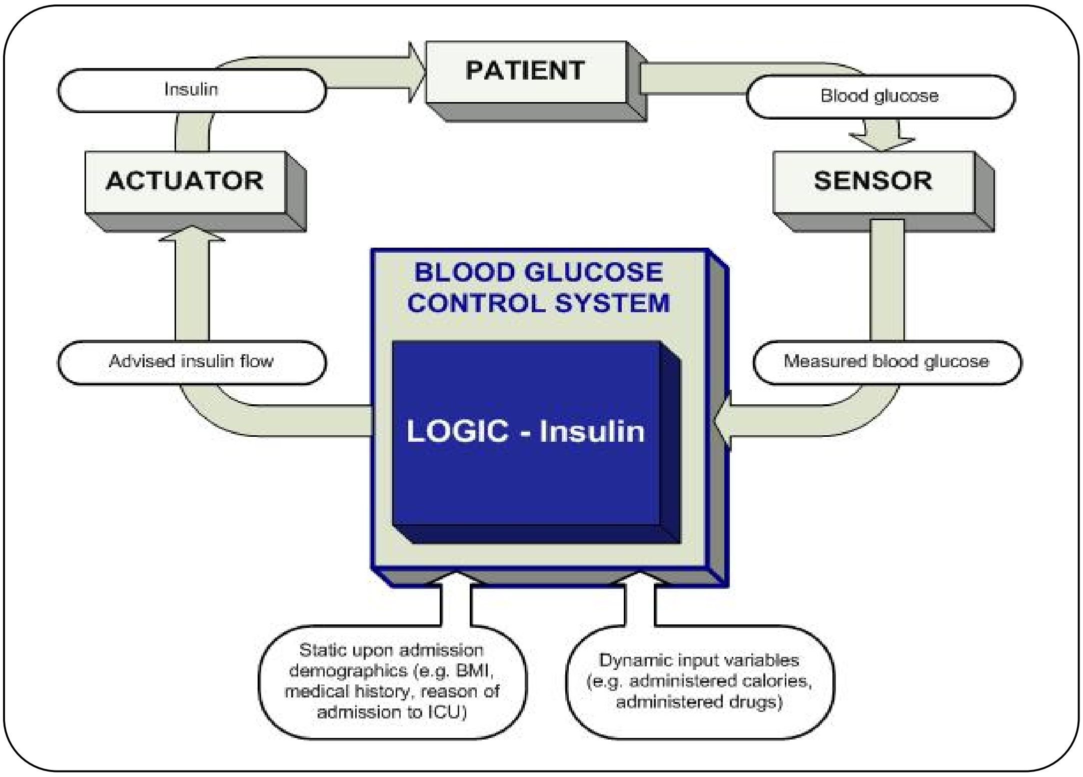

Figure 2. Presentation of the (semi-)automated control system. Undiluted arterial blood glucose is measured every four hours or more frequently in the initial phase or in case of complications. Blood glucose values and other (static and/or dynamic) input variables (i.e. disturbance factors) are denoted as inputs to the control system. In this study the performance of the second version of the LOGIC-Insulin software system will be investigated. This control system determines the insulin rate (or glucose bolus in case of hypoglycaemia) that is required to achieve normoglycaemia. After confirmation by a nurse, this advised insulin flow is delivered to the patient by means of a pump (actuator).

3.4 Identification of any features of design that are different from a previously similar marketed product (if relevant).

The previous version of the LOGIC-Insulin software (evaluated in the LOGIC-1 RCT) was designed for research purpose only and, accordingly, has never been marketed[32]. Adaptations towards the second version, which will be tested and evaluated in the LOGIC-2 RCT that forms the scope of this document, can be summarized as follows:

- Algorithm:
  - Adjustable blood glucose target range
  - Improvements regarding insulin dosing algorithm based on observed data from LOGIC-1 trial and risk management analysis (ISO-norm 14971)
  - Improved blood sampling time algorithm based on observed data from LOGIC-1 trial and feedback nursing staff
- Graphical user interface:
  - Reprogramming Matlab code into Java code incorporating the harmonised standard IEC62304 and the risk management analysis (ISO-norm 14971).
  - Improvements regarding user friendliness (the multi-centre character of present study necessitates a software system that is modern looking and easy to understand and use)
  - Adding unit tests that allow automatic validation
- IT-infrastructure
  - Installation of professional cloud-based IT infrastructure allowing us to easily distribute the LOGIC-Insulin software to the centres participating to this RCT. LOGIC-Insulin is set up as client-server application, in which bedside (thin-client) computers in ICUs access a REST (Representational State Transfer) based HTTP service to do calculations and store data.

3.5 Details of any new or previously untested features of the device

The main part of the LOGIC-Insulin blood glucose algorithm was clinically validated in the LOGIC-1 RCT[32]. Adaptations to both the glucose control as well as the blood sampling time algorithm, the use of a new software environment and the question whether LOGIC-Insulin is also efficient in a *non-Leuven* ICU setting require the clinical evaluation of this second version of LOGIC-Insulin in a large multi-centre RCT.

The optimal insulin dose (or glucose bolus in case of hypoglycaemia) is computed following a robust, biphasic algorithm that includes an adaptive patient model comprising two main phase I variables (patient profile and admission variables) for the initial phase and five main phase II variables (patient profile, blood glucose, insulin dose sequence, nutrition, and steroid medication) for the second phase. The patient profile is defined by the reason for ICU admission, the previous history of diabetes, and the body mass index, whereas the admission variables are set by the severity of illness, the blood

glucose level, and the nutrition, all determined on admission. Further, the model coefficients corresponding to the phase II variables are adapted on the basis of the incoming closed-loop measurements (every sampling episode) and, if appropriate, of an internal glucose control performance evaluation system (every 24 h). This control system assesses the level of blood glucose control (by computing the Glycaemic Penalty Index (GPI)[22], which is a marker of effectiveness of glycaemic control) and the required blood sampling frequency (as a marker of workload for the nurses) observed in the previous 24 h. If appropriate, the ratio between GPI and sampling frequency results in a change of model class and a further adaptation of the model parameters. The LOGIC-Insulin algorithm is schematically summarized in Figure 3.

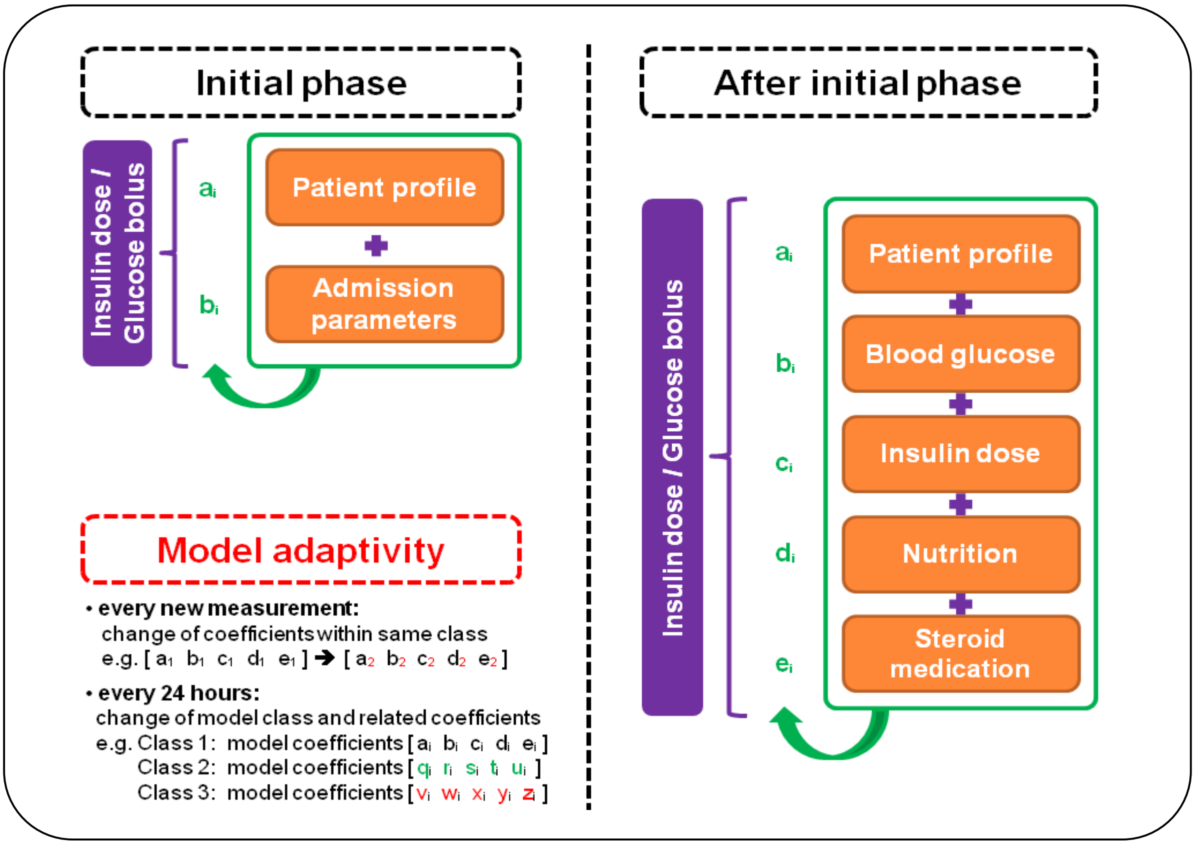

Figure 3. Schematical overview of the LOGIC-Insulin algorithm. The *initial phase* comprises two categories (patient profile and admission parameters) whereas the *following* phases consist of five categories (patient profile, blood glucose, insulin dose, nutrition and steroid drugs). A category (or model) parameter summarizes all patient dynamics related to each corresponding category (e.g. a_i for category 'patient profile'). These parameters vary as a function of patient dynamics. Every 24 hours the glucose profile and workload are evaluated potentially leading to a change of model class.

External ‘disturbance’ factors (such as the change of rate/type of nutrition calories) known for the near future (i.e. the next time horizon varies from 1 to 4 hours) are taken into consideration in the optimization process of the insulin dose sequence. In addition to the original algorithm, the hospital administrator now first selects the preferred blood glucose target range to meet hospital-specific needs and requirements. Therefore, lower and upper limit ranges are 80-100 mg/dL (4.4-5.6 mmol/L) and 110-180 mg/dL (6.1-10.0 mmol/L), respectively. The glucose control and sampling time algorithm are both adapted to the selected hospital-specific target range.

In this multi-centre RCT two different blood glucose target ranges will be selected:

- UZ Leuven and Jessa Hospital Hasselt: 80-110 mg/dL (4.4-6.1 mmol/L),
- AMC Amsterdam: 90-145 mg/dL (5.0-8.0 mmol/L).

Independent of the selected blood glucose target range, the LOGIC-Insulin software approaches glucose control with the following goals:

- **Avoidance of hypoglycaemia and hyperglycaemia:** Observational studies have shown that hyperglycaemia as well as hypoglycaemia are associated, by a J-curved relation, with increased risk of death in critically ill patients[33]. Accordingly, such episodes should be avoided. Severe hypoglycaemia is defined as blood glucose values <40 mg/dL (2.2 mmol/L), whereas common hypoglycaemia is referred to blood glucose values between 40 mg/dL (2.2 mmol/L) and 60 mg/dL (3.3 mmol/L). Analogously, severe hyperglycaemic values are blood glucose values >200 mg/dL (11.1 mmol/L) and common hyperglycaemic values are glycaemia values between 150 mg/dL (8.3 mmol/L) and 200 mg/dL (11.1 mmol/L). These threshold values are only valid for an adult critically ill patient population[23,34]. Different thresholds are used for the paediatric ICU[35], but these patients are not the scope of this study.

- **Avoidance of high glycaemic variability:** Sequential hypoglycaemic and hyperglycaemic events may evoke high glucose variability, due to over- and undershooting the insulin dosage. This is typically the case for simple feedback algorithms (unlike the LOGIC-Insulin algorithm) that do not take into account expected glucose excursions related to known disturbances (e.g. change of rate/type of feeding). Swings in blood glucose levels can be measured by the GPI. It has been shown that a low GPI value was associated with reduced mortality and morbidity for adult critically ill patients[36]. A high glycaemic variability should be avoided, accordingly[36,37].

- **Avoidance of an increased workload:** To ensure the LOGIC-Insulin software can potentially be used in clinical real-life, it is important that the workload for the nurses does not dramatically increase due to the implementation of the software. A typical nurse-patient ratio in Belgian ICUs is 1:2 or even 1:3. In comparison with hospitals world-wide this ratio is already low, indicating the existing high workload for the medical personnel. Therefore, the

algorithm should be able to result in adequate blood glucose control with as few glucose measurements as possible to limit the workload.

The LOGIC-Insulin algorithm proposes an insulin dosage that should be interpreted as an ‘advice’ (i.e. **not** an ‘order’). If the nurse agrees with the proposed insulin flow, he/she effectively adapts (if advised) the rate of the insulin infusion pump. In case the nurse doubts whether the advised insulin dose is adequate (e.g. risk of severe hypoglycaemia), he/she can overrule the insulin administration rate. The reason why must be filled out in the software in case of overruling. This will enable us to improve the algorithm, if appropriate, after the RCT.

When the nurse confirms the insulin dose that will be delivered to the patient, the LOGIC-Insulin software returns the time instant for the next blood sampling. The calculation of this sampling interval depends on the observed and predicted stability of the blood glucose signal, specific events in recent time periods (e.g. hypoglycaemic episode), possibly important (future) changes of the amount of delivered calories, the steroids medication and/or the insulin flow. Further, the LOGIC-Insulin software takes into consideration the clinically recommended, fixed sampling intervals as much as possible (e.g. a blood gas analysis every 4 hours to measure clinical parameters necessary in the treatment of the critically ill patient). The resulting computed sampling interval varies from 1 to 4 hours. If, for example for other reasons than glucose control, the nurse performs a blood gas measurement earlier than expected by the software, he/she is asked to enter this additional blood glucose measurement in LOGIC-Insulin. The additional information allows the software to shift the next blood sampling time and/or the insulin dose.

Though the expected time instants calculated by the software are no hard constraints (i.e. glucose measurement does not have to be sampled *exactly* at the mentioned time instant), an alarm system built in the software warns the nurse if a required glucose measurement has not been sampled yet. A delay from 15 to 30 minutes after the expected sampling time instant may not result in severe blood glucose excursions (though the alarm will be activated), illustrating the robustness of the LOGIC-Insulin algorithm. Analogously, the algorithm does take into account the time instants of previously measured glucose samples. A safety procedure is built in the algorithm to avoid overshooting or undershooting of the insulin dose when e.g. blood glucose values are submitted within a sampling interval of 30 minutes (which is too fast as the minimum sampling time interval is set at 1 hour – except in case of hypoglycaemia danger). Indeed, too short time intervals do not allow to appropriately evaluate the effect of previously administered insulin flows on the blood glucose.

Unlike the previous version of the LOGIC-Insulin software, a client-server application is now set up in which bedside computers in ICUs access a REST based HTTP service to do calculations and store data. The thin client (i.e. the computer available at each ICU bed) typically initiates a request to the server. The latter processes the request and returns the appropriate responses. We will host the LOGIC-Insulin web server in our own cloud environment over HTTPS; the web server itself will

communicate with a cloud hosted database server. In order to auto-fill some existing/available patient data, to detect new measurements and nutrition changes, etc. a LOGIC-Insulin local hospital server will be able to consult the local hospital patient database. As patient data safety and privacy is one of our main concerns, only anonymous data will be stored in our cloud infrastructure. Furthermore, all cloud services will be hosted in a private subnet. Except for a proxy server placed in a DMZ (demilitarized zone), these services are not accessible from the outside. The communication between the proxy server and the private subnet is strictly monitored by a firewall. The proxy server may cache some content and record user activity as well.

The REST architectural style requires neither special software nor hardware to access the application. Encrypted access is built-in in the HTTPS communication (secure access from outside the hospital's IT-network). Different kind of clients (i.e. browsers, stand-alone programs, medical devices) can access the server and the system will work independently of different Windows versions or other operating systems (cross platform).

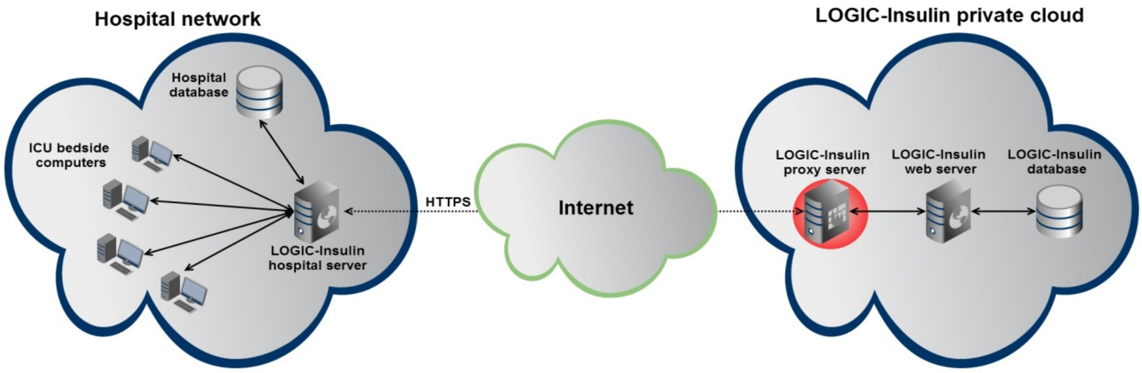

Figure 4. IT-infrastructure to be used in the multi-centre LOGIC-2 RCT. The different components are HTTPS-connected and the LOGIC-Insulin proxy server is the only service which is accessible from the outside.

The LOGIC-Insulin software itself is now coded in an object-oriented code language (Java), meeting European legislation criteria/norms on software development and allowing to generate a modern and easy-to-use graphical user interface. User friendliness is an even more important issue than in the LOGIC-1 RCT due to the multi-centre character of this new RCT. Indeed, the software is designed in such a way that nurses can independently employ LOGIC-Insulin, limiting the guidance by LOGIC-Insulin administrators. However, it is obvious that whenever a usability problem with the software or with the RCT in general arises, the bedside nurse can always contact the 'on duty' LOGIC-Insulin responsible.

Finally, we would like to note that both the re-design of the algorithms and the graphical user interface were developed in close collaboration with the nurses of our Leuven ICU, who have a world-renowned expertise in the field of blood glucose control.

3.6 Clinical data on device in question

The first version of the LOGIC-Insulin software (coded in Matlab) was clinically validated in the LOGIC-1 RCT[32]. The protocol and consent forms were approved by the institutional review board of the University Hospitals Leuven (ML6079) and the Belgian Federal Agency for Medicines and Health Products (80M0437 - *Intensive insulin therapy: nurse control versus advising computer algorithm*). The study had an investigator-initiated, single-centre, prospective, randomised, controlled, parallel-group design. A group of 300 critically ill patients were included and randomised into the control group (glucose regulation by the nurse) or the study group (glucose regulation by LOGIC-Insulin as advising software for the nurse).

LOGIC-Insulin was found to improve the efficacy of tight glycaemic control (TGC) without increasing the rate of hypoglycaemia. The GPI (a marker of efficacy of TGC and the primary outcome measure), was improved in patients treated by the LOGIC-Insulin algorithm. Also other efficacy variables among the time in the target range and the blood glucose variability were improved. Apart from the better glucose control, LOGIC-Insulin was also found to be safer than the expert nurses in terms of avoiding low blood glucose levels. The incidence of hypoglycaemia (<70 mg/dL, <60 mg/dL and <40 mg/dL; (<3.9 mmol/L, <3.3 mmol/L and <2.2 mmol/L) was decreased in the LOGIC-Insulin group. The number of severe hypoglycaemic events (<40 mg/dL; <2.2 mmol/L), often perceived as the major safety indicator, could be reduced to 0 (compared to 6 events for the nurse-controlled group). The better and safer glucose control reached by LOGIC-Insulin came along with an increased workload for the nurse, though. The average sampling interval was decreased from 2.5h to 2.2h. A summary table of the results of the LOGIC-1 RCT are presented in Table 1. The detailed results were published in *LOGIC-Insulin Algorithm-Guided Versus Nurse-Directed Blood Glucose Control During Critical Illness: The LOGIC-1 single-centre, randomised, controlled clinical trial* (Van Herpe T, Mesotten D, Wouters PJ, Herbots J, Voets E, Buyens J, De Moor B, Van den Berghe G. Diabetes Care 2013; 36:188-194).

The new version of the LOGIC-Insulin software system has also been tested in pilot trials in the Leuven ICU in close collaboration with the nurses. During these pilot observations (20 critically ill patients) the nurse obtained an insulin dosage / glucose bolus advice and feedback was asked whether this dosage was acceptable or not (and, in the last case, the reason why). The results of these pilot observations are presented in Table 2. These observed and fully controlled initial tests were used to pre-evaluate the modified glucose control and sampling time algorithms and to get feedback on the new design of the graphical user interface.

		Nurse-driven	LOGIC-Insulin 1	P-value
**Patients**		151	149	
**Study period**	Median (IQR)	1.9 (1.1-3.7) days	1.9 (1.2-4.7) days	0.42
**Blood glucose control**	Mean (SD)<br>Minimum gycaemia<br>Maximum glycaemia	107 (11) mg/dL / 5.9 (0.6) mmol/L<br>28 mg/dL / 1.6 mmol/L<br>328 mg/dL / 18.2 mmol/L	106 (9) mg/dL / 5.9 (0.5) mmol/L<br>45 mg/dL / 2.5 mmol/L<br>272 mg/dL / 15.1 mmol/L	0.36
**GPI**	Median (IQR)	12.4 (8.2-18.5)	9.8 (6.0-14.5)	<0.0001
**Time in Target**	Mean (SD)	60.1 (18.8) %	68.6 (16.7) %	0.00016
**Hypoglycaemia (patients)**	< 70 mg/dL (3.9 mmol/L)<br>< 60 mg/dL (3.3 mmol/L)<br>< 40 mg/dL (2.2 mmol/L)	73 (48.3%)<br>27 (17.9%)<br>5 (3.3%)	48 (32.2%)<br>21 (14.1%)<br>0 (0%)	0.0048<br>0.43<br>0.060
**Hypoglycaemia (samples)**	< 70 mg/dL (3.9 mmol/L)<br>< 60 mg/dL (3.3 mmol/L)<br>< 40 mg/dL (2.2 mmol/L)	170 (3.8%)<br>52 (1.2%)<br>6 (0.1%)	142 (2.3%)<br>39 (0.6%)<br>0 (0%)	<0.0001<br>0.0071<br>0.015
**Sampling interval**	Mean (SD)	2.5 (0.5) hrs	2.2 (0.4) hrs	<0.0001

Table 1. Summary of the results (Intention-to-treat analysis) from the clinical evaluation of the first version of the LOGIC-Insulin software (LOGIC-Insulin 1) as described in *LOGIC-Insulin Algorithm-Guided Versus Nurse-Directed Blood Glucose Control During Critical Illness: The LOGIC-1 single-centre, randomised, controlled clinical trial* (Van Herpe T et al. Diabetes Care. 2013; 36:188-194). (TIT = Time in Target; SD = standard deviation; IQR = Inter Quartile Range)

Blood glucose was tightly controlled. The mean blood glucose was within the target blood glucose range of 80-110 mg/dL (4.4-6.1 mmol/L). The GPI was found to be smaller than 23 indicating effective TGC. This result was also confirmed by the large time into target zone (TIT). Severe hypoglycaemic events (<40 mg/dL; <2.2 mmol/L) could be avoided and the average sampling interval was within the range that the nurses are used to. Mild hypoglycaemic events were still - inevitably - observed. However, provided that hypoglycaemia is diagnosed and treated promptly and adequately, the latter implying prevention of over-correction, the short-term benefit of preventing hyperglycaemia in ICU patients seems to outweigh the short-term risk of hypoglycaemia[26,38].

In general, the results obtained from this pilot patient set (Table 2) are comparable to the results from the LOGIC-1 trial (Table 1). This was expected since the main parts of the glucose control and sampling time algorithms were kept unchanged in the transformation from *Matlab* LOGIC-Insulin to *Java* LOGIC-Insulin. Consequently, this also indicates the software algorithm under study is ready to be tested in the large multi-centre RCT presented in this document.

		**LOGIC-Insulin 2 (Pilot study)**
**Patients**		20
**Pilot study period**	Median (IQR)	2.1 (1.5-5.8) days
**Blood glucose control**	Mean (SD)	104 (9) mg/dL / 5.8 (0.5) mmol/L
	Minimum glycaemia	47 mg/dL / 2.6 mmol/L
	Maximum glycaemia	188 mg/dL / 10.4 mmol/L
**GPI**	Median (IQR)	10.3 (7.2-13.7)
**Time in Target**	Mean (SD)	58.6 (16.0) %
**Hypoglycaemia (patients)**	< 70 mg/dL (3.9 mmol/L)	4 (20%)
	< 60 mg/dL (3.3 mmol/L)	3 (15%)
	< 40 mg/dL (2.2 mmol/L)	0 (0%)
**Hypoglycaemia (samples)**	< 70 mg/dL (3.9 mmol/L)	12 (1.5%)
	< 60 mg/dL (3.3 mmol/L)	7 (0.8%)
	< 40 mg/dL (2.2 mmol/L)	0 (0%)
**Sampling interval**	Mean (SD)	2.4 (0.1) hrs

Table 2. Overview pilot tests with the second version of the LOGIC-Insulin software (LOGIC-Insulin 2) in Leuven ICU as preparation for the LOGIC-2 multi-centre RCT.

3.7 Benefit/Risk analysis

The risk of hypoglycaemia is unfortunately not avoidable if a narrow blood glucose range is targeted, independent of the type of controller (nurse-driven or computer-driven). As already stated above, however, the short-term benefit of preventing hyperglycaemia in ICU patients seems to outweigh the short-term risk of hypoglycaemia[26,38]. It is of course important that hypoglycaemic events are diagnosed as soon as possible and that they are treated promptly and adequately. Over-correcting by drastically reducing the insulin dose and/or administering an overload of glucose calories should be avoided as such swings from hypoglycaemia to hyperglycaemia typically lead to a high glucose variability. The latter is associated with higher mortality and morbidity in critically ill patients[37].

Therefore, the LOGIC-insulin software advises the nurse on the bolus of glucose calories (besides the advice on reduction of the insulin dose) in case of observed or predicted hypoglycaemic episodes. Another action that has been taken to minimise the potential risk of hypoglycaemia is the introduction of an alarm system that is activated when no glucose measurement is entered in the LOGIC-Insulin software after the expected sampling time instant.

The risk management procedure describing all other risks, their severity, probability of appearance, probability of detection and the measure to control each hazard is added in **Appendix 3**.

3.8 Summary and analysis of pre-clinical testing

The pre-clinical test phase of the new version of the LOGIC-Insulin software comprised two major steps:

1. *Adapted glucose control and sampling time algorithms*
   The extended algorithms were critically evaluated by simulating the advised insulin dosages, glucose boluses, and sampling time instants using the patient data observed in the LOGIC-1 RCT (LOGIC-arm, 149 critically ill patients). Deviations from the original advices (i.e. during the study) were qualitatively and quantitatively analysed in an iterative process.

2. *Java graphical user interface*
   The new graphical user interface (coded in Java) was developed following the harmonised standard IEC62304. It was extensively tested preceding any clinical (pilot) test. Unit tests were added to allow automatic validation and extensive test scenarios were described, applied and reported in an issue tracking database. Further, a risk management analysis, as discussed in ISO-norm 14971, describing all potential hazards, assessing their severity, probability of appearance, probability of detection and the measure to control each hazard is executed. The LOGIC-Insulin risk management procedure is added in **Appendix 3**.

3.9 Description of materials coming into contact with the body

The medical device under study is software and can be installed on a computer or a thin client. There are no materials that come into contact with the body. This statement is also added in **Appendix 4**.

3.10 Description of biocompatibility and biological safety

Since there is no contact between the body and the medical device under study, issues concerning biocompatibility and biological safety are not present.

However, there is the potential risk that the LOGIC-Insulin algorithm proposes incorrect insulin dosages. Delivery of too much insulin may provoke hypoglycaemic events, whereas the administration of too few insulin may lead to hyperglycaemia.

Therefore, the safety of the patient can be guaranteed in the following way:

- **Advising system:** The LOGIC-Insulin software only gives an *advice* to the nurse. The bedside nurses apply glucose control in daily ICU practice. They are experts in assessing the glycaemic response (e.g. after administering insulin) and can simply overrule the software in case of expected hypoglycaemic or hyperglycaemic episodes. In case of overruling, the nurse is asked to fill out the motivation for the overruling in the software. During the LOGIC-1 RCT the LOGIC-Insulin advices were overruled only in a minority of the cases[32]. In 21 patients, nurses did a major (≥ 1 IU/h) overruling of the software (i.e. 0.46% of blood glucose measurements). Of these 25 major overrules, only 1 was justified to avoid hypoglycaemia. The other overrules were explained by a clinical context unknown to the software (e.g. inadvertent change of nutrition without informing the software or a disconnected insulin infusion line). The glucose control algorithm has now been modified to avoid the justified major overrule in future similar scenarios.

- **Semi-automated closed-loop:** The proposed medical device does not act as fully-automated closed-loop software. Besides the need for official confirmation by the nurse (see above), the insulin infusion pump needs to be adapted (if appropriate) manually by the nurse. This is a second safety procedure as the software is not able to automatically steer the insulin infusion pump.

- **Alarm system:** An alarm system to warn the nurse when a following blood sampling is expected. Though the next glucose measurement should not be sampled exactly at the suggested time instant to guarantee a well-controlled blood glucose signal, the alarm signal will be activated if no new glucose measurement is submitted within the expected sampling interval.

3.11 Identification of any pharmacological components of device

The medical device under study does not contain any pharmacological component. We want to emphasize that the LOGIC-Insulin algorithm does not act as a closed-loop system. Only advice on the insulin dosage (or glucose bolus in case of hypoglycaemic events) is given. For safety reasons, this advice needs to be confirmed by the expert nurses before the insulin flow is effectively delivered to the critically ill patient.

3.12 Design drawings

Figure 5 presents a general overview on the integration of the developed LOGIC-Insulin software with respect to the critically ill patient and the nurse who is treating this patient. Blood glucose is measured preferably at the time instant that is proposed by the LOGIC-Insulin software. Besides the blood glucose measurement, also other input factors (such as administered nutrition calories, medication, history of diabetes, etc.) are submitted to the LOGIC-Insulin algorithm. Next, an "optimal" insulin dose advice and an advice on the time instant for the next blood sample are calculated. The expert nurse can overrule both advices if appropriate. Furthermore, the nurse manually adapts the flow of the insulin infusion pump (if needed) and the next blood sample is taken at the appropriate time instant.

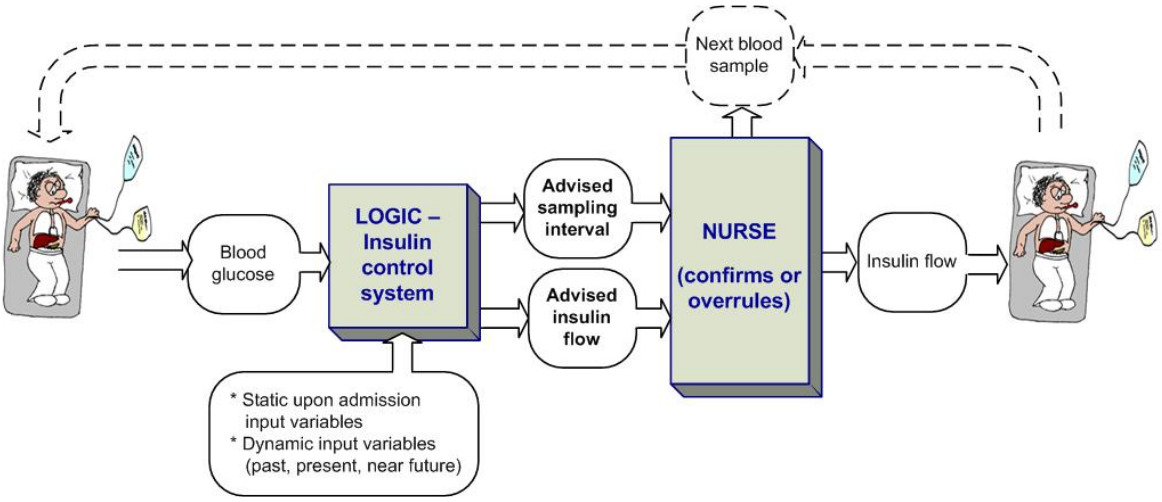

Figure 5. General overview on the integration of the developed LOGIC-Insulin software

3.13 Description of software

The LOGIC-Insulin software comprises an insulin dose calculator for adult critically ill patients integrated in a professional, easy-to-use graphical user interface coded in Java. During the fine-tuning process of the included algorithms and the design of the new Java software environment we gathered feedback from the nursing and medical staff on a regular base allowing to iteratively adapt the software package towards the needs present in daily-life ICU practice.

LOGIC-Insulin uses a set of input variables to optimise the insulin dose sequence and the blood sampling time instants. The most important variable is the blood glucose, which is, like in the LOGIC-1 RCT, intermittently measured using a blood gas analyser (sampling interval varying from 1-4 hours mainly depending on the stability of the blood glucose signal and expected potential effects of disturbance factors). Currently no near-continuous glucose sensors are available to measure glycaemia accurately in an ICU-setting.

A second important variable is the type and amount of nutrition that is administered to the patient. All nutrition types available in the ICUs that are participating to this multi-centre RCT, and their composition have been submitted to the LOGIC-Insulin nutrition database. If, during the study, the nutrition policy in an ICU would change, modifications or new entries to this database can be easily introduced by a LOGIC-Insulin administrator. The modern looking Java graphical user interface allows the nurse to simply add/delete/modify the nutrition type and rate, from which the number of glucose/carbohydrates are automatically computed. Figure 6 presents a screen shot of the nutrition selection.

Other input variables comprise the steroid drugs and non-changing (static) variables such as body height and mass, history of diabetes, reason of ICU-admission, etc. Finally, insulin is administered via an accurate syringe infusion pump.

While awaiting the next blood sample, the LOGIC-Insulin 'waiting screen' shows the last measured blood glucose, the current flow of insulin/nutrition and the evolution over time (graph and table format) for the blood glucose and the relevant input variables (e.g. nutrition). The new software further allows to select the time zone of the data that is of interest for the user (varying from full overview of all data to a very specific time slot). Accordingly, trend analysis of the data and verification of the insulin infusion pump settings are facilitated using the LOGIC-Insulin software. This undoubtedly further enhances patient safety.

Finally, a detailed manual of the LOGIC-Insulin software is added in **Appendix 5**. Since Dutch is the main speaking language in all hospitals participating to this multi-centre RCT, the software and the manual are written in Dutch.

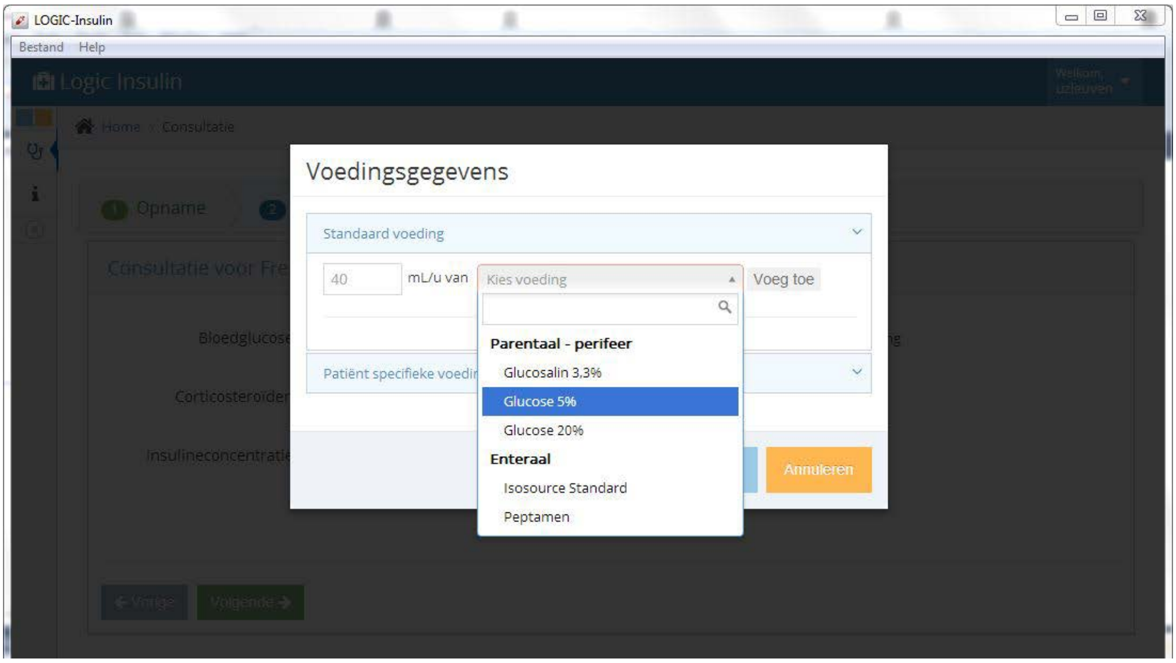

Figure 6. Nutrition selection screen shot of LOGIC-Insulin. Type and rate of nutrition can be easily selected. The number of glucose/carbohydrate calories are computed automatically.

3.14 Method of sterilisation, validation, cleaning, disinfection, …

LOGIC-Insulin is standalone medical software installed on a bedside computer or a thin client computer connected to a local hospital server or to the LOGIC-Insulin cloud environment. This last option has been chosen for the multi-centre RCT presented here. Important to note is that there is never any physical contact between the critically ill patient and the device under study. Therefore, the aspects mentioned in this paragraph are not relevant.

3.15 Identification of any tissues of animal origin

The aspects mentioned in this section are not relevant for the device under study. This statement is also added in **Appendix 6**.

3.16 Identification of any special manufacturing conditions required

The aspects mentioned in this section are not relevant for the device under study.

3.17 List of relevant Standards

The Java version of LOGIC-Insulin was developed according the norms and guidelines as described in the harmonised standard IEC 62304 and the technical report IEC/TR80002-1. The ISO-norm 14971 was used as basis for the risk management analysis. Further, based on the medical device directives 93/42/EEC and 2.4/1 rev.9, the LOGIC-Insulin software has been classified as class IIb device. Finally, the clinical investigation of this multi-centre RCT meets the criteria as presented in the WMA Declaration of Helsinki and the Good Clinical Practice norm.

Though not limited to the list below, most important relevant standards used in the design of this medical device, the corresponding risk analysis and the proposed multi-centre RCT are:

- WMA Declaration of Helsinki - Ethical Principles for Medical Research Involving Human Subjects, amended version of 59th WMA General Assembly, Seoul, October 2008,
- International Conference on Harmonisation (ICH), including Good Clinical Practice (GCP) E6 and Clinical Trials E8-10,
- MEDDEV 2.7.2 The European Commission Guideline on Medical Devices
- MEDDEV 2.4/4 Guidelines on clinical investigation: a guide for manufacturers and notified bodies
- MEDDEV 93/42/EEC Counsil Directive 93/42/EEC concerning medical devices
- MEDDEV 2.4/1 rev.9 Guidance document on classification of medical devices
- ISO 14155 Clinical investigation of medical devices for human subjects.
- ISO 14971 Medical devices — Application of risk management to medical devices
- ISO 13485 Medical devices — Quality management systems — Requirements for regulatory purposes
- ISO/TR 14969 Medical devices - Quality management systems - Guidance on the application of ISO 13485:2003
- IEC 62304 Medical device software – Software life cycle processes
- IEC/TR80002-1 Medical device software – Part 1: Guidance on the application of ISO 14971 to medical device software
- NB-MED/2.5.1/Rec5 Guidance document on technical documentation

3.18 Instructions for use including risks, contra indications and warnings

The medical and nursing staff of the trial centres will be intensively trained (2 hours general introduction for each shift and at the bedside for the first two patients for each nurse) by the clinical investigators or their delegates.

Specific attention will be paid to the risks in the study, notably hypoglycaemia or refractory hyperglycaemia. As included in the protocol, in the case of recurrent hypoglycaemic episodes or hyperglycaemia that cannot be controlled under the LOGIC-Insulin software or any change in condition compromising the safety of the patient, the patient can be withdrawn from the study. The investigators have to be notified as soon as possible by the treating nurse or physician.

Contra-indications for the study are as follows:

- Absence of one of the components of the TGC strategy: arterial line for blood sampling, accurate syringe pump for the administration of insulin, blood gas analyser for blood glucose measurements.
- The patient is eating.

As these contra-indications are already standard practices for TGC in the participating ICUs, the nursing and medical staff does not need any specific instructions in this regard.

Specific warnings regarding the risks, notably hypoglycaemia and refractory hyperglycaemia, are not necessary as the study will only be executed by nurses with expertise in TGC. Trainee nurses and recently appointed nurses will not be allowed to treat patients independently under the study protocol.

3.19 Provisions for the recovering of the device and prevention of unauthorised use

The LOGIC-Insulin software is meant for normalising the blood glucose levels in all adult critically ill patients and can therefore be simply 're-used' by submitting a unique patient number to the software. Each new patient ID number activates the software to start a new session as can be observed in the manual, see also **Appendix 5**.

4. CLINICAL INVESTIGATION PLAN: General information

4.1 Investigator team

The investigator team of this multicentre clinical investigation comprises the following researchers:

- **Coordinating principle clinical investigator:**

Prof. Dr. Dieter Mesotten, MD, PhD
Katholieke Universiteit Leuven
Dept. Intensive Care Medicine - University Hospitals Leuven
Herestraat 49
B-3000 Leuven
Belgium
dieter.mesotten@med.kuleuven.be
Tel: +32 16 344021
Fax: +32 16 344015

As a qualified anaesthetist and intensive care clinician Prof. D. Mesotten is since August 2008 staff specialist in the Department of Intensive Care Medicine of the KU Leuven University Hospitals in Belgium. Endocrine changes and liver dysfunction during critical illness are his clinical expertise and research focus. Prof. Mesotten obtained his PhD in 2003, dealing with the impact of tight blood glucose control through intensive insulin therapy on metabolism. During his PhD he worked in the Kolling Institute of Medical Research, University of Sydney. He currently runs a young research unit of one post-doctoral and three doctoral researchers and has extensive experience in molecular biology. Recently his research diversified to the clinical development of an algorithm to control blood glucose levels, as well as the long-term neurocognitive outcome of critically ill children in the context of tight glycaemic control. To date Prof. D. Mesotten has published 40 peer-reviewed manuscripts. Prof. D. Mesotten has given more than 30 invited presentations at national and international meetings and has received several research awards.

- **Principal clinical investigators KU Leuven**

Prof. Dr. Greet Van den Berghe, MD, PhD
Katholieke Universiteit Leuven
Dept. Intensive Care Medicine - University Hospitals Leuven
Herestraat 49
B-3000 Leuven
Belgium
greet.vandenberghe@med.kuleuven.be

Greet Van den Berghe graduated “summa cum laude” at the faculty of Medicine of the KU Leuven, Belgium, in 1985 after which she specialized in Anaesthesiology and later in Intensive Care Medicine. She studied biostatistics and in 1994 completed a PhD thesis on Endocrinology of critical illness. From 1995 until 2004, she was a Clinical Research Investigator for the Fund for Scientific Research Flanders, Belgium. She was appointed head of the Department of Intensive Care Medicine at the University Hospitals Leuven in 2002. This intensive care unit is a large (56 beds) tertiary centre treating around 2500 patients per year, adults as well as children. Greet Van den Berghe is Professor of Medicine at the Leuven University, and has been running an active research program on the endocrinology of critical illness since 1995. She is also a member of the Belgian Royal Academy of Medicine. Greet Van den Berghe published over 350 papers in peer-reviewed journals, and has authored several chapters in international textbooks on intensive care and endocrinology. She has been an invited speaker at many scientific meetings worldwide.

- **Clinical co-investigator KU Leuven**

Prof. Dr. Alexander Wilmer, MD, PhD
Katholieke Universiteit Leuven
Medical Intensive Care Unit - University Hospitals Leuven
Herestraat 49
B-3000 Leuven
Belgium
alexander.wilmer@med.kuleuven.be

Alexander Wilmer obtained his medical degree from the Ludwig-Maximilians University of Munich in 1989 and his doctorate in 1990 (magna cum laude). He specialized in Internal Medicine and Intensive Care in Munich, Houston and Leuven. He is currently Associate Clinical Professor at the Faculty of Medicine of the Catholic University of Leuven and Head of Clinic of the Medical Intensive Care Unit of the University Hospitals Leuven. His clinical

research is focused mainly on clinical studies of the management of liver failure in the ICU and nutritional support strategies in the critically ill. He is author or co-author of numerous articles in medical journals, as well as reviews and book chapters.

- **Principal clinical investigator Jessa Hospital Hasselt**

Dr. Jasperina Dubois, MD
Dept. Anaesthesia and Intensive Care – Jessa Hospital
Stadsomvaart 11
B-3500 Hasselt
Belgium
jasperina.dubois@jessazh.be

Jasperina Dubois studied Medicine at the KU Leuven, where she graduated “magna cum laude” in 2000. In 2005 she completed her internship in Anaesthesiology at the University Hospitals Leuven, followed in 2006 by a fellowship in Intensive Care Medicine at the same institution. Thereafter, Dr. Dubois started working in the department of Anaesthesia and Intensive Care of the Jessa Hospital Hasselt. In this large non-academic centre, she works in a mixed medical–surgical 36-bed intensive care unit. She collaborated in a large multicentre trial conducted by the research team of Prof. Dr. Greet Van den Berghe as a local principal investigator. Dr. Dubois is also a member of the research group of the department of Cardiac Surgery - Cardiac Anaesthesia – Experimental Hematology of the Jessa Hospital Hasselt, which focuses on animal studies on cardiac stem cells for myocardial repair.

- **Clinical co-investigator Jessa Hospital Hasselt**

Dr. Aimé Van Assche, MD
Dept. Anaesthesia and Intensive Care – Jessa Hospital
Stadsomvaart 11
B-3500 Hasselt
Belgium
aime.vanassche@jessazh.be

After graduating from medical school at the KU Leuven in 1982, Dr. Aimé Van Assche specialised in Anesthesiology and Intensive Care Medicine at the AZ St-Jan Brugge and the Universität Ulm. In 1986 he became a staff member of the department of Anesthesiology and Intensive Care of the Virga Jesse Hospital Hasselt, where he was appointed head of the department of Intensive Care in 1991. Since 1997, when Dr. Van Assche became a qualified trainer in Intensive Care Medicine, the department has run a clinical fellowship programme in Adult Intensive Care Medicine. Currently, the mixed medical-surgical ICU has 36 beds.

- **Principal clinical investigator AMC, Amsterdam**

Prof. Dr. Marcus Schultz, MD, PhD
Dept Intensive Care Medicine
Academic Medical Center
Meibergdreef 9
1105 AZ Amsterdam
The Netherlands
marcus.j.schultz@gmail.com

Marcus J. Schultz completed his medical degree (cum laude) and residency in internal medicine at the Academic Medical Center at the University of Amsterdam, Amsterdam, The Netherlands. He obtained his doctorate from the University of Amsterdam. Dr. Schultz is currently professor of intensive care medicine at the Academic Medical Center. He serves as one of the principal investigators of the Academic Medical Center and chairs the Laboratory of Experimental Intensive Care and Anaesthesiology. Dr. Schultz has published over 350 articles in international journals and over 60 chapters in scientific books, and has received several research awards. His main research interests are in the area of acute lung injury and pneumonia, and mechanical ventilation, specifically preclinical and clinical studies on the interaction between infection/inflammation and coagulation, and new anticoagulants. In addition, Dr. Schultz initiated several studies focusing on mechanical ventilation settings during general anesthesia for surgery. His second interest involves glucose control and monitoring in the intensive care unit setting. Finally, he performs implementation studies in western countries (The Netherlands) and low– (Bangladesh, India and Nepal) and middle–income countries (Sri Lanka, Malaysia and Brazil).

- **Clinical co-investigator AMC, Amsterdam**

Dr. Roosmarijn van Hooijdonk, MD
Dept Intensive Care Medicine
Academic Medical Center
Meibergdreef 9
1105 AZ Amsterdam
The Netherlands
r.t.vanhooijdonk@amc.uva.nl

Roosmarijn T.M. van Hooijdonk completed her master of Science in Medicine at the University of Groningen. During her study she became interested in the field of glucose control in the Intensive Care. This led to her current position as a PhD student within the

department of intensive care medicine, at the Academic Medical of the University of Amsterdam, Amsterdam, the Netherlands. Her main topic of interest is glucose monitoring in intensive care setting. Additional research activity involves a large national study into sepsis diagnostics and prognostics.

- **Engineering investigators KU Leuven**

Prof. Dr. ir. Bart De Moor, PhD
Katholieke Universiteit Leuven
Dept. Electrical Engineering Department (ESAT-STADIUS)
Kasteelpark Arenberg 10
B-3001 Heverlee
Belgium
bart.demoor@esat.kuleuven.be

Bart De Moor is a full professor at the Department of Electrical Engineering of the KU Leuven in the research group STADIUS. His research interests are in numerical linear algebra, algebraic geometry and optimization, system theory and system identification, quantum information theory, control theory, data-mining, information retrieval and bio-informatics. Currently, he is leading a research group of 10 PhD students and 4 postdocs and in the recent past, 80 PhDs were obtained under his guidance. He has been teaching at several universities in Europe and the US. He is also a member of several scientific and professional organizations and jury member of several awards (Tech Art Prize, Innovation Award of the Flemish Government, Barco Innovation Award, ICOS Award, Egemin Award). His work has won him several scientific awards (Leybold-Heraeus Prize (1986), Leslie Fox Prize (1989), Guillemin-Cauer best paper Award of the IEEE Transactions on Circuits and Systems (1990), Laureate of the Belgian Royal Academy of Sciences (1992), bi-annual Siemens Award (1994), best paper award of Automatica (IFAC, 1996), IEEE Signal Processing Society Best Paper Award (1999)). In November 2010, he received the 5-annual FWO Excellence award. From 1991-1999 he was the chief advisor on Science and Technology of several ministers of the Belgian Federal Government (Demeester, Martens) and the Flanders Regional Governments (Demeester, Van den Brande). From December 2005 to July 2007, he was the Head of Cabinet on socio-economic policy of the minister-president of Flanders, Yves Leterme, capacity in which he was the coordinator of a new socio-economic business plan for the Flemish region. He was and/or is in the board of 6 spin-off companies, of the Flemish Interuniversity Institute for Biotechnology (VIB), the Institute for Broad Band Technology (iMinds), the Study Center fro Nuclear Energy, the Flemish Children Center Technopolis, the Alamire foundation, the Belgian AstraZeneca foundation and other scientific and cultural organizations. He is also the Chairman of the Industrial Research Fund, Hercules (heavy

equipment funding in Flanders), and member of the Board of the Danish National Research foundation. He is a co-founder of and in the Board of the International School of Leuven.

Dr. ing. Tom Van Herpe, PhD
Katholieke Universiteit Leuven
Dept. Intensive Care Medicine - University Hospitals Leuven
Herestraat 49
B-3000 Leuven
Belgium
tom.vanherpe@esat.kuleuven.be

Tom Van Herpe obtained his PhD degree at the KU Leuven, Belgium, in 2008. In his research he designed a first control system for normalizing the blood glucose in critically ill patients and new procedures to assess glucose sensors and glucose control. Currently he is a Postdoctoral Researcher with the Intensive Care Unit of the Medical department and the STADIUS Research Division of the Electrical Engineering department (ESAT) of the KU Leuven. His expertise and research focus are medical devices, in particular for the Intensive Care Unit and the Diabetes area: blood glucose sensor technology, artificial pancreas, blood glucose control, biomedical modeling and control, statistical evaluation of glucose sensors, etc. In 2005, he won the Diabetes Technology Peterson Student Research Bronze Price during the Fifth Annual Diabetes Technology Meeting in San Francisco. In 2012, he won the KU Leuven Da Vinci price for the creation and valorisation of his research.

Guy Veraghtert, MSc
Katholieke Universiteit Leuven
Dept. Intensive Care Medicine - University Hospitals Leuven
Herestraat 49
B-3000 Leuven
Belgium
Guy.Veraghtert@esat.kuleuven.be

Guy Veraghtert studied Master of Science (Informatics) at the KU Leuven, Belgium from 2000 to 2004. Afterwards he worked nearly 8 years as a Java-consultant at ACA-IT Solutions. In this role he worked in various environments: ranging from the Telecom sector through the banking sector to government institutions. He has built a strong background in software architecture, designing & implementing enterprise class systems and B2B integration. Instructing Java, contributing to open source projects and speaking at conferences is another way of his involvement with the technology. In 2011 he won the JavaOne Rockstar award for

his outstanding session content and speaking ability. As member of the STADIUS Research Division of the Electrical Engineering department (ESAT) of the KU Leuven his current interests lie in the design & development of medical software.

4.2-4.3 Institution, Sponsor, Manufacturer

The clinical investigation will be conducted in:

- **KU Leuven - University Hospitals Leuven (UZ Leuven)**
  Dept. Intensive Care Medicine
  Herestraat 49
  B-3000 Leuven
  Belgium

- **Jessa Hospital Hasselt**
  Dept. Anaesthesia and Intensive Care
  Stadsomvaart 11
  B-3500 Hasselt
  Belgium

- **Academic Medical Center (AMC)**
  Dept Intensive Care Medicine
  Academic Medical Center
  Meibergdreef 9
  1105 AZ Amsterdam
  The Netherlands

The sponsor of this study and the Manufacturer are the KU Leuven:

- **KU Leuven**
  Leuven Research & Development
  Waaistraat 6
  B-3000 Leuven
  Belgium

4.4 Synopsis of the clinical investigation plan

4.4.1 Clinical background

While stress hyperglycaemia has traditionally been regarded as an adaptive, beneficial response, it is clear from observational studies that hyperglycaemia, as well as hypoglycaemia, are associated with increased risk of death in critically ill patients. The association between blood glucose levels and mortality risk follows a J-curved relationship with the nadir roughly between 80-140 mg/dL (4.4-7.8 mmol/L)[1,3,33]. However, in order to show a causal relationship between hyperglycaemia and mortality risk, RCTs that target and achieve different blood glucose levels are required.

The first RCT that lowered blood glucose levels by insulin therapy during critical illness was performed in Leuven[4]. As one would expect from a seminal study, it had a proof-of-concept design: single centre, a homogeneous patient population (mainly cardiac surgery and high risk/complicated non-cardiac surgery), standardised arterial blood glucose measurements and central venous continuous insulin infusion using an accurate syringe pump. The study targeted a “strictly normal level for fasting blood glucose”, i.e. 80-110 mg/dL (4.4-6.1 mmol/L) versus treating hyperglycaemia only when it exceeded the renal threshold of 215 mg/dL (12.0 mmol/L). The insulin dose-adaptations were based on a guideline to stimulate intuitive and anticipating decision making by bedside nurses. Extensive training sessions were organised to optimise the execution of tight glycaemic control. The study showed that maintaining strict normoglycaemia by “intensive insulin therapy” lowered ICU mortality from 8.0% to 4.6% (absolute risk reduction (ARR) 3.4%) and in-hospital mortality from 10.9% to 7.2% (ARR 3.7%). It also reduced morbidity by preventing organ failure. This was reflected in a shorter duration of mechanical ventilation, a decreased incidence of acute kidney failure, severe infections and polyneuropathy and less blood transfusions.

Generalising to the medical[5] and paediatric[6] critically ill patient population, these results were confirmed in the same context of a well-controlled single centre expert setting and in implementation studies[7-11]. In contrast, large multi-centre studies (VISEP, GLUCONTROL and NICE-SUGAR) could not confirm this survival benefit[12-14].

Besides the differences in the target level for blood glucose in the control groups (much lower for GLUCONTROL and NICE-SUGAR than in the Leuven trials), methodological disparity in the execution of the complex intervention of tight glycaemic control may have contributed significantly to the contradicting results[16,39]. Firstly, allowing inaccurate blood glucose measurement devices, in combination with different blood sampling sites and types of infusion pumps, may have led to unnoticed swings in blood glucose levels. Secondly, the level of expertise of the intensive care nurses with the therapy may have been variable due to low study patient numbers per centre. Hence, these multi-centre trials failed to reach the predefined blood glucose targets in the intervention group, resulting in a significant overlap with the control group.

Now everyone acknowledges that the implementation and evaluation of complex interventions is a stepwise, gradual process that consumes vast amounts of time and resources[17]. And clearly in the case of tight glycaemic control the fringe conditions were overlooked, when this complex intervention made the step from proof-of-concept study to large multi-centre, effectiveness trials. The scientific community has hinted that the focus of future studies should shift to the accuracy of blood glucose measurements[18], glycaemic management protocols[19] and the validation of markers of quality of glycaemic control[20-23].

Besides the issue of efficacy in steering blood glucose levels in the predefined target range, strategies to avoid hypoglycaemia, inherently associated with tight glycaemic control, are a burning question[24]. The incidence of severe hypoglycaemia < 40 mg/dL (2.2 mmol/L) increased in all studies 5-13 fold[16]. Even in an experienced centre the incidence of hypoglycaemia rose to 11.3% in the adult ICU population[25] and 25% in the paediatric critically ill patients[6]. Severe or prolonged hypoglycaemia can cause convulsions, coma, and irreversible brain damage as well as cardiac arrhythmias[34]. The risk of hypoglycaemia is a concern when intensive insulin therapy is implemented in the ICU because early hypoglycaemic symptoms are not easily recognised in sedated critically ill patients. However, provided that hypoglycaemia is diagnosed and treated promptly and adequately, the latter implying prevention of over-correction, the benefit of preventing hyperglycaemia in ICU patients seems to outweigh the short-term risk of hypoglycaemia[26,38,40]. In the studies with less attention to the methodological aspects and implementation process of tight glycaemic control the association between hypoglycaemia and poor outcome is more pronounced[27,28].

Computerised algorithms, such as the LOGIC (Lova Glucose control for Intensive Care)-Insulin algorithm, may improve glycaemic control and lower hypoglycaemic episodes[29,30]. Computer-assisted blood glucose control software may in the future be combined with accurate, continuous blood glucose monitoring devices, preferably in closed-loop systems to avoid hypoglycaemia and large blood glucose swings[31].

4.4.2 Aim of the study

The primary objective of the study is to demonstrate the superiority of the computer algorithm (LOGIC-Insulin) against the standard routine protocol for the normalisation of blood glucose concentrations in the ICU.

The secondary objective of the study is to demonstrate cost-effectiveness of the implementation of the LOGIC-Insulin algorithm.

4.4.3 The LOGIC-Insulin control software

The developed LOGIC-Insulin software comprises advanced algorithms to normalise the blood glucose of critically ill patients and to define the next blood sampling time interval. At these time instants (or earlier in case the nurse requires a blood sample for reasons other than glucose control) or when important changes regarding type/amount of nutrition calories are observed, the LOGIC-

Insulin algorithm computes the most optimal insulin dose. If (potentially dangerous) hypoglycaemic episodes are monitored or expected, an advice on glucose bolus calories is given (insulin flow reduced to 0 IU/h). The algorithm aims at reaching normoglycaemia as quickly as possible, avoiding hypo- and hyperglycaemic episodes and avoiding high blood glucose variability (i.e. blood glucose swings). The suggested insulin dose needs to be confirmed by the nurse before effectively delivering to the patient. Based on his/her expertise, the nurse evaluates the suggested insulin dose and overrules it if appropriate.

Further, the LOGIC-Insulin algorithm advises the nurse on the time instant when – approximately – the next blood glucose measurement is expected. The calculation of this sampling interval depends on the observed and predicted stability of the blood glucose signal, specific events in recent time periods (e.g. hypoglycaemic episode), possibly important (future) changes of the number of administered calories, the corticosteroids and/or the insulin flow. The resulting computed sampling interval varies from 1 to 4 hours.

Finally, a professional and user-friendly graphical user interface meeting European norms/criteria on software development was developed for the scope of CE-approval for this medical device after the multi-centre RCT.

4.4.4 Risks

Hypoglycaemia is an important and unavoidable risk associated with the normalisation of the blood glucose in a narrow target range. Therefore, hypoglycaemic events should be diagnosed and treated promptly and adequately. Over-correcting by drastically reducing the insulin dose and/or administering an overload of glucose calories should be avoided as such swings from hypoglycaemia to hyperglycaemia typically lead to a high glucose variability. The latter is associated with higher mortality and morbidity in critically ill patients[36,37]. The LOGIC-insulin software advises the nurse on the bolus of glucose calories (besides the advice on reduction of the insulin dose) in case of observed or predicted hypoglycaemic episodes. Another action that has been taken to minimise the potential risk of hypoglycaemia is the introduction of an alarm system that is activated when the waiting period for the next blood sample has passed (i.e. longer than expected sampling interval).

4.4.5 Clinical study

This is an international, pragmatic effectiveness multi-centre interventional RCT. In accordance with state-of-the-art trial design it will consist of a screening, an intervention and a predefined follow-up assessment. The study will be performed in three hospitals. The University Hospitals Leuven, Leuven, Belgium has five ICUs. The Jessa Hospital, Hasselt, Belgium has two ICUs and the Academic Medical Center at the University of Amsterdam, has one ICU. Hence, the study will take place at eight participating ICUs.

The study has been conceived as a superiority trial for improving the effectiveness of blood glucose control, measured by the GPI (primary outcome measure) and the time-in-target. The LOGIC-1 single centre study[32] showed that, compared to standard care, the LOGIC-Insulin computer algorithm decreases the GPI from median 12.4 (IQR 8.2-18.5) to 9.8 (IQR 6.0-14.5), hence an observed difference of 2.6. A retrospective analysis of 461 patients from the AMC, Amsterdam showed a GPI of median 13 (IQR 6-24) and of 739 patients from the Jessa Hospital Hasselt revealed a GPI of 24 (IQR 17-33). A study, in which all centres would include a similar number of patients, with a confidence level of 5% (alpha error) and a statistical power of 90% (beta error level 10%) will require 458 patients in each arm of the study to decrease GPI from mean 21 to 19 (sigma 14) in a two-sided test. For an increase of the time-in-target from 45% to 50% with a sigma of 29%, a study with a two-sided confidence level of 5% (alpha error) and a statistical power of 90% (beta error level 10%) will require 707 patients in each arm.

The study will also be powered to detect differences in the incidence of mild hypoglycaemia (<70 mg/dL or < 3.9 mmol/L), a safety outcome variable. In the LOGIC-1 RCT the use of the LOGIC-Insulin algorithm decreased the incidence of mild hypoglycaemia from 48.3% to 32.2% (ARR 16.1%)[32]. The incidence of mild hypoglycaemia in AMC and Jessa were, respectively, 15.2% and 19.6%. Hence, for a decrease of mild hypoglycaemia from 27% to 20% (ARR 7%), a study with a two-sided confidence level of 5% (alpha error) and a statistical power of 90% (beta error level 10%) will require 769 patients in each arm.

In order to be powered for the primary outcome measure (GPI) and incidence of mild hypoglycaemia (safety), the study will therefore recruit 775 patients in each arm of the study to take into account the patients with an unanticipated early discharge from the ICU, leading to a total of 1550 patients. With this number of patients to be included, the LOGIC-2 study will have a 75% power to detect a 2000 EUR cost difference (one-sided alpha: 0.05, mean cost comparator 17000 EUR (SD 18000), mean cost intervention 15000 (SD 16000))[41].

All adult patients (≥18 years) admitted to the ICU with an expected stay of at least two days and already receiving or potentially needing insulin infusion for blood glucose control are eligible for the study.

The study protocol and patient follow-up will be stopped at ICU discharge or when the patient starts to eat. In order to guarantee the safety of the patient, the study can further be stopped in case of:

- Recurrent severe hypoglycaemic episodes (blood glucose < 40 mg/dL or 2.2 mmol/L) under treatment with the computer algorithm, as judged by the investigator or treating physician
- Refractory hyperglycaemia (blood glucose > 180 mg/dL or 10 mmol/L) under treatment with the computer algorithm
- Any change in condition that compromises the safety of the patient, as judged by the investigator or treating physician

Further, electronic CRFs for the study will be prepared and used for data collection. A final report will be written after completing the clinical study and all results will be offered to the appropriate scientific journals.

The Good Clinical Practice (GCP) quality standard was followed in the design of both the clinical study and the LOGIC-Insulin software to protect the human rights for the patients who will be enrolled in the study and to guarantee the safety and efficacy of the medical device under study. Finally, the ethical principles described in the Declaration of Helsinki are also met and the formulations as mentioned in the ISO 14155 were followed.

4.5 Summary of training experience

The medical and nursing staff of the trial centres will be intensively trained (2 hours general introduction for each shift and at the bedside for the first two patients for each nurse) by the clinical investigators or their delegates.

Specific attention will be paid to the risks in the study, notably hypoglycaemia or refractory hyperglycaemia. As included in the protocol, in the case of recurrent hypoglycaemic episodes or hyperglycaemia that cannot be controlled under the LOGIC-Insulin software or any change in condition compromising the safety of the patient, the patient can be withdrawn from the study. The investigators have to be notified as soon as possible by the treating nurse or physician.

Contra-indications for the study are as follows:

- Absence of one of the components of the TGC strategy: arterial line for blood sampling, accurate syringe pump for the administration of insulin, blood gas analyser for blood glucose measurements.
- The patient is eating.

These contra-indications are already standard practices for TGC in the participating departments. Specific warnings regarding the risks, notably hypoglycaemia and refractory hyperglycaemia, are not necessary as the study will only be executed by nurses with expertise in TGC. Trainee nurses and recently appointed nurses will not be allowed to treat patients independently under the study protocol.

4.7 Informed consent

Copies of the approved informed consents for UZ Leuven and Jessa Hospital Hasselt are added in **Appendices 7 and 8**. The informed consents for AMC Amsterdam will be sent to the IRB after approval of current file by the AMC Instrumenteel Bedrijf.

4.8 Case Report Form (CRF)

The CRF will be made electronically available. As formulated in ISO 14155-2, the following information will be recorded for each patient entering the study:

a) the date, place and identification of the investigation, including the version number of the Clinical Investigation Plan (CIP);
b) identification of the subject, date of enrolment, demographic data;
c) identification of the medical device by number of software version;
d) medical diagnosis for which the subject is to be treated with the device to be investigated together with any concomitant illness;
e) subject compliance information for concurrent procedures measures and for any emergency;
f) relevant previous medication and/or procedures;
g) subject baseline characteristics;
h) concomitant medication and/or procedures;
i) compliance with the inclusion/exclusion criteria;
j) dated clinical and non-clinical findings according to the CIP: all blood glucose levels, all insulin infusion rates, nutritional intake and medication rates at any time will be recorded in our patient database management system and exported to the study database;
k) procedural data;
l) subject assessment during the use of the device and follow-up with dates;
m) reported adverse events and adverse device effects with dates;
n) date of the end of follow-up;
o) signature of the clinical investigator at the completion of follow-up.

4.9 Clinical study

This section has already been discussed above:

- Rationale and justification of the clinical investigation: see **Introduction - section**
- Summary of background to study: see **Introduction - section**
- Pre-clinical testing: see **Section 3.6 Clinical data on device in question** and **Section 3.8: Summary and analysis of pre-clinical testing**
- Device and investigation risk analysis and risk assessment: see **Appendix 3 Risk management analysis**, **Section 3.7 Benefit/Risk analysis** and **Section 3.10 Description of biocompatibility and biological safety**.

5. CLINICAL INVESTIGATION PLAN: Investigation Parameters and Design

5.1 Objectives of clinical investigation

In succession of the LOGIC-1 RCT that showed the *non-inferiority* (equivalence) of the computer algorithm (LOGIC-Insulin) against the standard nurse-directed protocol for the normalisation of blood glucose concentrations in an expert ICU (i.e. the Leuven setting), the LOGIC-2 multi-centre RCT aims at demonstrating the *superiority* of the computer algorithm (LOGIC-Insulin) against the standard routine protocol for the normalisation of blood glucose concentrations in the ICU. The secondary objective of the study is to demonstrate cost-effectiveness of the implementation of the LOGIC-Insulin algorithm.

5.2 Investigation design

This is an international, pragmatic effectiveness multi-centre interventional RCT. In accordance with state-of-the-art trial design it will consist of a screening, an intervention and a predefined follow-up assessment.

In the screening, an ICU-population, representative for most critically ill patients in developed countries, will be sought. For planned ICU admissions after elective procedures, the patient him/herself will be asked for informed consent beforehand. Only in the case of emergency ICU admissions deferred informed consent will be asked from the closest family member (legal representative). This will be done by a strict procedure. The attending physician will do the assessment of the patient for study eligibility within the time frame of two hours. However, it will be impossible to obtain a written informed consent within two hours after emergency admission. Therefore, if eligible, the patient will be randomised into the study and informed consent will be asked within the time frame of 24 hours (deferred informed consent) as blood glucose control has to be initiated on admission to treat the hyperglycaemic peak, usually present on admission. In severely ill patients, usually the emergency admissions, this hyperglycaemic peak is even more pronounced.

A duplicate of the signed informed consent will be given to the patient or the legal representative. The patient's referring physician will also be informed about the patient's participation. The family member or the patient can withdraw from the trial, at any time, without impact on his treatment or penalty.

Random allocation will be done by a centralised computer to either the standard practice (control group) or the computer algorithm (intervention group). In both allocation groups the blood glucose control will be done during the entire ICU-stay, as long as indicated. However, the analysis of the blood glucose dynamics will only be done in a timeframe of maximal 14 days, to minimise the impact of outliers. The trial has an open-label design as the bedside nurses and physicians cannot be blinded for the treatment allocation. However, the outcome assessors will be blinded for the treatment

allocation. The follow-up for clinical and economic outcome measures will stop at 90-days post-randomisation.

In the follow-up assessment all routinely recorded data will be collected (source data). These data will be stored on the servers of the participating centres. The duration of storage of study data will be the minimally required duration in the current local hospital policy. In the clinical trial database the following endpoints will be recorded and stored for at least ten years after completion of the study.

- **Primary endpoint:**
  Adequacy of reaching and maintaining the target range for blood glucose, as assessed by the GPI (up to 14 days) ("Effectiveness of glycaemic control")

- **Secondary clinical endpoints:**
  - Incidence of mild hypoglycaemia (blood glucose level < 70 mg/dL or < 3.9 mmol/L) per patient (up to 14 days) ("Safety of glycaemic control")
  - Mean and median arterial blood glucose concentration (up to 14 days)
  - Adequacy of reaching and maintaining the target range for blood glucose, as assessed by the Hyperglycaemic index (HGI) (up to 14 days)
  - Time in target range (up to 14 days)
  - Time to target range (up to 14 days)
  - Daily maximal blood glucose difference, as a marker of blood glucose fluctuations (up to 14 days)
  - Incidence of severe hypoglycaemia (blood glucose level < 40 mg/dL or 2.2 mmol/L) per patient (up to 14 days)
  - Incidence of severe hypoglycaemia (blood glucose level < 40 mg/dL or 2.2 mmol/L) as a proportion of all blood glucose measurements (up to 14 days)
  - Incidence of mild hypoglycaemia (blood glucose level < 70 mg/dL or 3.9 mmol/L) as a proportion of all blood glucose measurements (up to 14 days)
  - Interval between blood glucose measurements, as a marker of workload (up to 14 days)
  - Protocol compliance in the intervention group (up to 14 days): the number and proportion of patients in which the LOGIC-Insulin was not followed for a time period of at least 8 hours, which is the duration of one nurse shift.
  - Overrules in the intervention group (up to 14 days): the number and proportion of recommendations by the software that were overruled by the bed-side nurses
    - ✓ Minor overrules: absolute insulin dose difference of >0.1 and < 1IU/h
    - ✓ Major overrules: absolute insulin dose difference of >= 1IU/h
    - ✓ Major overrules will also be qualitatively assessed
  - Incidence of new infections in the ICU (up to 90 days): The diagnosis of "new infection" will be based on the administration of antibiotics, beyond the prophylactic scope
  - Length of stay in the ICU (up to 90 days)

- Length of stay in the hospital (up to 90 days)
- Mortality in the ICU (up to 90 days)
- Mortality in the hospital (up to 90 days)
- Landmark 90-day mortality.

- **Secondary economic endpoints:**
  - All direct medical costs from a healthcare payer's perspective (time horizon from randomisation to hospital discharge, up to 90 days)
  - The EuroQol-5D questionnaire at ICU and hospital discharge, and at 90 days.

The time window to calculate the measures of glycaemic control (GPI, incidence of hypoglycaemia, mean/median glycaemia, HGI, time in target, daily maximal blood glucose difference, time to target, episodes of hypoglycaemia, interval between blood glucose measurements, protocol compliance) will start with the first blood glucose measurement in the ICU and stop when tight blood glucose is not anymore indicated up to a maximum of 14 days.

5.3 Numbers of subjects

The study has been conceived as a superiority trial for improving the effectiveness of blood glucose control, measured by the GPI (primary outcome measure) and the time-in-target. The LOGIC-1 single centre study[32] showed that, compared to standard care, a computer algorithm decreases the GPI from median 12.4 (IQR 8.2-18.5) to 9.8 (IQR 6.0-14.5), hence an observed difference of 2.6. A retrospective analysis of 461 patients from the AMC, Amsterdam showed a GPI of median 13 (IQR 6-24) and of 739 patients from the Jessa Hospital Hasselt revealed a GPI of 24 (IQR 17-33). A study, in which all centres would include a similar number of patients, with a confidence level of 5% (alpha error) and a statistical power of 90% (beta error level 10%) will require 458 patients in each arm of the study to decrease GPI from mean 21 to 19 (sigma 14) in a two-sided test. For an increase of the time-in-target from 45% to 50% with a sigma of 29%, a study with a two-sided confidence level of 5% (alpha error) and a statistical power of 90% (beta error level 10%) will require 707 patients in each arm.

The study will also be powered to detect differences in the incidence of mild hypoglycaemia (<70 mg/dL or < 3.9 mmol/L), a safety outcome variable. In the LOGIC-1 RCT the use of the LOGIC-Insulin algorithm decreased the incidence of mild hypoglycaemia from 48.3% to 32.2% (ARR 16.1%)[32]. The incidence of mild hypoglycaemia in AMC and Jessa were, respectively, 15.2% and 19.6%. Hence, for a decrease of mild hypoglycaemia from 27% to 20% (ARR 7%), a study with a two-sided confidence level of 5% (alpha error) and a statistical power of 90% (beta error level 10%) will require 769 patients in each arm.

In order to be powered for the primary outcome measure (GPI) and incidence of mild hypoglycaemia (safety), the study will therefore recruit 775 patients in each arm of the study to take into account the patients with an unanticipated early discharge from the ICU, leading to a total of 1550 patients.

This number of patients will generate a 75% power to detect a 2000 EUR cost difference (one-sided alpha: 0.05, mean cost comparator 17000 EUR (SD 18000), mean cost intervention 15000 (SD 16000))[41].

5.4 Duration of study

Depending on the enrolment efficiency, the study will need about 9 to 12 months for completion. This does not include the extensive briefing and training of the nursing staff for the LOGIC-Insulin algorithm.
The estimated start date is January 6, 2014. The last inclusion is planned before January 1, 2015. Data interpretation and publication will take 10 months. The follow-up period starts immediately after finishing the study.

5.5 Criteria for subject selection

Inclusion criteria can be summarized as follows:

- Signed informed consent from patient or the next of kin, obtained as soon as possible after admission to the ICU (<24 hours). Blood glucose control is an important issue in the treatment of critically ill patients and should be initiated immediately after admission to the ICU. If the patient or the next of kin does not agree with the study and does not sign the informed consent within the first 24 hours after admission to the ICU, the patient will be withdrawn from the study,
- Patients admitted to the ICU with an expected stay of at least two days and already receiving or potentially needing insulin infusion for blood glucose control (these patients should already have or need an arterial and central venous line),
- Patients should be older than 18 years.

Exclusion criteria are:

- Age under 18 years,
- Patients deemed not critically ill ("monitoring only" patients, no arterial or central line),
- Patients expected to die within 12 hours (=moribund patients),
- Patients already enrolled in another intervention RCT,
- Pregnancy or lactating,
- Patients suffering from ketoacidotic or hyperosmolar coma on admission,
- Patients who have been previously included in the LOGIC-2 study,
- Allergy to insulin.

5.6 Criteria for withdrawal from study

Patients can leave the study at any time for any reason if they wish to do so without any consequences. When a patient or legal representative withdraws consent during ICU stay, the patient will go on to be clinically followed up without his data being analysed in the study. A separate patient log will register all patients withdrawn from the study.

End-of-care decisions in patients for whom further intensive care is considered to be futile will be taken in consensus by a group of at least two senior ICU physicians and the referring specialist, all blinded to study treatment allocation.

Withdrawal criteria:

- Recurrent severe hypoglycaemic episodes (blood glucose < 40 mg/dL or 2.2 mmol/L) under treatment with the computer algorithm, as judged by the investigator or treating physician,
- Refractory hyperglycaemia (blood glucose > 180 mg/dL or 10.0 mmol/L) under treatment with the computer algorithm,
- Any change in condition that compromises the safety of the patient, as judged by the investigator or treating physician.

5.7 Description and justification of hazards caused by invasive procedures that are not medically required (if applicable).

This section is not relevant for the medical device under study.

5.8 Description of general methods of diagnosis or treatment of the medical condition for which the investigation testing is being proposed.

Hyperglycaemia with critically ill patients is currently treated via intensive insulin therapy by the bedside nurse in the participating ICUs. Blood glucose target range in the Leuven and Hasselt ICU is 80-110 mg/dL (4.4-6.1 mmol/L) whereas a slightly higher target zone is applied in the Amsterdam ICU (90-145 mg/dL, 5.0-8.0 mmol/L). The insulin dose-adaptations are based on a guideline to stimulate intuitive and anticipating decision making.

5.9 Monitoring arrangements during the clinical investigation including request for direct access to source documents including extent of source data verification.

The first post-initiation visit will usually be made as soon as possible after enrolment has begun. At these visits, the monitor will compare the data entered into the CRFs with the hospital records (source documents). At a minimum, source documentation must be available to substantiate: subject

identification, eligibility, proper informed consent, and randomisation; adherence to protocol procedures; adequate reporting and follow-up of adverse events; administration of concomitant medication; date of study completion, discontinuation from treatment, or withdrawal from the study.

Simultaneously, all data will be recorded directly into the patient data management system (PDMS) (source document). All entries into the PDMS will be identifiable and reviewable by the monitor. Direct access to other source documents such as medical records must be allowed for the purpose of verifying that the data recorded in the CRF are consistent with the original source data. Findings from this review of CRFs and source documents will be discussed with the investigators.

The sponsor (KU Leuven – Leuven Research & Development) expects that, during monitoring visits, the relevant investigational staff and source documentation will be available for review. The monitor will meet with the investigators on a regular basis during the study to provide feedback on the study conduct.

6. CLINICAL INVESTIGATION PLAN: Data Collection/Analysis/Statistics

6.1 Description of endpoints

6.1.1 Primary endpoint

Adequacy of reaching and maintaining the target range for blood glucose, as assessed by the GPI (up to 14 days) ("Effectiveness of glycaemic control")

6.1.2 Secondary clinical endpoints

- Incidence of mild hypoglycaemia (blood glucose level < 70 mg/dL or 3.9 mmol/L) per patient (up to 14 days) ("Safety of glycaemic control")
- Mean and median arterial blood glucose concentration (up to 14 days)
- Adequacy of reaching and maintaining the target range for blood glucose, as assessed by the Hyperglycaemic index (HGI)(22) (up to 14 days)
- Time in target range (up to 14 days)
- Time to target range (up to 14 days)
- Daily maximal blood glucose difference, as a marker of blood glucose fluctuations (up to 14 days)
- Incidence of severe hypoglycaemia (blood glucose level < 40 mg/dL or 2.2 mmol/L) per patient (up to 14 days)
- Incidence of severe hypoglycaemia (blood glucose level < 40 mg/dL or 2.2 mmol/L) as a proportion of all blood glucose measurements (up to 14 days)
- Incidence of mild hypoglycaemia (blood glucose level < 70 mg/dL or 3.9 mmol/L) as a proportion of all blood glucose measurements (up to 14 days)
- Interval between blood glucose measurements, as a marker of workload (up to 14 days)
- Protocol compliance in the intervention group (up to 14 days): the number and proportion of patients in which the LOGIC-Insulin was not followed for a time period of at least 8 hours, which is the duration of one nurse shift.
- Overrules in the intervention group (up to 14 days): the number and proportions of recommendation by the software that were overruled by the bed-side nurses
    - Minor overrules: absolute insulin dose difference of >0.1 and < 1IU/h
    - Major overrules: absolute insulin dose difference of >= 1IU/h
    - Major overrules will also be qualitatively assessed
- Incidence of new infections in the ICU (up to 90 days): The diagnosis of "new infection" will be based on the administration of antibiotics, beyond the prophylactic scope
- Length of stay in the ICU (up to 90 days)
- Length of stay in the hospital (up to 90 days)

- Mortality in the ICU (up to 90 days)
- Mortality in the hospital (up to 90 days)
- Landmark 90-day mortality

6.1.3 Secondary economic endpoints

- All direct medical costs from a healthcare payer's perspective (time horizon from randomisation to hospital discharge, up to 90 days)
- The EuroQol-5D questionnaire at ICU and hospital discharge, and at 90 days.

The time window to calculate the measures of glycaemic control (GPI, incidence of hypoglycaemia, mean/median glycaemia, HGI, time in target, daily maximal blood glucose difference, time to target, episodes of hypoglycaemia, interval between blood glucose measurements, protocol compliance) will start with the first blood glucose measurement in the ICU and stop when tight blood glucose is not anymore indicated up to a maximum of 14 days.

6.2 Description and justification of statistical design, method and analytical procedures

The study will use a prospective, randomised, controlled and parallel-group design. On admission patients will be randomly assigned to nurse-directed blood glucose control or to LOGIC-insulin guided blood glucose control. At ICU admission, consecutive patients will be randomly assigned to one of these two treatment groups using a centralized computer randomisation. Randomisation will be done in a 1:1 ratio in permuted blocks and stratified according to (1) centre and (2) primary diagnostic category on admission (cardiac surgery, medical, transplantation, other surgery & procedure). Block size will be unknown to the bedside nurses and physicians. Randomisation of cardiac surgery patients will stop after they have reached 60% of the total study population across all centres. All centres will include at least 250 patients in the study.

Routine state-of-the-art intensive care will be provided for each patient and trial related activities will not interfere with regular patient care. Blood samples will be retrieved from an arterial line, available for routine diagnostic and monitoring procedures in all patients. Immediately upon admission, intravenous infusion of insulin is started and the target blood glucose levels are 80-110 mg/dL (4.4-6.1 mmol/L) in the Leuven and Hasselt ICUs and 90-145 (5.0-8.0 mmol/L) in Amsterdam (routine care). In case of computer algorithm randomisation blood glucose values will be provided as requested by the algorithm and the insulin infusion rate will be adopted according to the advice suggested by the algorithm. The nurse always has to confirm this software advice. At all times the nurse can overrule this advice. In case of standard care, blood glucose values will be provided as required by the current best clinical practice of the local paper-based intuitive protocol.

The study protocol will stop at ICU discharge and patient follow-up at hospital discharge. The landmark 90-day survival status will be assessed through the official national registries.

6.2.1 Analysis principles

- All analyses will be conducted on an intention-to-treat basis.
- We will not impute missing values unless specified otherwise. We will report the number of observations used in the analysis.
- P-values will not be adjusted for multiplicity. However the outcomes are clearly categorized by degree of importance (primary to secondary) and the number of subgroup analyses are pre-specified.

6.2.2 Baseline characteristics

Description of baseline characteristics will be presented by randomised treatment group in a table. Discrete variables will be summarised using frequencies and percentages. Percentages will be calculated according to the number of patients for whom data is available. Continuous variables including durations will be summarised by use of standard measures of central tendency and dispersion, either mean and SD, or median and interquartile range (IQR).

The following variables will be presented in the baseline characteristics table:

- Number of patients
- Age
- Gender
- Height, weight, BMI
- APACHE-II score
- Diagnosis group
- Sepsis on admission (Y/N)
- History of diabetes (Y/N, type, R/diet, R/OAD, R/insulin, HbA1c if available)
- Insulin drip at admission (Y/N)
- Renal replacement therapy preadmission (Y/N)
- Mechanical ventilatory support at admission (Y/N)
- Glycaemia on admission
- Hypoglycaemia (<40 mg/dL or 2.2 mmol/L) on admission
- Blood lactate on admission
- EuroQoL-5 by proxy on admission.

6.2.3 Primary outcome analysis regarding glycaemic control

GPI during the study intervention (censored at 14 days) of control group versus intervention group will be compared by a non-parametric test (Mann-Whitney U). 95% Confidence Interval and p-value will be calculated

6.2.4 Secondary outcome analyses regarding glycaemic control

- Not normally distributed continuous variables of effectiveness of glycaemic control (HGI, time to target, and blood glucose variability) will be compared between treatment groups by a non-parametric test (Mann-Whitney U) and 95% Confidence Interval and p-value will be calculated
- Normally distributed continuous variables (mean glycaemia and sampling interval as a marker of workload) will be compared between treatment groups by the t-test and 95% Confidence Interval and p-value will be calculated
- Proportions, Time in target (effectiveness marker) and hypoglycaemia <70 mg/dL, <60 mg/dL and <40 mg/dL (<3.9 mmol/L, <3.3 mmol/L and <2.2 mmol/L) per patient and per samples (safety marker), of control group versus intervention group will be compared by Chi square (Fisher exact) test and 95% Confidence Interval and p-value will be calculated

The secondary outcome analyses regarding glycaemic control will be represented in a table format

6.2.5 Secondary outcome analyses regarding post-randomisation interfering factors for glycaemic control

- Daily insulin dose and daily carbohydrate intake of control group versus intervention group will be compared by a non-parametric test (Mann-Whitney U) and 95% Confidence Interval and p-value will be calculated
- Proportional number of days that the patient was on vasopressor/inotropic support, was receiving steroid medication, was on mixed-bag parenteral nutrition and was on antibiotics will be compared between groups by Chi square (Fisher exact) test and 95% Confidence Interval and p-value will be calculated

6.2.6 Tertiary analysis regarding protocol compliance with LOGIC-Insulin software

This will be regarded as a tertiary analysis as the analyses will only be done in the intervention group. Hence, the results will only be descriptive:

- Proportion of patients in whom the software was not used for at least 8 hours
- Proportion of patients in whom a minor and major overruling of the software advice occurred
- Proportion of patients in whom the major overruling was justified to avoid hypoglycaemia

6.2.7 Clinical outcome measures (secondary level)

- The clinical outcome measures will be presented as a separate table
- Length of stay in ICU and hospital will be compared between treatment groups by a non-parametric test (Mann-Whitney U) and 95% Confidence Interval and p-value will be calculated
- Proportion of patients with a new infection in the ICU of control group versus intervention group will be compared by Chi square (Fisher exact) test and 95% Confidence Interval and p-value will be calculated

- Ventilator-free days per patient, censored at 14 days, of control group versus intervention group will be compared by a non-parametric test (Mann-Whitney U) and 95% Confidence Interval and p-value will be calculated
- Proportion of patients who died in the ICU, the hospital and at 90 days of control group versus intervention group will be compared by Chi square (Fisher exact) test and 95% Confidence Interval and p-value will be calculated
- EuroQoL-5 scores at ICU and hospital discharge and at 90 days of control group versus intervention group will be compared by a non-parametric test (Mann-Whitney U) and 95% Confidence Interval and p-value will be calculated

6.2.8 Planned subgroup analyses for primary outcome measure and secondary outcome measures regarding glycaemic control

- Cardiac surgery admission versus other
- Medical admission versus other
- Sepsis on admission versus other
- Diabetes mellitus on admission versus other
- Subgroup analysis per study centre
- Subgroup analysis for the two blood glucose target ranges

6.2.9 Health economic assessment

Cost-consequence, cost-effectiveness and cost-utility analyses will also be undertaken as part of the study. The objective of these economic evaluations is to assess whether the costs of implementing the LOGIC-Insulin computer algorithm for blood glucose control are justified by reductions in hospitalisation costs and/or by improvements in patient outcomes.

The first planned analysis will be a cost-consequence analysis of the software implementation. Secondly, a cost-effectiveness analysis with the GPI (primary outcome), hypoglycaemia, ICU-acquired infection and survival at hospital discharge and at 90 days will be done. Finally, a cost-utility analysis is planned in which the health benefits are quantified in terms of quality-adjusted life years (QALYS), and measured by the EuroQoL-5D at hospital discharge and at 90 days.

The perspective for the analyses will be the healthcare payer's perspective. The time horizon for the economic evaluation parallels the follow-up duration of the LOGIC-2 trial. For survival and EuroQoL-5D this will be 90 days. However, cost-data will only be available during hospital stay. Hence, the minimal time horizon will be hospital stay.

The cost estimation will be done in two ways. For all hospitals resource use will be the basis of the cost estimation as this international study is confronted with two healthcare systems. Commonly accepted cost drivers will be used herein (days on ventilator, days with infection, days in ICU and days in hospital). Data on resource use will come from the study database and source databases (patient data management system, hospital information system). For the Belgian hospitals an additional micro-costing approach can be used as most medical interventions and pharmacy costs are

recorded in the administrative database of the hospitals. Only, direct healthcare-related costs will be taken into account. Costs will not be discounted as the time horizon is only 90 days.

All economic analyses will be done on an intention-to-treat basis. The cost-consequence analysis will report mean differences with 95% CIs in resource use and hospital costs between the treatment groups. In the cost-effectiveness analysis costs per death averted and costs per adverse event (hypoglycaemia, ICU-acquired infection) averted will be reported.

To take into account generalisability problems and uncertainty, respectively subgroup and sensitivity analyses will be done. With the availability of patient level data on costs and effects, uncertainty can be summarized in the incremental cost-effectiveness ratio (ICER) as a confidence interval. Non-parametric bootstrapping will be used to calculate the confidence intervals, since cost data are not normally distributed.

Subgroup analyses will comprise: cardiac surgery versus other, medical versus other, sepsis on admission and diabetes mellitus on admission. Also a subgroup analysis per study centre and blood glucose target will be done. As the comparator (nurse-directed glycaemic control) in this study is approaching gold standard, an analysis under real-world conditions with a higher incidence of hyperglycaemia, hypoglycaemia and blood glucose variability will be done (scenario analysis). Further, multi-way probabilistic sensitivity analyses (Markov model) will take into account the imprecision in all input variables. The ranges and thresholds for the probabilistic modelling will be based on the data distribution. To avoid bias and imprecision due to missing data, multiple data imputation will be done.

6.2.10 Further exploratory analyses

In order to answer questions of peer-reviewers, to explain possible differences between study groups or in certain subgroups or for other reasons ancillary exploratory analyses can be done.

6.3 Procedures for data collection, review, cleaning, and issuing and resolving queries, if appropriate.

Screening procedures will take place as soon as possible after admission to the ICU (<2 hours). The patient, or the closest relative of the patient will be informed about the trial and written informed consent will be obtained. A screening number will be assigned to the patient in ascending order. The following data will be recorded in the case record form (CRF) after informed consent is obtained:

- Patient number
- Patient name: for reasons of data integrity and internal control during data input, the patient name is stored in a separate table linked to the eCRF. However, these data will only be accessible for the authorized local research staff and the principal database manager on a login/password base. When the database is finalized, the identity data will be detached from the eCRF and stored at the local site.
- Anonymised hospital ID numbers: the key to the original patient ID number will be kept by the local study site

- Randomisation group
- Randomisation number
- Unit & bed number
- On admission diagnosis group
- Date of birth & age
- Gender
- Admission date & time
- Inclusion date & time
- Height, weight, BMI
- APACHE-II score
- Diagnosis
- Sepsis on admission (Y/N)
- History of diabetes (Y/N, type, R/diet, R/OAD, R/insulin, HbA1c if available)
- Insulin drip at admission (Y/N)
- Renal replacement therapy preadmission (Y/N)
- Mechanical ventilatory support at admission (Y/N)
- Glycaemia on admission
- Hypoglycaemia (<40 mg/dL or 2.2 mmol/L) on admission
- Blood lactate on admission
- Investigation site
- Investigation ID
- Check for inclusion criteria
    - Patients admitted to the ICU with an expected stay of at least 48 hours and already receiving or potentially needing insulin infusion for blood glucose control. These patients should already have or need an arterial and central venous line;
    - Patients should be 18 years or older.
- Check for exclusion criteria
    - Not critically ill
    - Age under 18 years
    - Patients already enrolled in another intervention RCT
    - Patients expected to die within 12 hours (=moribund patients)
    - No arterial line or central venous line needed
    - Pregnancy or lactating
    - Patients suffering from ketoacidotic or hyperosmolar coma on admission
    - Patients who have been previously been included in the LOGIC-2 study
    - Allergy to insulin
- Informed consent (patient or relative, number).

A separate log file will be kept for the patients, who were not included in the study. These data will not be used for analysis, but are required to construct an adequate patients' flow diagram (CONSORT diagram). Only the following data will be recorded:

- Patient name: will only be kept at the local study Site
- Anonymised hospital ID numbers: the key to the original patient ID number will be kept at the local study Site
- Age, gender
- Admission date
- Diagnostic group
- Reason for non-inclusion.

In the admission file of the CRF the data on the end of the study will also be included.

- End of study date, time and reason (stopping criteria)
- ICU discharge date, ICU length of stay (to a maximum of 90 days)
- Hospital discharge date, hospital length of stay (to a maximum of 90 days)
- Outcome (=died during hospitalization within 90 days, Y/N), date of death
- EuroQoL-5 score at ICU and hospital discharge, and at 90 days
- File completed (Y/N)
- Authorization for completion.

Simultaneously, all data from the patient data management system, which are used in routine clinical care in the three hospitals, will also be kept for the study patients. Additionally, the data that are highly relevant for the study will be included in the CRF on a daily basis.

- Medication that interferes with insulin sensitivity
    - Daily cumulative insulin (in IU)
    - Daily cumulative dose of adrenaline, noradrenaline, dopamine, dobutamine and milrinone (in mg)
    - Steroid medication (type, dose, mg, route)
- Nutrition
    - Parenteral nutrition (cumulatively administered during 1 day)
    - Enteral nutrition (cumulatively administered during 1 day)
- Antibiotics administered on a daily basis (type, dose, mg, route)
- Mechanical ventilation (Y/N)
- Blood glucose values
    - Only date & time stamped arterial blood glucose values will be included
    - For blood glucose values within 20 minutes time interval, the first blood glucose value will be omitted, except in the case of hypoglycaemia (<40 mg/dL or 2.2 mmol/L) treatment
- Hypoglycaemia report (will be reported per hypoglycaemic event)
    - Minimum glycaemia

- Maximum glycaemia
- Duration (in hours) from hypoglycaemia to death
- Date and time of hypoglycaemia
- Cause of hypoglycaemia
- Action undertaken
- Insulin drip decrease/stop (Y/N/no insulin)
- Bolus glucose (Y/N)
- Glycaemia > 40 mg/dL (2.2 mmol/L) within 1h, 2h, 3h, 4h (Y/N/no value)
- Immediate consequences (Y/N/unclear)
  - Perspiration (Y/N)
  - Epilepsy within 8h ((Y/N/unclear)
  - Severe arrhythmia or shock (Y/N)
  - Death within 24h (Y/N)
  - Loss of consciousness (Y/N/sedation).

6.4 Recording and reporting procedures of clinical investigation plan deviations, if appropriate

Electronic CRFs for the study will be prepared and used for data collection. The patient and any biological material obtained from the subject will be identified by subject number and trial identification number. Appropriate measures such as encryption or deletion will be enforced to protect the identity of human subjects in all presentations and publications. For screening failure patients no data will be entered into the database.

7. CLINICAL INVESTIGATION PLAN: Safety Reporting

7.1 Definitions of adverse events and adverse device effects

Adverse events are defined as any undesirable experience (including an abnormal finding) occurring to a patient during a clinical trial, whether or not considered related to the investigational device. All adverse events reported spontaneously by the patient or observed by the investigators or their staffs will be recorded.

For the scope of the study we further define the following events as ‘adverse device effects’:

- Severe hypoglycaemic episodes (< 40 mg/dL or 2.2 mmol/L) under treatment with the computer algorithm,
- Refractory hyperglycaemia under treatment with the computer algorithm.

Finally, any occurrence that is new in onset or clinically significantly aggravated in severity or frequency from the baseline condition, or abnormal results of diagnostic procedures, including laboratory test abnormalities, are defined as adverse event.

Adverse events related to disease progression and surgical procedures as part of admission to the ICU are not considered reportable adverse events.

Note 1: It is a standard procedure in our ICU that all patients are clinically and neurologically evaluated upon discharge from the ICU.

Note 2: The sponsor collects adverse events starting with the signing of the informed consent.

7.2 Definitions of serious adverse events and serious adverse device effects

A serious adverse event (SAE) is any untoward medical occurrence that:

- results in death
- is life threatening (at the time of the event)
- requires hospitalization or prolongation of existing inpatients’ hospitalization
- results in persistent or significant disability or incapacity
- is a congenital anomaly or birth defect
- is a new event of the trial likely to affect the safety of the patients.

As this study will be performed in critically ill patients, SAE are expected to happen often. We will report all unanticipated severe adverse device effects (USADE’s) to the FAGG and through the web portal *ToetsingOnline* to the accredited ethics committee that approved the clinical trial protocol, according to the requirements of that ethics committee. All other SAEs (i.e., not device–related) are

recorded in an overview list (line-listing) that will be submitted once every year to the ethics committee. This line–listing provides an overview of all SAEs from the study, accompanied by a brief report highlighting the main points of concern.

For the scope of the study we further define the following events as “serious adverse device effects”:

- Recurrent hypoglycaemic episodes (blood glucose < 40 mg/dL or 2.2 mmol/L) under treatment with the computer algorithm, as judged by the investigator or treating physician,
- Any change in condition that compromises the safety of the patient, as judged by the investigator or treating physician.

Note 1: It is a standard procedure in our ICU that all patients are clinically and neurologically evaluated upon discharge from the ICU.

Note 2: The sponsor collects serious adverse events starting with the signing of the informed consent.

Note 3: Medical and clinical judgement should be exercised in deciding whether expedited reporting is also appropriate in situations other than those listed above. For example, important medical events may not be immediately life threatening or result in death or prolonged hospitalization but may jeopardize the subject or may require intervention to prevent one of the outcomes listed in the definition above. Any adverse event is considered a serious adverse event if it is associated with clinical signs or symptoms judged by the investigator to have a significant clinical impact.

7.3 Details of procedures of those events to be reported to the relevant Ethics Committees and Competent Authority, including timelines

Before the start of the study, the investigator (or sponsor where required) will provide the Ethics Committee (EC) and the Competent Authority (CA) with current and complete copies of the following documents:

- Final protocol and, if applicable, amendments,
- Sponsor-approved informed consent form,
- Investigator’s brochure,
- Information on compensation for study-related injuries,
- Investigator’s curriculum vitae,
- Any other documents that the EC or CA requests to fulfil its obligation.

This study will be undertaken only after the EC (and Instrumenteel Bedrijf AMC for The Netherlands) has given its full approval and if the FAGG did not raise any objections against the start of the study.

During the study the investigator (or the sponsor where required) will send the following documents to the EC and CA for their review and approval, where appropriate:

- protocol amendments,
- revisions to the informed consent form,
- revisions to the compensation for study-related injuries,
- investigator’s brochure amendments or new editions,
- summary of the status of the study, if required,
- reports of adverse events that are serious and associated with the study intervention,
- new information that may adversely affect the safety of the subjects or the conduct of the study,
- deviations from or changes to the protocol to eliminate immediate hazards to the subjects,
- report of deaths of subjects under the investigator’s care,
- notification if a new investigator is responsible for the study.

For protocol amendments that increase subject risk, the amendment and applicable informed consent form revisions must be submitted promptly to the EC and CA for review and approval before the implementation of the changes. For non-substantial amendments, approval or only notification are required.

At the end of the study, the investigator (or sponsor where required) will notify the EC and CA about the study completion.

7.4 List of foreseeable adverse events and adverse device effects, likely incidence, mitigation and/or treatment.

It is virtually unavoidable that hypoglycaemic episodes are evoked when applying intensive insulin therapy towards a narrow target blood glucose. Therefore, hypoglycaemia is defined as a ‘foreseeable’ adverse event. However, provided that hypoglycaemia is diagnosed and treated promptly and adequately, the latter implying prevention of over-correction, the short-term benefit of preventing hyperglycaemia in adult ICU patients seems to outweigh the short-term risk of hypoglycaemia. Moreover, to prevent or to treat (potentially severe) hypoglycaemia, the LOGIC-Insulin algorithm also recommends to infuse glucose calories (as a bolus) when hypoglycaemia is expected or observed.

7.5 Emergency contact details for reporting serious adverse events and serious adverse device effects to the Sponsor.

The names of the individuals (and the corresponding telephone numbers) who should be contacted regarding safety issues or questions regarding the study are listed below:

- Dieter Mesotten: +32 497 54 66 20
- Tom Van Herpe: + 32 494 89 21 36
- Guy Veraghtert: +32 499 25 37 82

In case of unavailability of the above mentioned individuals, delegates will be assigned.

8. CLINICAL INVESTIGATION PLAN : Other

8.1 Reference to Insurance coverage in case of injury

The insurance document is added in **Appendix 9**.

8.2 Device accountability procedures

The Information Technology (IT) responsibilities in general and for the medical device under study in particular are structured as follows:

- **KU Leuven:**
    - Prof. Bart Van den Bosch is the head of the (IT) service department and, accordingly, is the end responsible for all IT aspects in the University Hospitals Leuven.
    - Reinoud Reynders is the head of the Systems & Operations division of the University Hospitals Leuven. He is responsible for all general computer support, control of the network, central IT services, standard software, etc.
    - Tom Van Herpe holds the “master-file” of the updated LOGIC-Insulin algorithm.
    - Guy Veraghtert holds the “master-file” of the new LOGIC-Insulin graphical user interface.

- **Jessa Hospital Hasselt:**
    - Hilde Goossens is the head of the (IT) service department and, accordingly, is the end responsible for all IT aspects in Jessa Hospital Hasselt.
    - Jan Jansen is the project manager ICT in Jessa Hospital Hasselt
    - Tom Van Herpe holds the “master-file” of the updated LOGIC-Insulin algorithm.
    - Guy Veraghtert holds the “master-file” of the new LOGIC-Insulin graphical user interface.

- **AMC Amsterdam:**
    - Edwin Schuchmann is the head of the ICT department in AMC Amsterdam
    - Edward J. Feith is system / application manager in AMC Amsterdam
    - Tom Van Herpe holds the “master-file” of the updated LOGIC-Insulin algorithm.
    - Guy Veraghtert holds the “master-file” of the new LOGIC-Insulin graphical user interface.

The investigator is responsible for ensuring that the study device is used in accordance with applicable regulations and the approved protocol.

8.3 Ethics and informed consent procedures

The Participating Sites acknowledge that the Study can and will be conducted only on the basis of prior/deferred informed consent by the Subjects, or their legal representatives, to participate in the Study. The Participating Sites shall obtain a signed informed consent form for all patients prior/during to their enrollment and participation in the Study in compliance with all applicable laws, regulations and the approval of the (local) Ethics Committee.

More specifically, for planned ICU admissions after elective procedures, the patient him/herself will be asked for informed consent beforehand. Only in the case of emergency ICU admissions deferred informed consent will be asked from the closest family member (legal representative). This will be done by a strict procedure. The attending physician will do the assessment of the patient for study eligibility within the time frame of two hours. However, it will be impossible to obtain a written informed consent within two hours after emergency admission. Therefore, if eligible, the patient will be randomised into the study and informed consent will be asked within the time frame of 24 hours (deferred informed consent) as blood glucose control has to be initiated on admission to treat the hyperglycaemic peak, usually present on admission. In severely ill patients, usually the emergency admissions, this hyperglycaemic peak is even more pronounced.

A duplicate of the signed informed consent will be given to the patient or the legal representative. The patient's referring physician will also be informed about the patient's participation. The family member or the patient can withdraw from the trial, at any time, without impact on his treatment or penalty.

The Participating Sites shall retain such informed consents in accordance with the requirements of all applicable regulatory agencies and laws.

During the patient's participation in the study adequate medical care is provided to the patient by the treating physician of the dept. intensive care medicine. The treating physician informs the investigator if any disease or condition interferes with the study or the safety of the patient.

The investigator is accountable for the conduct of the study. If any responsibilities are delegated, the investigator should maintain a list of appropriately qualified persons to whom he has delegated specified significant study-related duties.

8.4 Procedure for early termination or suspension of the investigation giving criteria and risk analysis.

The study is considered completed with the last visit of the last subject undergoing the study. Reasons for the early termination may include but are not limited to:

- major safety concerns,
- failure to comply with the protocol or the GCP guidelines,
- inadequate recruitment of subjects.

8.5 Procedure for clinical investigation plan amendments

Neither the sponsor nor the investigator will modify the clinical investigation plan without a formal amendment. All protocol amendments must be issued by the sponsor, and signed and dated by the investigator. Protocol amendments must not be implemented without prior approval of the Ethics Committee (EC) and Competent Authority (CA). When changes involve only logistic or administrative aspects of the study, the EC and CA only need to be notified.

8.6 Final report and publication policy.

A final report will be written after completing the clinical study. As formulated in ISO 14155-1, this report will include an identification of the LOGIC-Insulin software, a description of the methodology and design of the clinical investigation, any deviations from the clinical investigation plan, data analysis with any statistical analysis and a critical appraisal in relation to the aims of the investigation.

Furthermore, all results will be offered to scientific journals.

Appendix 1: Signed statement

We here declare that the medical device under study complies with the essential requirements except with regard to those aspects of the medical device which are to be investigated; and that, in respect of those aspects, every precaution has been taken to protect the health and safety of the subject.

07-01-2014

D. MESOTTEN

Prof. Dr. Dieter Mesotten

Signature Clinical Investigator

Appendix 2: Copy of the Ethics committee approval

COMMISSIE MEDISCHE ETHIEK VAN DE UNIVERSITAIRE ZIEKENHUIZEN KULEUVEN
U.Z. GASTHUISBERG
HERESTRAAT 49
B-3000 LEUVEN (BELGIUM)

KATHOLIEKE
UNIVERSITEIT
LEUVEN

Aan Prof. D. Mesotten
Intensieve Zorgen, UZ Leuven

ONS KENMERK ML9517
LEUVEN, 31 juli 2013

Nurse-directed versus algorithm-assisted tight blood glucose control in adult critically ill patients: the logic-2 multi-centre randomized controlled trial. S55613

Belgisch Nummer B322201318068

S55613

DEFINITIEF GUNSTIG ADVIES

Geachte Collega,

De Commissie Medische Ethiek van de Universitaire Ziekenhuizen K.U.Leuven heeft vermeld protocol onderzocht en besproken op haar vergadering van 26 juni 2013.

Na inzage van de bijkomende informatie en/of aangepaste documenten met betrekking tot dit dossier is de Commissie van oordeel dat de voorgestelde studie, zoals beschreven in het protocol, wetenschappelijk relevant en ethisch verantwoord is. Na raadpleging van de ethische commissies van de andere deelnemende centra verleent ze dan ook een gunstig advies over deze studie.

Bij het beoordelen van dit dossier werd rekening gehouden met de documenten en informatie gerelateerd aan deze studie, ingediend op 6 juni 2013, 11 juni 2013 en 16 juli 2013.

Dit gunstig advies betreft:

Protocol : version 16 July 2013 dd LOGIC-2_v2_01

Patiëntendocumenten

- *IC LOGIC-2 UZLeuven patiënt versie 2 dd 16/07/2013*
- *IC LOGIC-2 UZLeuven vertegenwoordiger versie 2 dd 16/07/2013*
- *IC LOGIC-2 UZLeuven patiënt achteraf versie 1 dd 16/07/2013*
- *IC LOGIC-2 Jessa patiënt versie 2 dd 16/07/2013*
- *IC LOGIC-2 Jessa vertegenwoordiger versie 2 dd 16/07/2013*
- *IC LOGIC-2 Jessa patiënt achteraf versie 1 dd 16/07/2013*

De Commissie bevestigt dat ze werkt in overeenstemming met de ICH-GCP principes (International Conference on Harmonization Guidelines on Good Clinical Practice) en met de van toepassing zijnde wetten en regelgeving.

SECRETARIAAT: M. LEYS N. OPDEKAMP D. VAN MOLL M. VERBEECK H. HUYGHE
Tel +32 16 34 86 00 Fax +32 16 34 86 01 ec@uzleuven.be ec-submission@uzleuven.be www.uzleuven.be/ec

ONS KENMERK ML9517
UW KENMERK
LEUVEN , 31 juli 2013

De Commissie bevestigt dat in geval van belangenconflict, de betrokken leden niet deelnemen aan de besluitvorming omtrent de studie.

Een ledenlijst wordt bijgevoegd.

De opdrachtgever is verantwoordelijk voor de conformiteit van de anderstalige documenten met de Nederlandstalige documenten.

Aandachtspunten: (indien van toepassing)

Indien er een Clinical Trial Agreement is, kan de studie in ons centrum pas aangevat worden wanneer dit Clinical Trial Agreement goedgekeurd en ondertekend is door de gedelegeerde bestuurder van UZ Leuven.

Studies met geneesmiddelen en sommige studies met "medische hulpmiddelen" dienen door de opdrachtgever aangemeld te worden bij het FAGG.
Studies met geneesmiddelen mogen slechts aanvangen op voorwaarde dat de minister (FAGG) geen bezwaren heeft kenbaar gemaakt binnen de wettelijke termijnen zoals beschreven in art.13 van de Belgische wet van 7/5/2004 inzake experimenten op de menselijke persoon. Voor bepaalde studies met medische hulpmiddelen gelden eveneens wettelijke termijnen (zie KB van 17/3/2009). Voor meer informatie hieromtrent verwijzen we naar de website van het FAGG www.fagg-afmps.be

Onderzoek op embryo's in vitro valt onder de wet van 11 mei 2003. Voor dergelijk onderzoek is er naast een positief advies van het Ethisch Comité ook een goedkeuring van de Federale Commissie voor medisch en wetenschappelijk onderzoek op embryo's in vitro noodzakelijk vooraleer dit onderzoeksproject kan doorgaan.

Gelieve ook rekening te houden met de regelgeving van het ziekenhuis betreffende weefselbeheer en met de beschikkingen van de wet van 19 december 2008.

Dit gunstig advies van de Commissie houdt niet in dat zij de verantwoordelijkheid voor de geplande studie op zich neemt. U blijft hiervoor dus zelf verantwoordelijk. Bovendien dient U er over te waken dat uw mening als betrokken onderzoeker wordt weergegeven in publicaties, rapporten voor de overheid enz., die het resultaat zijn van dit onderzoek.
U wordt eraan herinnerd dat bij klinische studies iedere door U waargenomen ernstige verwikkeling onmiddellijk zowel aan de opdrachtgever (desgevallend de producent) als aan de commissie medische ethiek moet worden gemeld, ook al is het oorzakelijke verband met de studie onduidelijk.
Indien de studie niet binnen het jaar beëindigd is, vereist de ICH-GCP dat een jaarlijks vorderingsrapport aan de commissie wordt bezorgd.
Tenslotte verzoeken wij U ons mee te delen indien een studie niet wordt aangevat, of wanneer ze wordt afgesloten of vroegtijdig onderbroken (met opgave van eventuele reden). Gelieve het (al dan niet vroegtijdig) stopzetten van een studie binnen de door de wet vastgelegde termijnen mee te delen en een Clinical Study Report aan de Commissie te bezorgen.

SECRETARIAAT: **M. LEYS** **N. OPDEKAMP** **D. VAN MOLL** **M. VERBEECK** **H. HUYGHE**
Tel +32 16 34 86 00 Fax +32 16 34 86 01 ec@uzleuven.be ec-submission@uzleuven.be www.uzleuven.be/ec

ONS KENMERK ML9517
UW KENMERK
LEUVEN , 31 juli 2013

Met de meeste hoogachting,

Prof. Dr. Walter VAN DEN BOGAERT
Voorzitter Commissie Medische Ethiek
UZ K.U.LEUVEN

Prof. Dr. W. Van den Bogaert
Voorzitter
Commissie Medische Ethiek van de UZ K.U.Leuven

Cc : **FAGG** (Federaal Agentschap voor Geneesmiddelen en Gezondheidsproducten)
Departement R&D
Eurostation, blok 2
Victor Hortaplein 40, bus 40
B-1060 Brussel

Clinical Trial Center (CTC), UZ Leuven, Campus Gasthuisberg

Lokale Commissie(s):

1- De Commissie heeft rekening gehouden met het advies van de volgende lokale commissie(s). De Commissie gaat er dan ook van uit dat deze centra de studie aanvaarden, tenzij tegenbericht:

Lokale Commissie	Onderzoeker
Dr. K. Magerman<br>Ethische Commissie<br>Jessa Ziekenhuis vzw<br>Stadsomvaart 11<br>3500 Hasselt	Dr. J. Dubois

2- De volgende commissie(s) heeft (hebben) een negatief advies uitgebracht:

Lokale Commissie	Onderzoeker
Niet van toepassing	

3- Van de volgende commissie(s) ontvingen we geen advies:

Lokale Commissie	Onderzoeker
Niet van toepassing	

4- De volgende commissie(s) laat (laten) ons weten dat ze niet in het bezit is (zijn) van het dossier:

Lokale Commissie	Onderzoeker
Niet van toepassing	

COMMISSIE MEDISCHE ETHIEK VAN DE UNIVERSITAIRE ZIEKENHUIZEN K.U.LEUVEN
TOETSINGSCOMMISSIE
UZ GASTHUISBERG E330
HERESTRAAT 49
3000 LEUVEN BELGIUM

KATHOLIEKE UNIVERSITEIT LEUVEN

Ledenlijst Commissie Medische Ethiek/Toetsingscommissie (OG032) vanaf 13 februari 2013 tot op heden
List of Members Ethics Committee/IRB (OG032) from February 13th, 2013 until present

Prof. Walter Van den Bogaert, M.D.	Chairman (M)	Radiotherapy-Oncology
Dr. Johan Wildiers, M.D.	Vice-Chairman (M)	Medical Oncology
Dr. Sabine Graux, M.D.	Secretary (F)	Physician
Dr. Sonja Haesendonck, M.D.	Secretary (F)	Physician
Prof. Xavier Bossuyt, M.D.	Member (M)	Immunology
Prof. D. Bullens, M.D.	Member (F)	Paediatrics
Prof. Willem Daenen, M.D.	Member (M)	Cardiac Surgery
Dr. Lut De Groote, M.D.	External Member (F)	General Practitioner
Prof. Jan de Hoon, M.D.	Member (M)	Clinical Pharmacology
Prof. Ivo De Wever, M.D.	Member (M)	Surgical Oncology
De heer Filip Gybels	Member (M)	Head Nurse
Mrs. Christine Mathieu, Law	Member (F)	Medical Legislation
Dr. José Thomas, M.D.	Member (M)	Medical Oncology
Prof. Josse R. Thomas	Member (M)	Clinical Pharmacology
Prof. Ben Van Calster	Member (M)	Statistics
Prof. Jan Van Hemelrijck, M.D.	Member (M)	Anesthesiology
Prof. Raymond Verhaeghe, M.D.	Member (M)	Cardiology
Prof. Guido Verhoeven, M.D.	Member (M)	Experimental Medicine

(M) = Male (F) = Female

De Commissie voor Medische Ethiek volgt de voorschriften van ICH Good Clinical Practice en de lokale wettelijke bepalingen terzake (wet van 7 mei 2004 inzake experimenten op de menselijke persoon en bijbehorende KB's en programmawet)

The Ethics Committee operates according to ICH Good Clinical Practice and local applicable regulations.

Appendix 3: Risk management analysis

A full risk management analysis was performed based on ISO 14971 *Medical devices — Application of risk management to medical devices* and under guidance of CE-mark expert Mr. Peter Frederickx from consultancy company Think & Do, Klappijstraat 29, B-3294 Diest, Belgium.

All LOGIC-Insulin software items and subitems (i.e. different aspects and features) were analysed individually with respect to the potential hazards and clinical risks that are associated to each of them. The probability of appearance of the hazard, the severity of the hazard and the probability of the hazard detection were determined using an a priori defined coding mechanism and under the assumption that no control measure exists. An overview of the definitions that were set for the software under study is presented in Table A3.1, Table A3.2 and Table A3.3. The product of the score for probability of appearance of the hazard, the score for severity of the hazard and the score for probability of hazard detection is called the Hazard Risk Index (HRI). Items or subitems with hazards that return high HRI values are potentially the most risky and require most attention regarding adequate control measures. Based on the aforementioned definitions the maximum allowable HRI was set at 200. The visual interpretation of HRI is given in Table A3.4.

In a following phase 'control' measures were developed particularly aiming to reduce the probability of hazard appearance and to increase the probability of the hazard detection. In most cases the severity of the hazard could not be adapted. Accordingly, it was our goal to reduce the HRI as much as possible (at least HRI<200) for all items and subitems. The designed control measures involved error messages and warnings for the user as well as safety algorithms (internal, i.e. not visible for the user). Due to the appropriate control measures the recomputation of the HRIs returned values lower than 200 except for one subitem (*Real insulin pump rate differs from flow of LOGIC-Insulin database*, HRI = 336). For this specific scenario the insulin dosage at the pump differs from the insulin rate that is entered in LOGIC-Insulin due to human error (e.g. although confirmed in LOGIC-Insulin, nurse forgets to adapt pump rate).

In current ICU practice no control measure can be implemented to cover this hazard. However, in the near future, it will be able to 'push' the effective pump rates (nutrition, insulin and other 'drip' medication) towards the PDMS system. Automatic inclusion of the data from the PDMS system to LOGIC-Insulin will allow to lower the workload for the nurses and to know the exact pump rates. While awaiting the availability of such automatic data transfer this exceptional 'higher than 200' HRI value should be tolerated. Nurses will be instructed to precisely adapt the infusion pump rates as considered in LOGIC-Insulin and the PDMS. Unlike most other drugs, the effect of insulin is monitored as an *open system* allowing to observe the 'output' (glycaemia) on a regular base and to intervene if needed. The 'inputs' (among the insulin flow) will be investigated in case of unexpected deviations from the target glucose range. Possible deviations due to discrepancies between the LOGIC-Insulin flow rate and the effective pump rate will be detected. In addition, during the clinical trial specifically

trained study nurses will control the correct application of the study protocol and will regularly check (and eventually correct) data input (e.g. agreement between database entries and real-life device readings/settings, among pump rates).

We want to note that for each hazard detected in the risk management analysis a test scenario was written, executed and, finally, reported in an issue tracking database. The full risk management analysis for the LOGIC-Insulin software is only available in electronic format due to its complex and extensive design (no printable format).

**Probability of hazard appearance**			
	**Frequency per year for 1 ICU bed (365 ICU days)**	**Interpretation: chance of appearance of a fail**	**SCORE**
**Frequent, high chance (> once per day)**	> 365 (more than once per day)	Not surprised, will occur several times	**10**
**Probable (> once per month)**	12-365 (more than once per month)	Occurs repeatedly / event to be expected	**8**
**Occasional (> once per year)**	1-12 (more than once per year)	Could occur some time	**6**
**Remote (> once per 2 years)**	0.5-1 (more than once per 2 years)	Unlikely though conceivable	**4**
**Improbable (>once per 10 years)**	0.1-0.5 (more than once per 10 years)	So unlikely that probability is close to zero	**2**
**Zero chance (no chance)**	0 (no chance)	Probability is zero	**0**

Table A3.1. Probability of hazard appearance

Severity of the hazard		
	**Importance of the consequence of failure**	**SCORE**
**Catastrophic**	Potential of multiple deaths	**10**
	Potential of multiple severe injury	**9**
**Critical**	Potential of single (patient) death	**8**
	Potential of single (patient) severe injury or pain	**7**
	Potential of single (patient) injury or pain	**6**
**Uncomfortable**	Potential of multiple serious discomfort	**5**
	Potential of single (patient) serious discomfort	**4**
	Potential of multiple discomfort	**3**
	Potential of single (patient) discomfort	**2**
	Very small hazard	**1**
**No consequence**	No hazard	**0**

Table A3.2. Severity of the hazard

Probability of hazard detection		
	**Chance of detecting the hazard before delivering and appropriate action**	**SCORE**
**Low detection chance**	Hazard will never be detected	**10**
	Hazard can only be detected by serious LOGIC-Insulin ADMIN investigation (extra queries, database check, log files)	**9**
	Hazard can only be detected by serious hospital ADMIN investigation	**8**
**Medium detection chance**	Hazard can only be detected by hospital ADMIN investigation (regular checks)	**7**
	Hazard can only be detected by serious user investigation	**6**
	Hazard will be detected during extended use	**5**
**High detection chance**	Hazard will be detected during normal use	**4**
	Hazard will directly appear when device is turned on	**3**
	Hazard will be detected by visual check	**2**
	Hazard will always be detected	**1**
	No hazard	**0**

Table A3.3. Probability of hazard detection

		Probability of hazard appearance					
		Frequent, high chance (> once per day)	Probable (> once per month)	Occasional (> once per year)	Remote (> once per 2 years)	Improbable (>once per 10 years)	Zero chance (no chance)
Severity of the hazard	Potential of multiple deaths	1000	800	600	400	200	0
	Potential of multiple severe injury	900	720	540	360	180	0
	Potential of single (patient) death	800	640	480	320	160	0
	Potential of single (patient) severe injury or pain	700	560	420	280	140	0
	Potential of single (patient) injury or pain	600	480	360	240	120	0
	Potential of multiple serious discomfort	500	400	300	200	100	0
	Potential of single (patient) serious discomfort	400	320	240	160	80	0
	Potential of multiple discomfort	300	240	180	120	60	0
	Potential of single (patient) discomfort	200	160	120	80	40	0
	Very small hazard	100	80	60	40	20	0
	No hazard	0	0	0	0	0	0

Table A3.4. Representation of the Hazard Risk Index (HRI) assuming that the probability of hazard detection gets the highest score (10: *Hazard will never be detected*) indicating a worst case scenario. The maximum allowable HRI for the software under study was set at 200.

Appendix 4: Incorporation of human blood

We here declare that the medical device under study does not incorporate, as an integral part, a substance or human blood derivative.

07-01-2014

D. MESOTTEN

Prof. Dr. Dieter Mesotten

Signature Clinical Investigator

Appendix 5: Dutch Manual

Only a Dutch manual is currently available as all nurses of the participating ICUs speak and understand the Dutch language (mother tongue). The LOGIC-Insulin manual is included in the following pages.

LOGIC-Insulin

LOva (Leuven) Glucose control for Intensive Care

Tom Van Herpe

`<tom.vanherpe@esat.kuleuven.be>`

Guy Veraghtert

`<guy.veraghtert@esat.kuleuven.be>`

Revision 9517

2013-11-21

Table of Contents

.... iv
1. Wanneer te gebruiken? 1
2. Wanneer niet gebruiken? 2
3. Aanmelden 3
4. Selectie van een patiënt 4
    4.1. Nieuwe opname 4
    4.2. Opzoeken van een bestaande patiënt 6
5. Ingeven van een nieuwe consultatie 9
    5.1. Ingeven van de voeding 11
6. Ingeven van de toegediende insuline 14
7. Wachtscherm 16
    7.1. Ingeven van nieuwe bloedglucose 17
    7.2. Wijzigen van de voeding 18
    7.3. Pauze 18
    7.4. Stoppen 20
8. Afmelden 22
9. Praktische situaties 23
Glossarium 25

List of Figures

3.1. *Log in* scherm 3
4.1. *Patiënt-overzicht* scherm 4
4.2. *Patiënt-gegevens* scherm met foutieve waarden 5
4.3. Correct ingevuld *Patiënt-gegevens* scherm 6
4.4. *Patiënt-overzicht* scherm: Zoek een bestaande patiënt 7
4.5. *Wacht* scherm: Patiënt openen op andere computer 8
5.1. Initieel *Consultatie* scherm 10
5.2. Ingevuld *Consultatie* scherm 11
5.3. Initieel *Voeding* scherm 12
5.4. Ingevuld *Voeding* scherm 13
6.1. *Advies* scherm: Bevestiging van voorgesteld advies 14
6.2. Afwijking van voorgesteld advies 15
7.1. *Wacht* scherm 16
7.2. *Wacht* scherm: Historiek 17
7.3. *Wacht* scherm: Nieuwe meting 17
7.4. *Wacht* scherm: Wijzig voeding 18
7.5. *Wacht* scherm: Pauze 19
7.6. *Wacht* scherm: Terug op kamer 19
7.7. *Wacht* scherm: Terug op kamer bevestigen 20
7.8. *Wacht* scherm: Stop 20
7.9. *Wacht* scherm: Bevestiging stop LOGIC-Insulin behandeling 21
7.10. *Wacht* scherm: Behandeling met LOGIC-Insulin werd gestopt 21
8.1. Afmelden 22

Chapter 1. Wanneer te gebruiken?

Advies over de toe te dienen insuline aan patiënten op een intensieve eenheid, met als doel:

- Snel naar de doel-"normale"-glycemie
- Vermijden van hypo – en hyperglycemie
- Vermijden van hoge glycemievariabiliteit
- Beperken van verhoging werkdruk

> De software geeft een advies over de toe te dienen insuline (of glucosebolus in geval van hypoglycemie) en het tijdstip van bloedstaalname. De software heeft een ADVISERENDE functie. Het toedienen van insuline mag enkel gebeuren door gekwalificeerd personeel die de risico's eigen aan strikte bloedglucosecontrole kan inschatten.

Chapter 2. Wanneer niet gebruiken?

- De patiënt is jonger dan 18 jaar
- Er is geen arteriële lijn (meer) beschikbaar
- De patiënt begint "normaal" te eten en/of drinkt calorierijke dranken (bv. boterham, pudding, fruitsap)
- Er is geen indicatie (meer) voor (strikte) glycemiecontrole (bv. therapiestop, palliatieve zorg)
- De geobserveerde hyper- of hypoglycemie lijkt niet behandelbaar (*refractory* hyper- of hypoglycemie)
- De patiënt is zwanger of geeft borstvoeding
- De patiënt heeft een allergie voor insuline
- De patiënt lijdt aan keto-acidose of hyperosmolair coma (bij opname)
- Medisch personeel heeft onvoldoende ervaring met (strikte) glycemiecontrole

Chapter 3. Aanmelden

Alvorens men de software kan gebruiken, dient met in te loggen aan de hand van een Gebruikersnaam/Wachtwoord-combinatie.

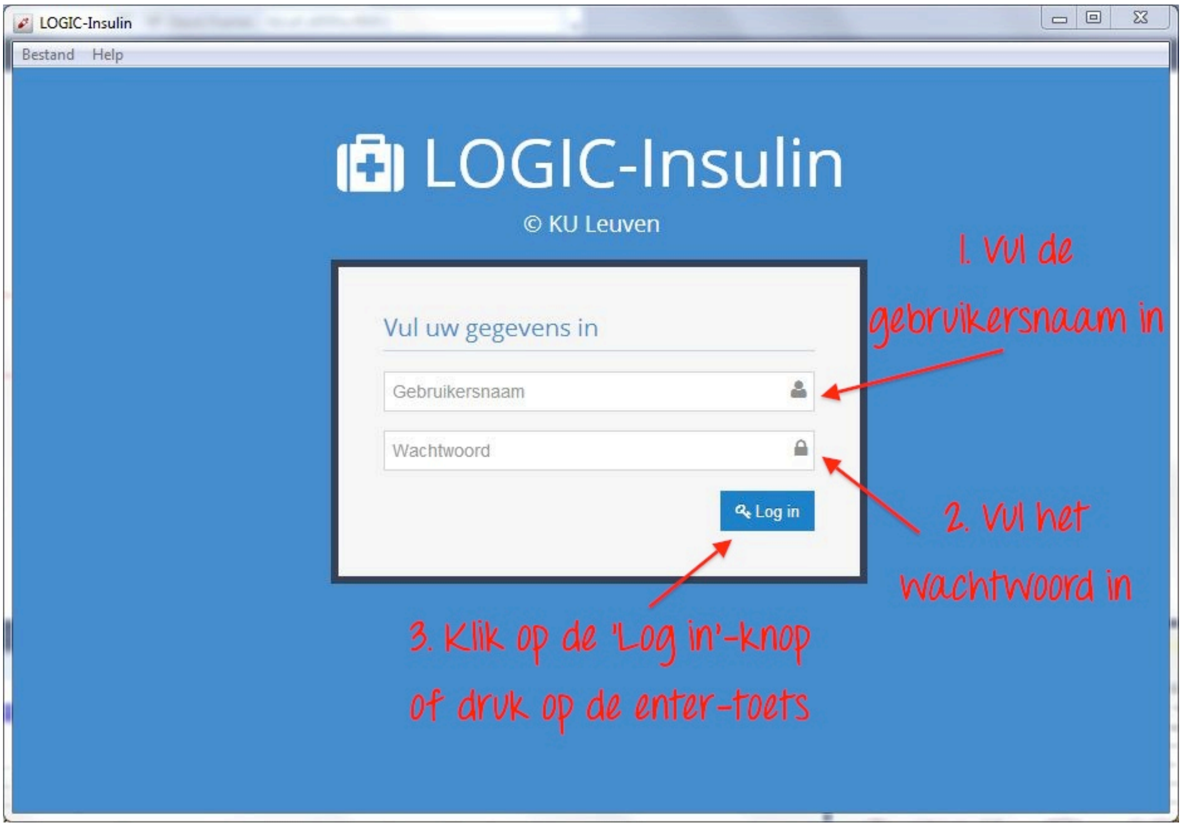

Figure 3.1. *Log in* scherm

Chapter 4. Selectie van een patiënt

De eerste maal na het inloggen, moet de patiënt geselecteerd worden bij wie men de LOGIC-Insulin software wenst te gebruiken. Dit kan men doen door:

- een nieuwe patiënt in te geven
- een patiënt die reeds ingegeven is, op te zoeken (bv. na verhuis van de patiënt naar een ander bed)

Door deze selectie beschouwt de LOGIC-Insulin software de patiënt als gekoppeld aan de computer op de welke men de software gebruikt. Vanaf nu zal de software automatisch het Chapter 7, *Wachtscherm* tonen van deze gekoppelde patiënt bij het starten van de software.

4.1. Nieuwe opname

Wanneer men de LOGIC-Insulin software wenst te gebruiken bij een nieuwe patiënt moet deze eerst ingegeven worden. Dit doet men door op het *Patiënt-overzicht* scherm op de knop *Nieuwe opname* te klikken.

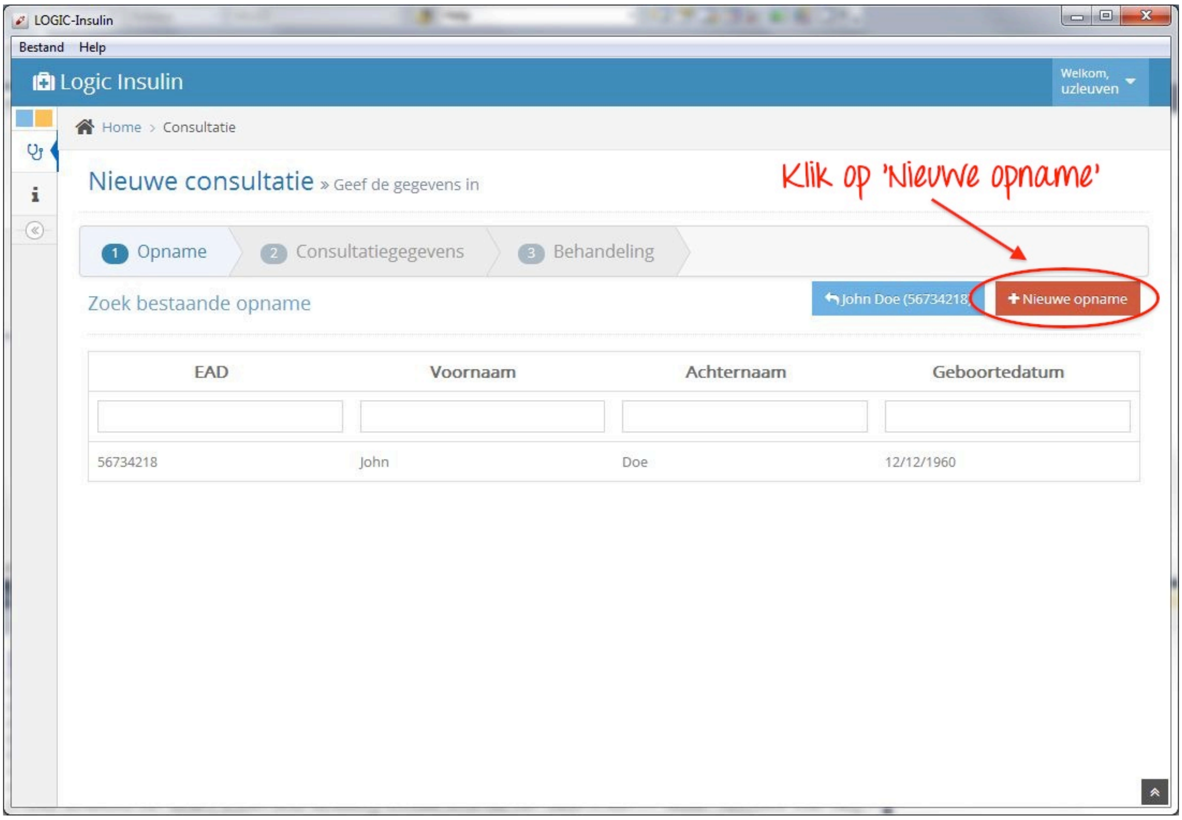

Figure 4.1. *Patiënt-overzicht* scherm

Vervolgens krijgt men het *Patiënt-gegevens* scherm te zien. Hier dient men alle velden die rood zijn (en/of een rood kruisje bevatten) in te vullen. Een foutboodschap wordt getoond op het moment dat men een foutieve of onvolledige waarde intikt. Wanneer een correcte waarde is ingevuld, verdwijnt de rode kleur (en het rode kruisje). Men kan niet op *Opslaan* klikken, zolang er fouten zijn.

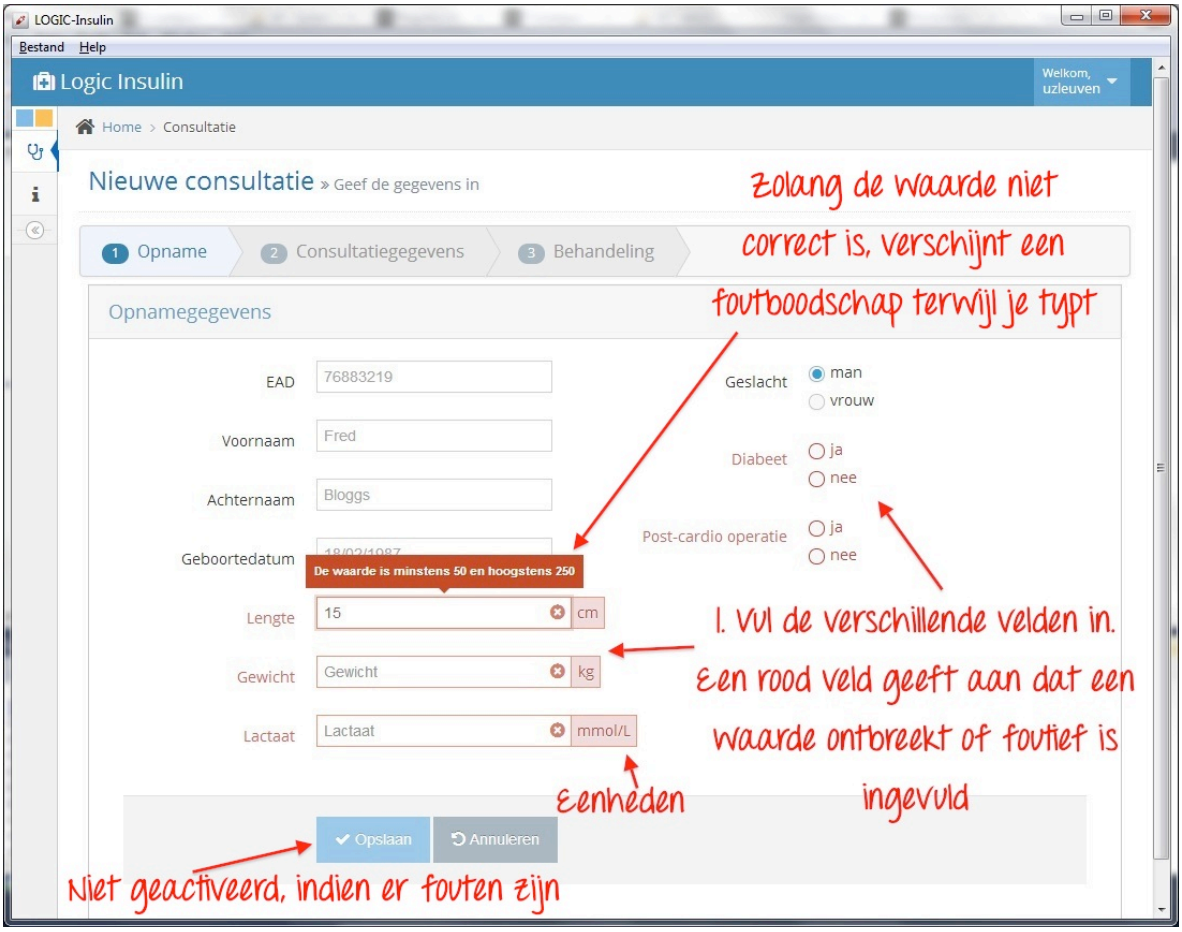

Figure 4.2. *Patiënt-gegevens* scherm met foutieve waarden

Wanneer alle verplichte velden correct zijn ingevuld, wordt de *Opslaan*-knop geactiveerd. Deze knop leidt ons naar het *Nieuwe consultatie* scherm.

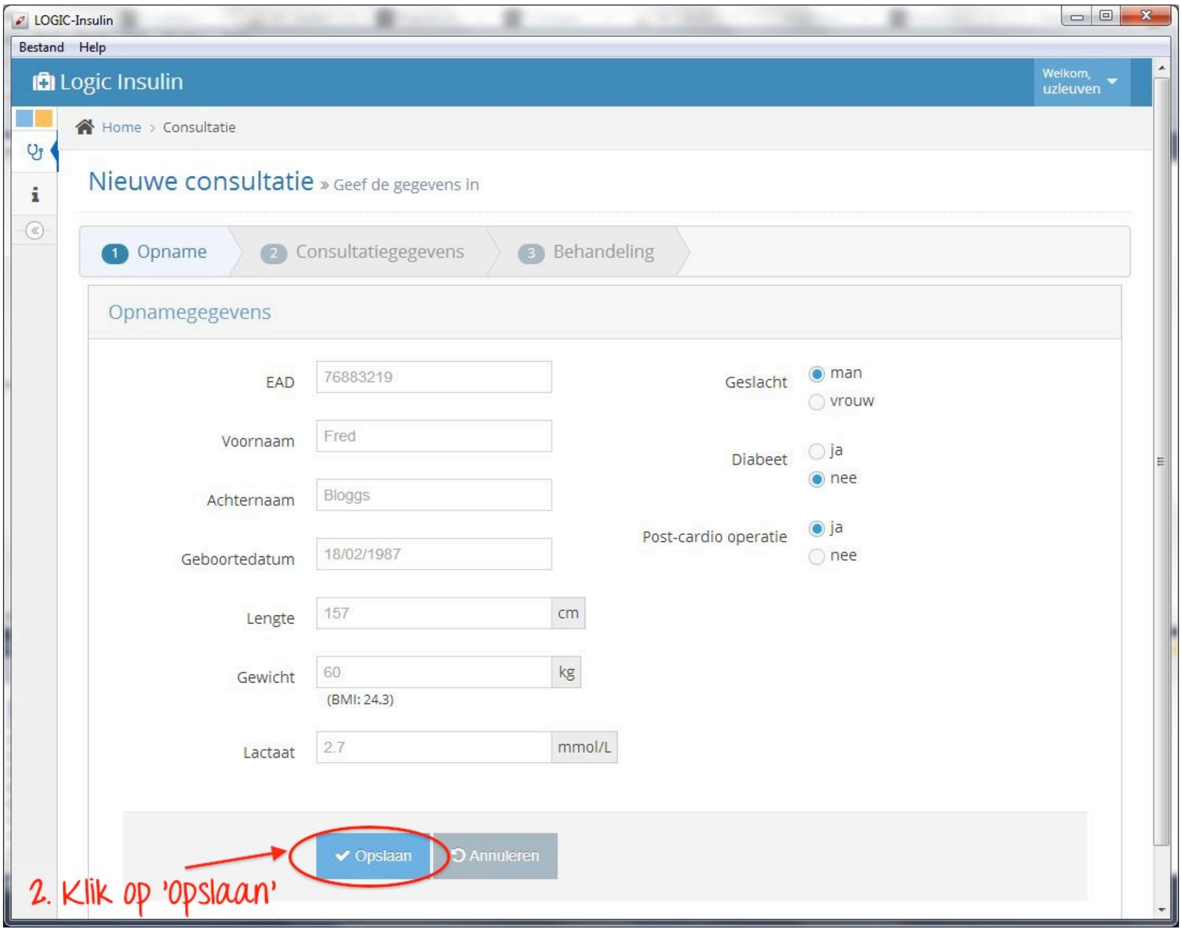

Figure 4.3. Correct ingevuld *Patiënt-gegevens* scherm

4.2. Opzoeken van een bestaande patiënt

Als een patiënt bij wie de LOGIC-Insulin software gebruikt wordt, van bed/kamer verhuist, is het NIET nodig om de gegevens van deze patiënt opnieuw in te geven op de nieuwe computer. Via het *Patiënt-overzicht* scherm, kan men een reeds ingegeven patiënt opzoeken en selecteren. Men krijgt dan het *Wacht* scherm van deze patiënt.

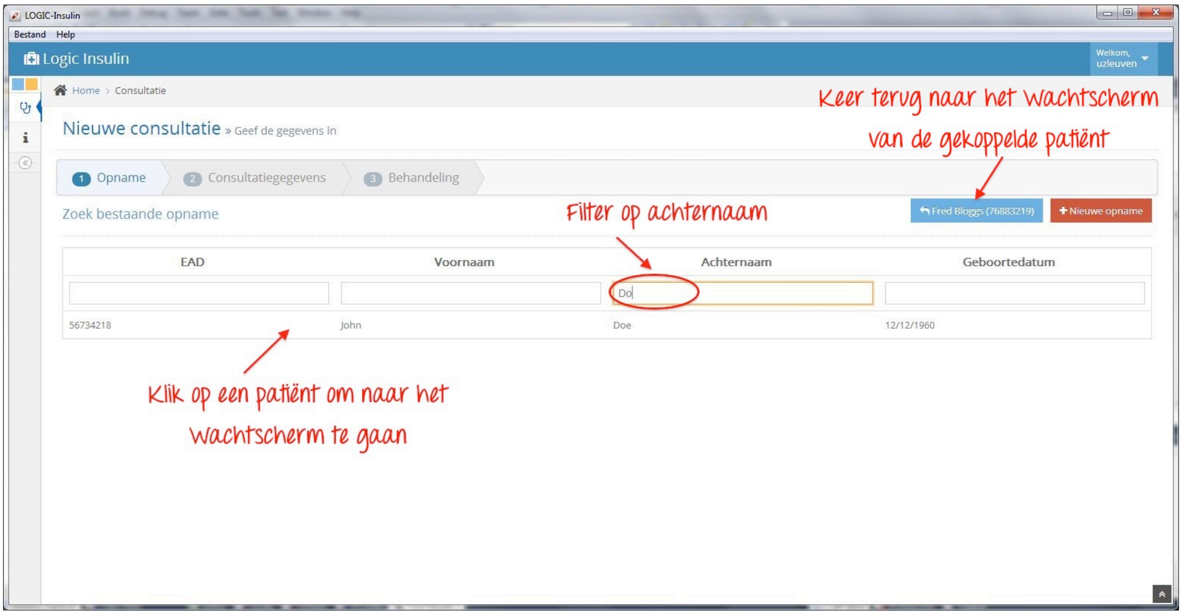

Figure 4.4. *Patiënt-overzicht* scherm: Zoek een bestaande patiënt

Vanuit het Chapter 7, *Wachtscherm* kan men steeds naar het *Patiënt-overzicht* scherm navigeren door op het *stethoscoop*-symbool te klikken

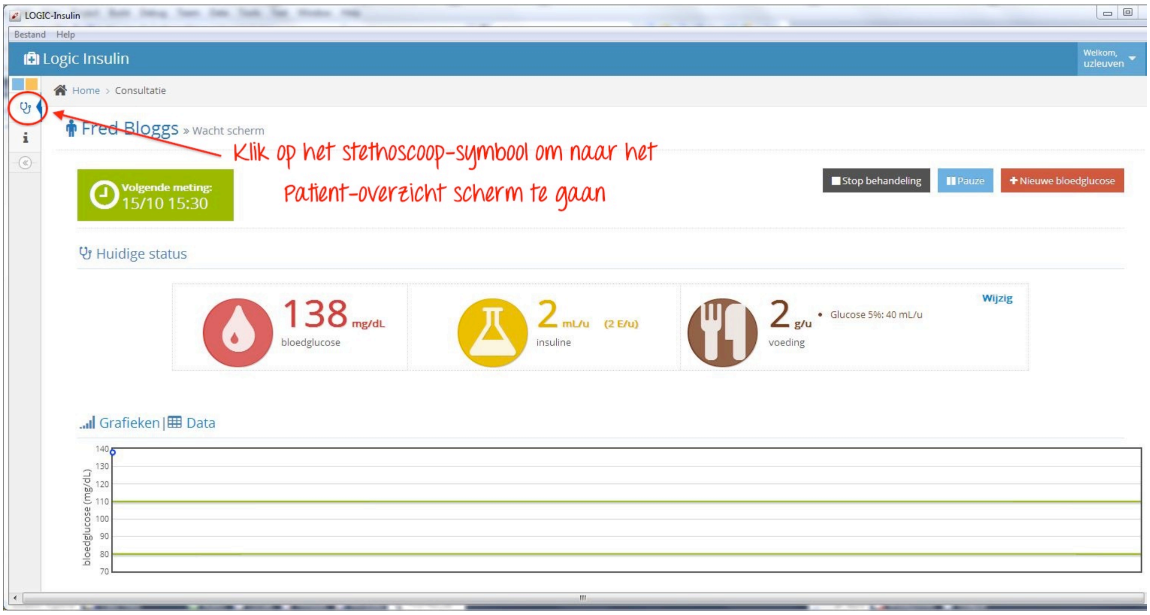

Aangezien deze patiënt nog niet aan die nieuwe computer gekoppeld is, verschijnt een rode balk. Deze rode balk maakt de gebruiker attentief opdat vermeden zou worden dat een bloedglucose bij de verkeerde patiënt wordt ingegeven.

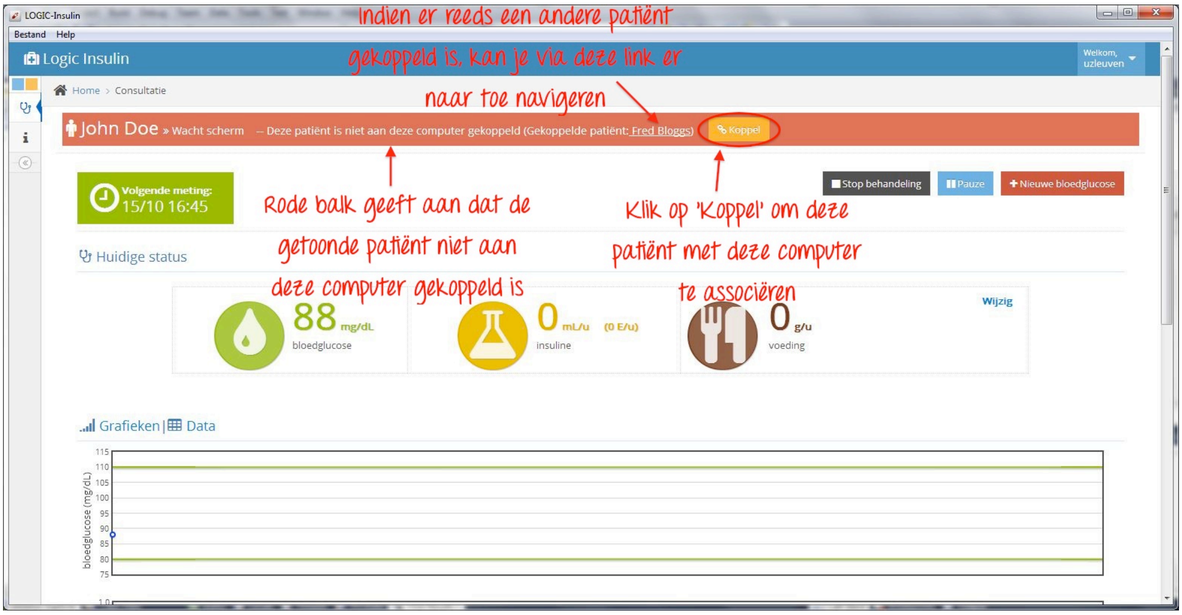

Figure 4.5. ***Wacht*** **scherm: Patiënt openen op andere computer**

Chapter 5. Ingeven van een nieuwe consultatie

Na het invullen en opslaan van de patiëntgegevens bij Opname van een nieuwe patiënt of na het klikken op *Nieuwe bloedglucose* in het *Wacht* scherm bij een bestaande patiënt opent het *Consultatie* scherm waarin volgende velden verplicht ingegeven moeten worden:

- Bloedglucose: de glycemie bekomen via een **arterieel** genomen bloedstaal (*veneuze* bloedstalen kunnen resulteren in onnauwkeurige glycemiebepalingen en worden daarom NIET gebruikt in LOGIC-Insulin)
- Insulineconcentratie: de concentratie aan insuline in de pomp
- Voeding veranderd: indien de patiënt enterale of parenterale voeding krijgt, klikt men op *ja* en krijgt men het *Voeding* scherm te zien. Zoniet klikt men op *nee*.

Indien de patiënt recent een corticosteroïd **bolus** heeft gekregen (minder dan twee uur geleden) of binnen de 15 minuten zal krijgen, dient deze waarde ook ingevuld te worden. Dosissen "per os" of "drip" moeten NIET ingevuld worden.

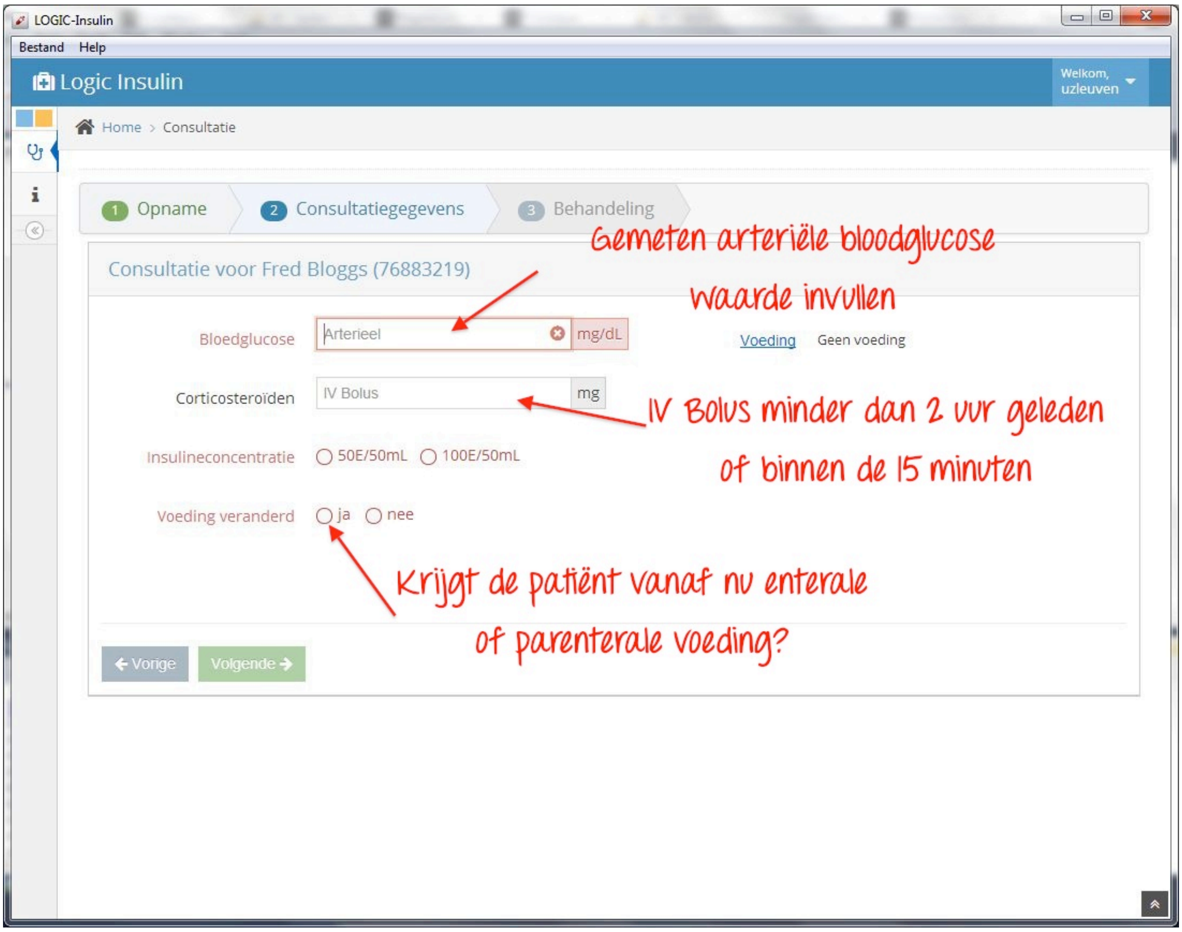

Figure 5.1. Initieel *Consultatie* scherm

Wanneer alle waarden zijn ingegeven, klikt men op *Volgende* en komt men op het *Advies* scherm.

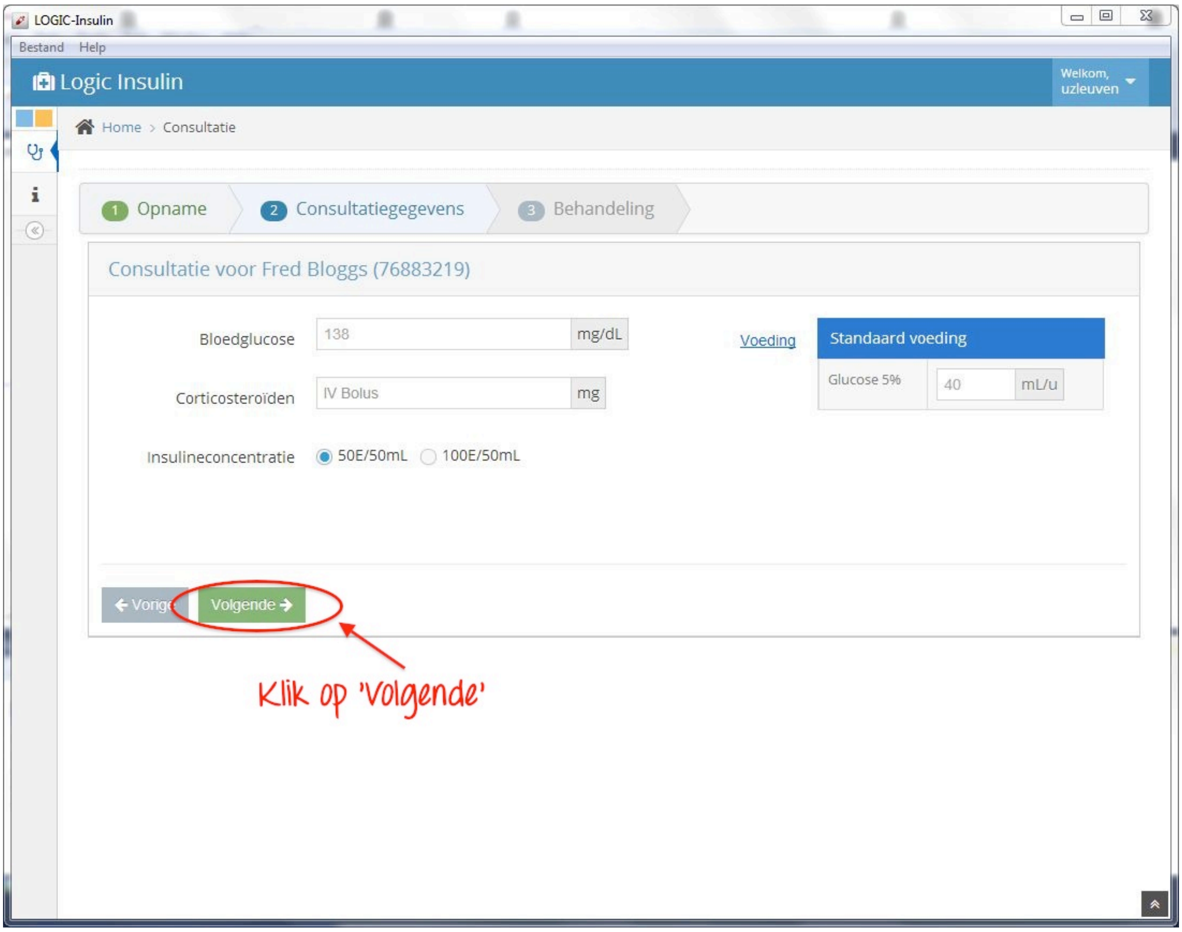

Figure 5.2. Ingevuld *Consultatie* scherm

5.1. Ingeven van de voeding

In het *Voeding* scherm kan men één of meerdere voedingen registreren die de patiënt gedurende de volgende periode toegediend krijgt. Hiervoor vult men het debiet van de voedingspomp in en selecteert men het type voeding uit een lijst. Vervolgens klikt men op de *Voeg toe* knop.

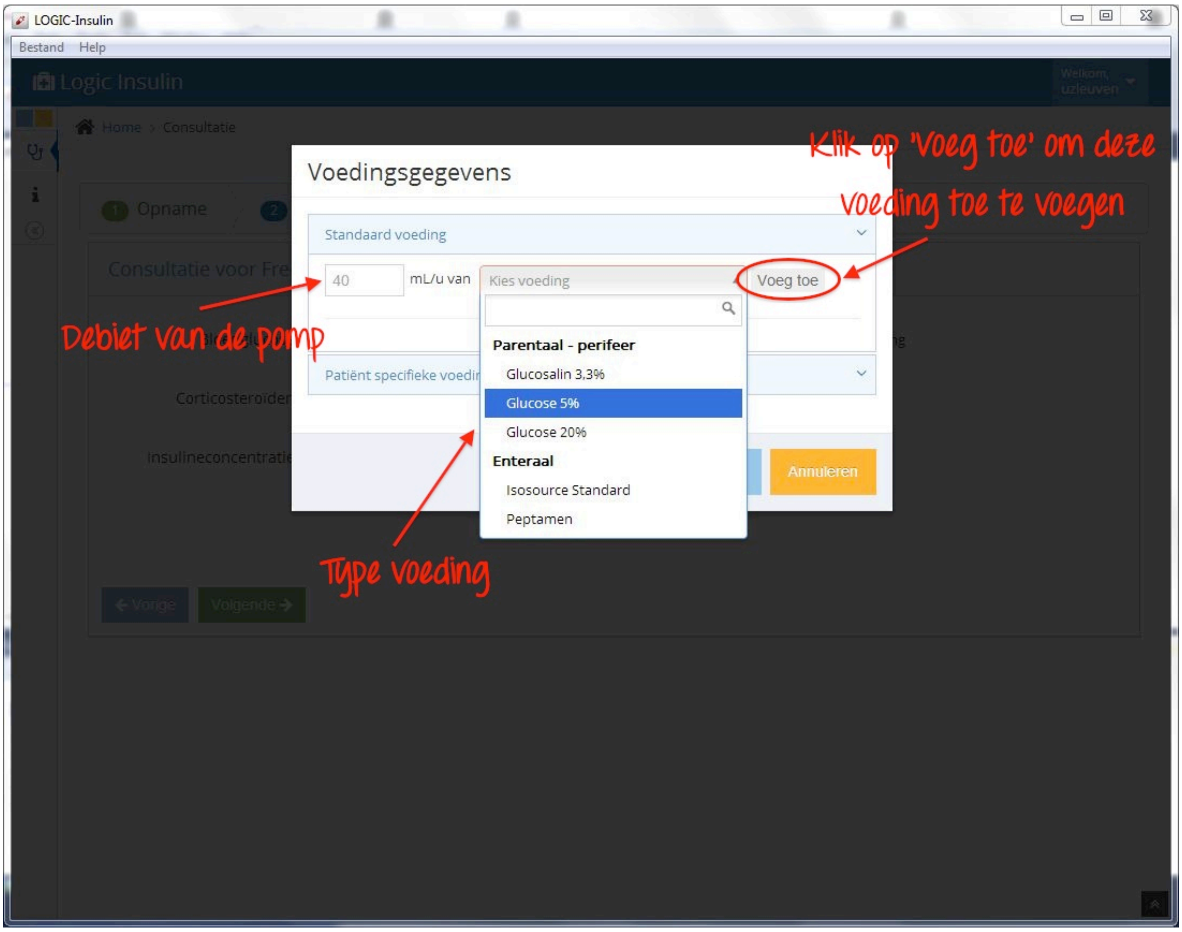

Figure 5.3. Initieel *Voeding* scherm

Wanneer alle voedingen zijn toegevoegd, klikt men op *Opslaan*.

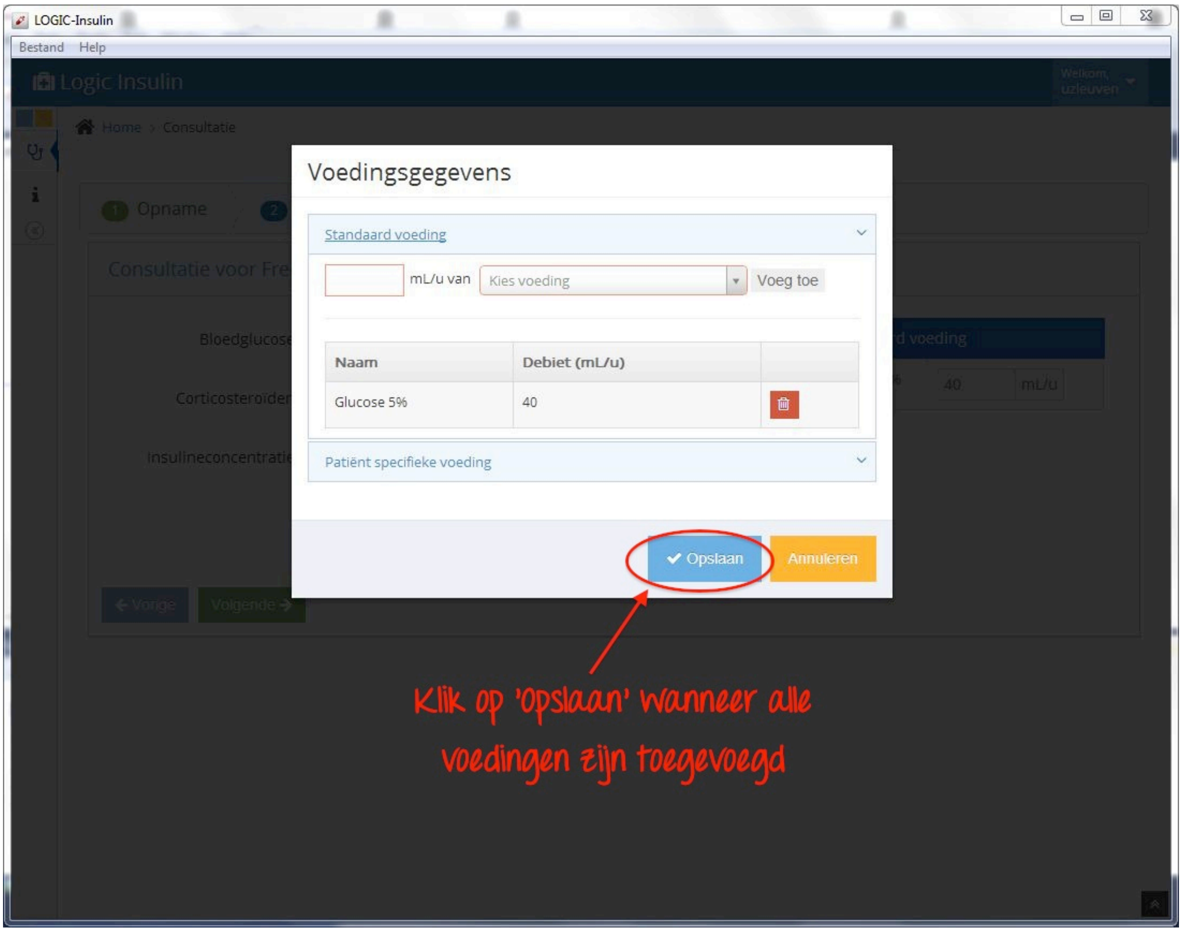

Figure 5.4. Ingevuld *Voeding* scherm

Chapter 6. Ingeven van de toegediende insuline

Op het *Advies* scherm stelt de LOGIC-Insulin software de **insulinedosis** voor (debiet voor insulinepomp gedurende de volgende tijdsperiode). In geval van (gemeten of verwachte) hypoglycemie wordt een **glucosebolus** voorgesteld.

Het medisch personeel kan deze geadviseerde waarde bevestigen of corrigeren.

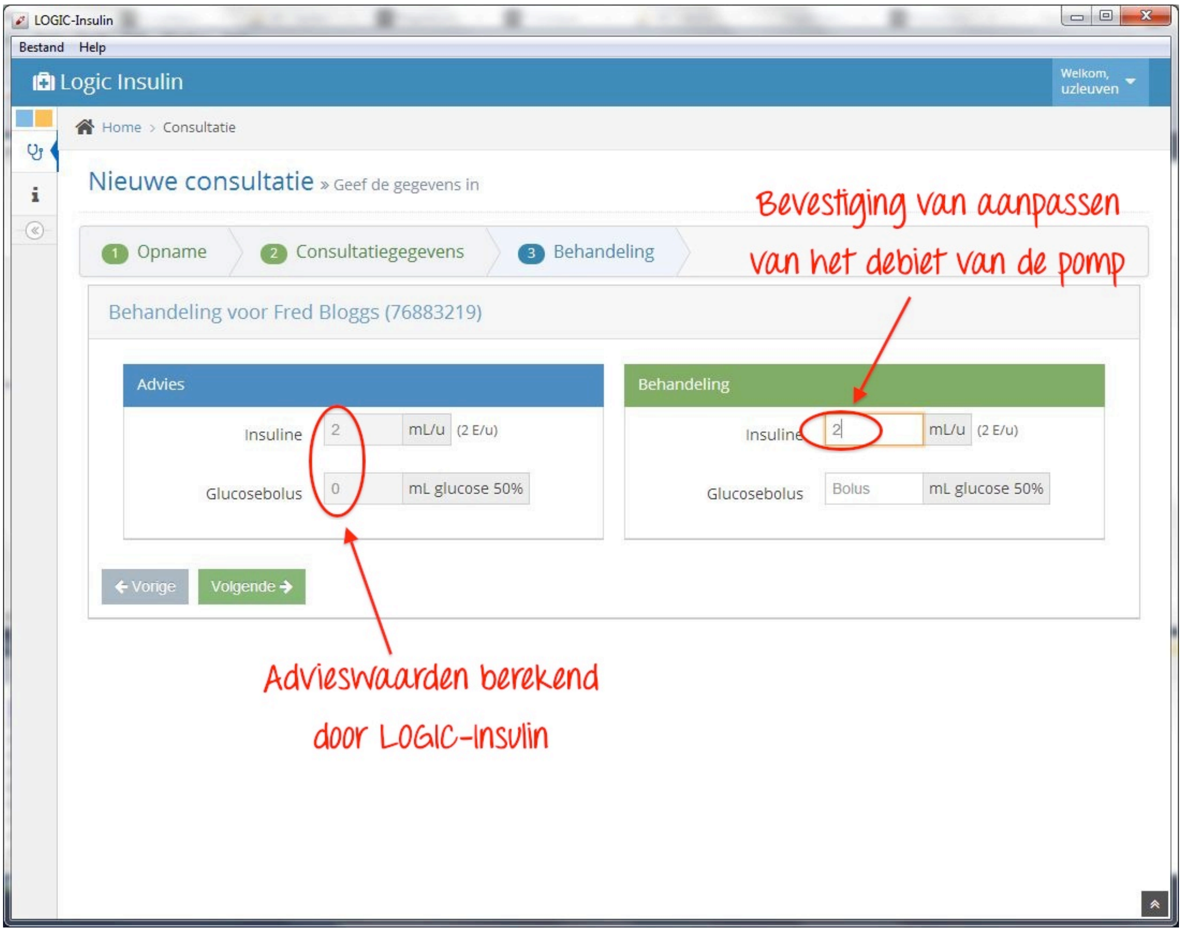

Figure 6.1. *Advies* scherm: Bevestiging van voorgesteld advies

Indien het medisch personeel het advies **niet** opvolgt, verschijnt er een extra scherm waarin de reden van afwijking moet worden ingevuld.

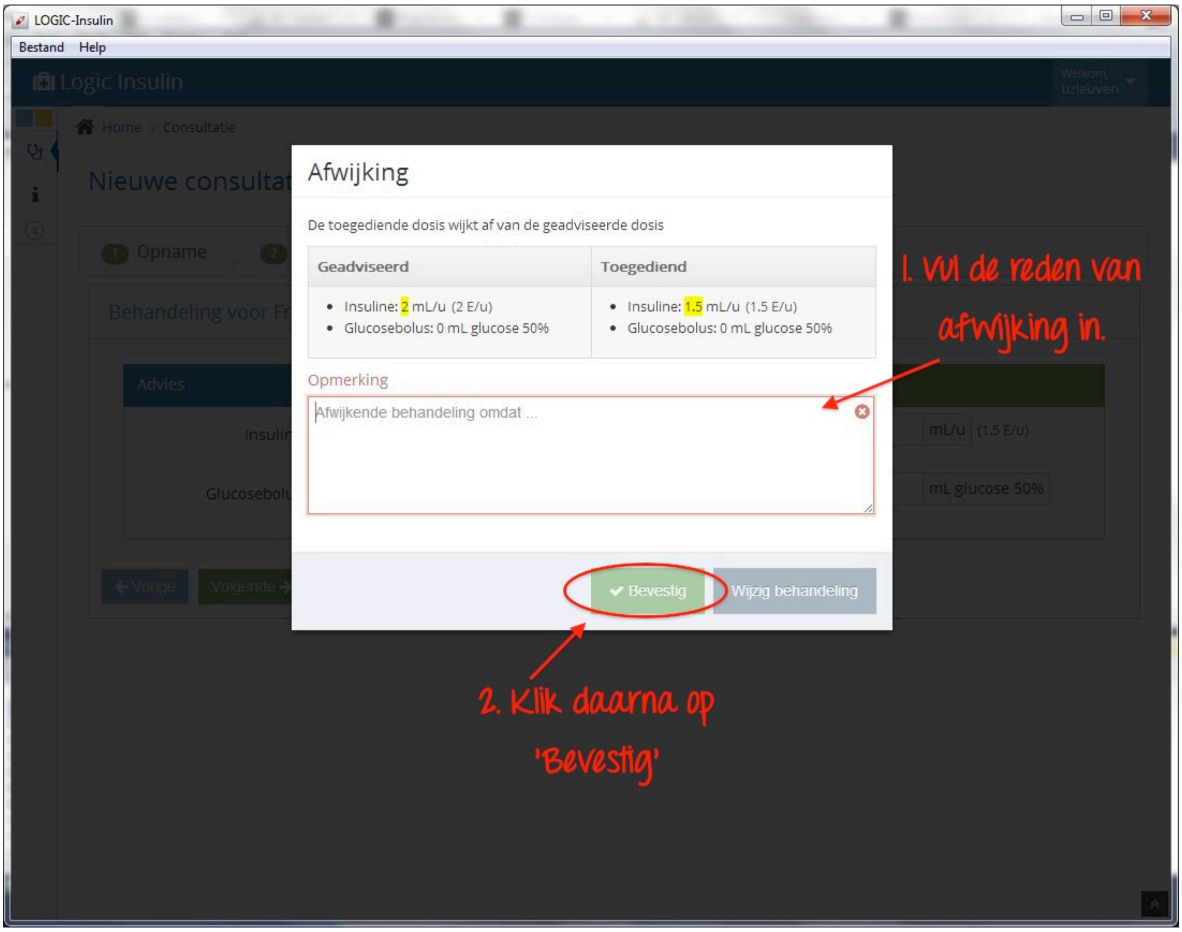

Figure 6.2. Afwijking van voorgesteld advies

Na dit *Advies* scherm komt men op het *Wacht* scherm.

Chapter 7. Wachtscherm

Op het *Wacht* scherm ziet men de huidige situatie van de patiënt:

- De laatst gemeten bloedglucose
- Het huidig debiet van de insulinepomp
- De voeding die de patiënt momenteel toegediend krijgt

Verder ziet men ook de **adviestijd** rond dewelke de LOGIC-Insulin software een nieuwe consultatie (nieuwe bloedstaalname) verwacht.

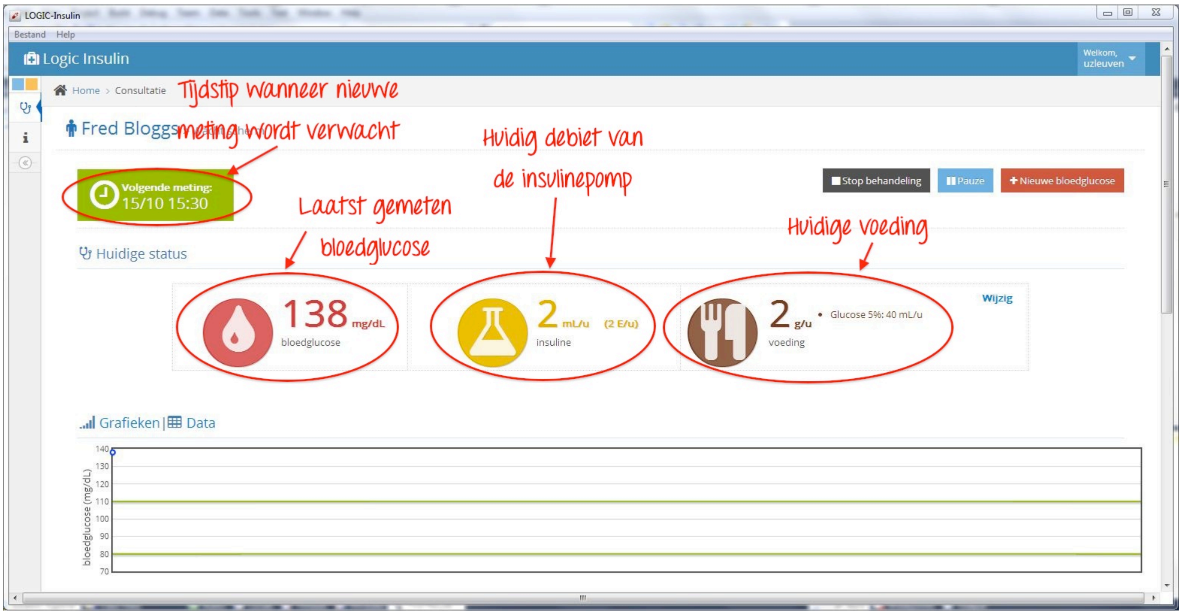

Figure 7.1. *Wacht* scherm

Door op *Grafieken* of *Data* te klikken, kan men de historiek van de patiënt bekijken op een grafische of numerieke manier.

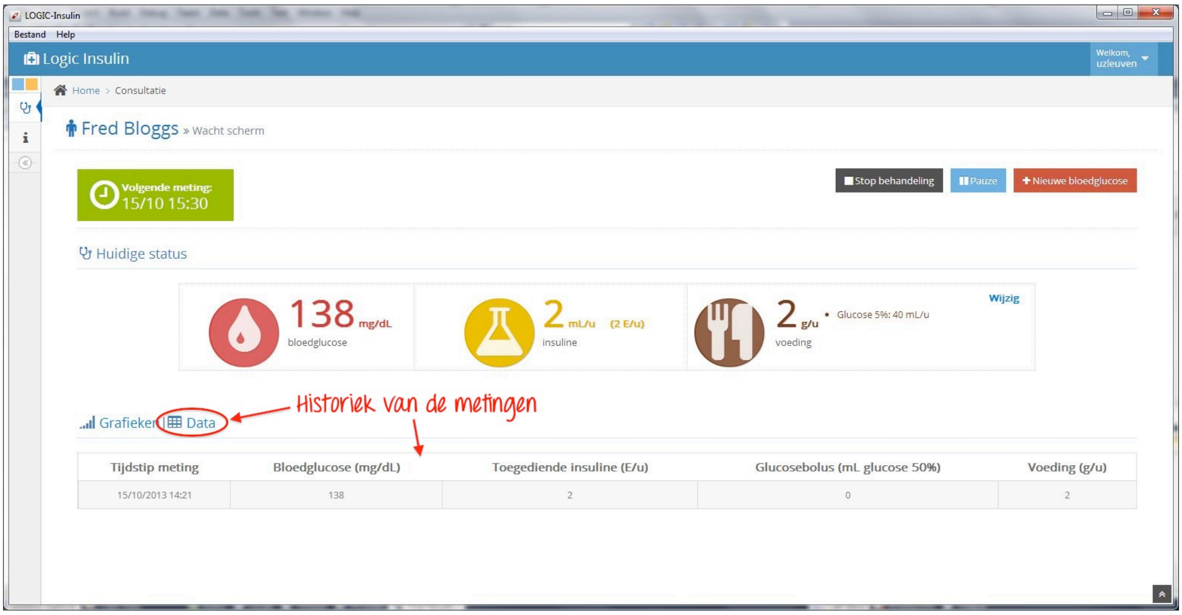

Figure 7.2. *Wacht* scherm: Historiek

7.1. Ingeven van nieuwe bloedglucose

Wanneer een nieuwe meting werd uitgevoerd omstreeks het gevraagde tijdstip (of *vroeger* omwille van een andere reden dan de bloedglucose), klikt men op de knop *Nieuwe bloedglucose*.

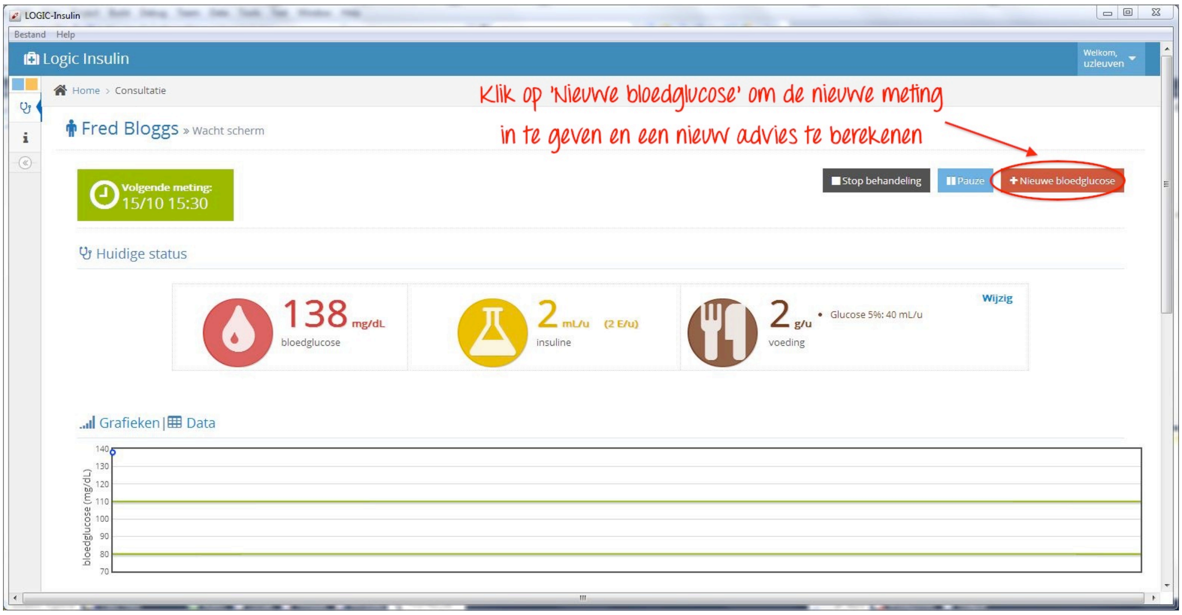

Figure 7.3. *Wacht* scherm: Nieuwe meting

Het *Consultatie* scherm wordt nu weer getoond.

7.2. Wijzigen van de voeding

Wanneer de voeding wordt aangepast zonder dat men een nieuwe meting heeft gedaan, kan dit rechtstreeks gebeuren vanuit het *Wacht* scherm. Klik op *Wijzig voeding* en het *Voeding* scherm wordt getoond.

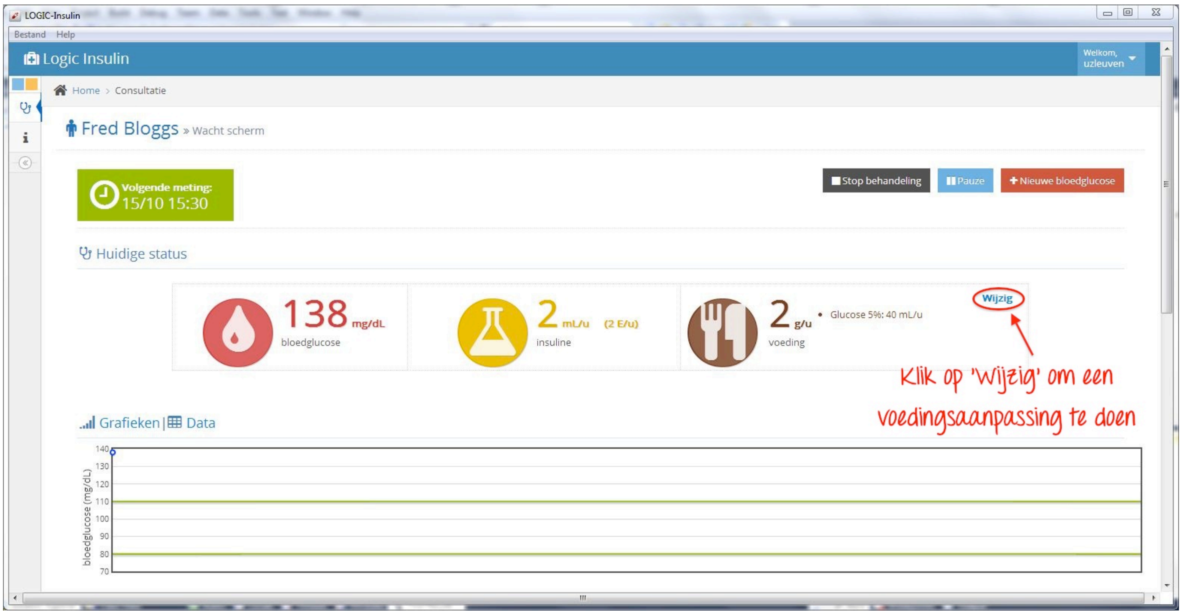

Figure 7.4. *Wacht* scherm: Wijzig voeding

De LOGIC-Insulin software tracht nu de bloedglucose zelf te schatten om vervolgens met deze *virtuele* waarde de insulinedosis te bepalen. Indien dit echter niet met voldoende zekerheid kan gebeuren, zal de software alsnog om een nieuwe meting vragen via het *Consultatie* scherm.

7.3. Pauze

Druk in het *Wacht* scherm op *Pauze* om een tijdelijke stop van insuline/voeding aan te geven (bv. patiënt naar scan, diagnostisch onderzoek, operatie).

> Het speelt **geen** rol of de *Pauze* knop werd geactiveerd vóór, tijdens of na de pauze! M.a.w. indien de pauze voorbij is (bv. patiënt is terug van scan) en de insuline/voeding werden teruggekoppeld aan de patiënt, kan je nog steeds op de *Pauze* knop drukken. **Het is belangrijk dat je de software meegeeft dat er een pauze is geweest.**

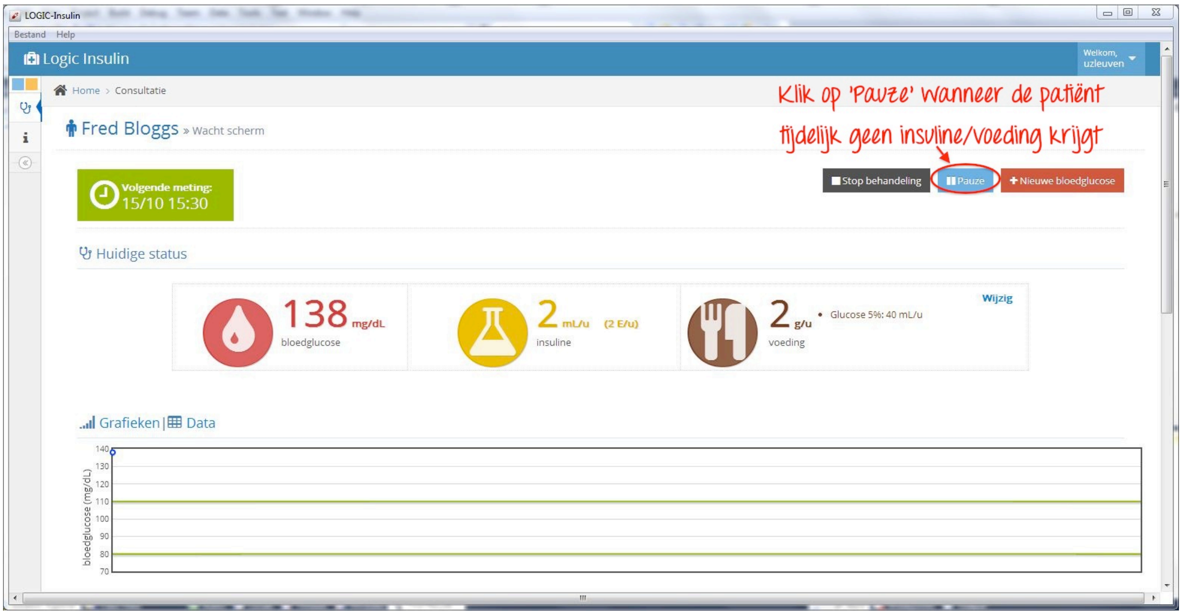

Figure 7.5. *Wacht* scherm: Pauze

Wanneer de patiënt terug op de kamer is, klik je op de knop *Terug op kamer*.

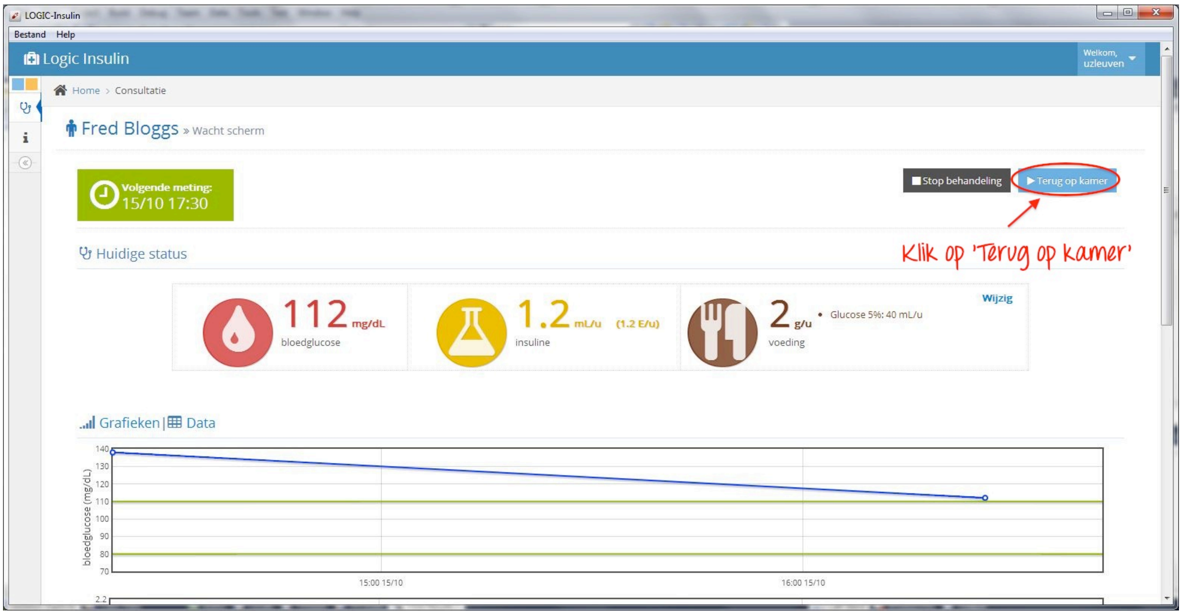

Figure 7.6. *Wacht* scherm: Terug op kamer

De software vraagt nu hoelang de insuline/voeding terug gekoppeld zijn aan de patiënt. Anders gesteld: *Hoelang krijgt de patiënt terug insuline/voeding NA de pauze?* Kies een waarde en bevestig.

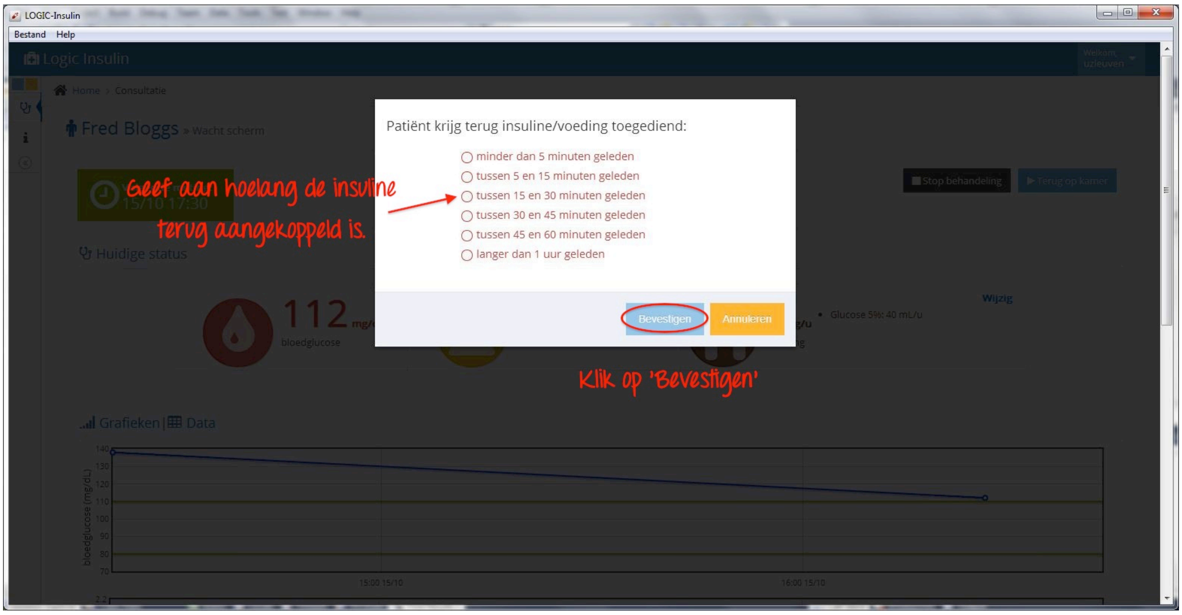

Figure 7.7. *Wacht* scherm: Terug op kamer bevestigen

7.4. Stoppen

Indien de LOGIC-Insulin software niet langer gebruikt moet worden bij een bepaalde patiënt, klik je in het *Wacht* scherm van die patiënt op de knop *Stop behandeling*.

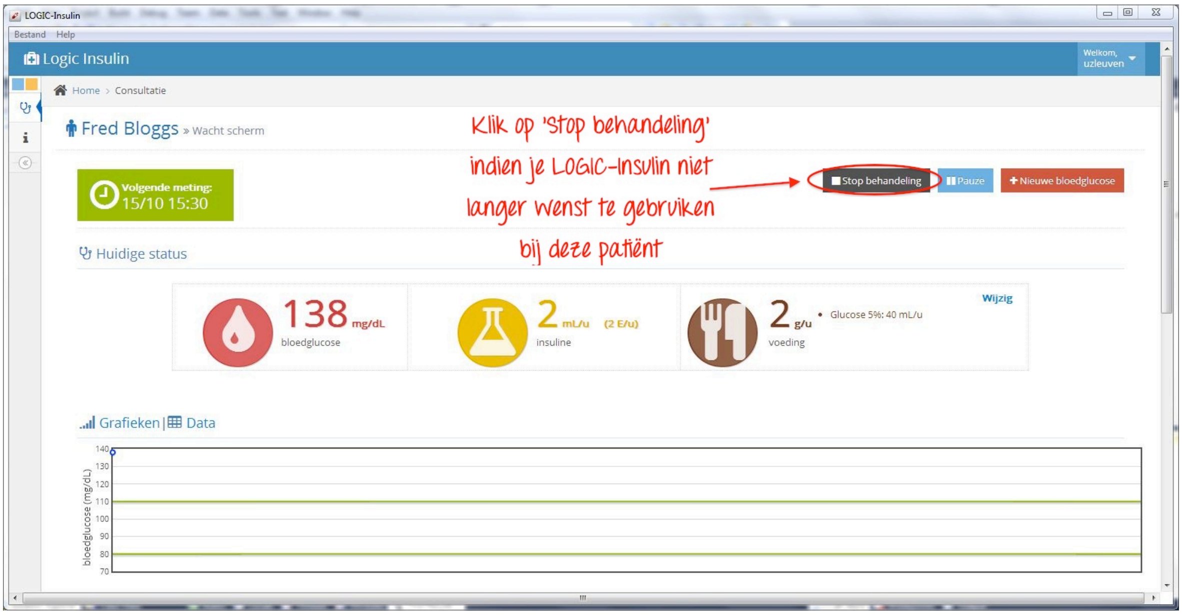

Figure 7.8. *Wacht* scherm: Stop

De software vraagt nu om een reden op te geven. Kies een reden en bevestig.

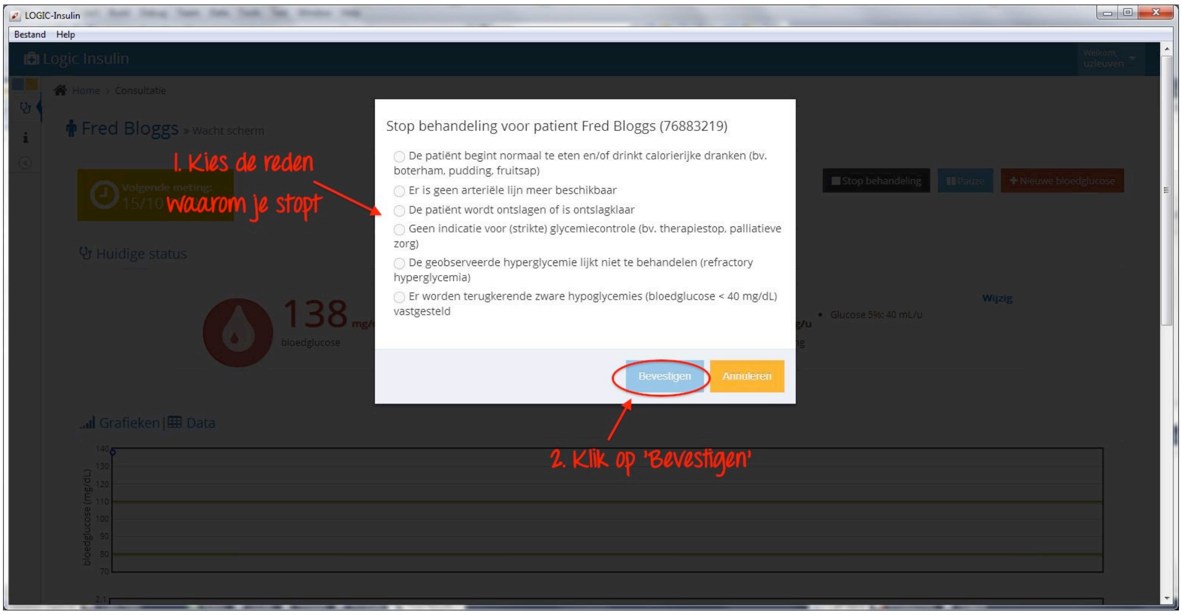

Figure 7.9. *Wacht* scherm: Bevestiging stop LOGIC-Insulin behandeling

Het programma kan nu afgesloten worden. Indien gewenst, kan later steeds een nieuwe behandeling bij deze patiënt gestart worden.

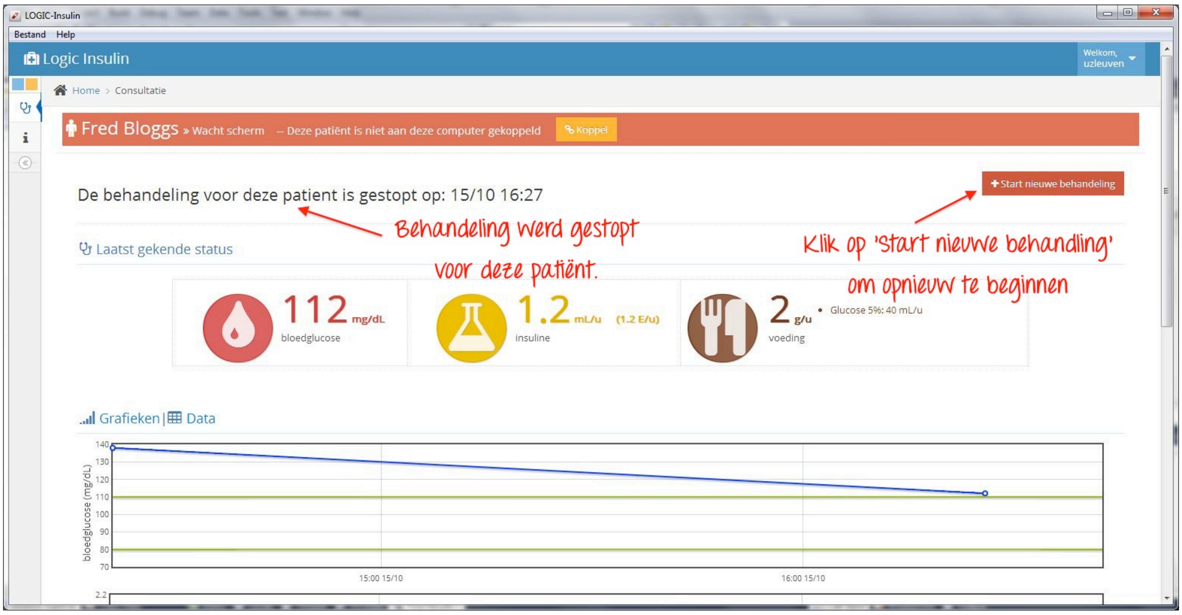

Figure 7.10. *Wacht* scherm: Behandeling met LOGIC-Insulin werd gestopt

Chapter 8. Afmelden

Klik op het pijltje in de rechterbovenhoek. Een menu verschijnt waar je *Afmelden* kan kiezen. Wanneer je hier op klikt, word je afgemeld en verschijnt het *Log in* scherm opnieuw.

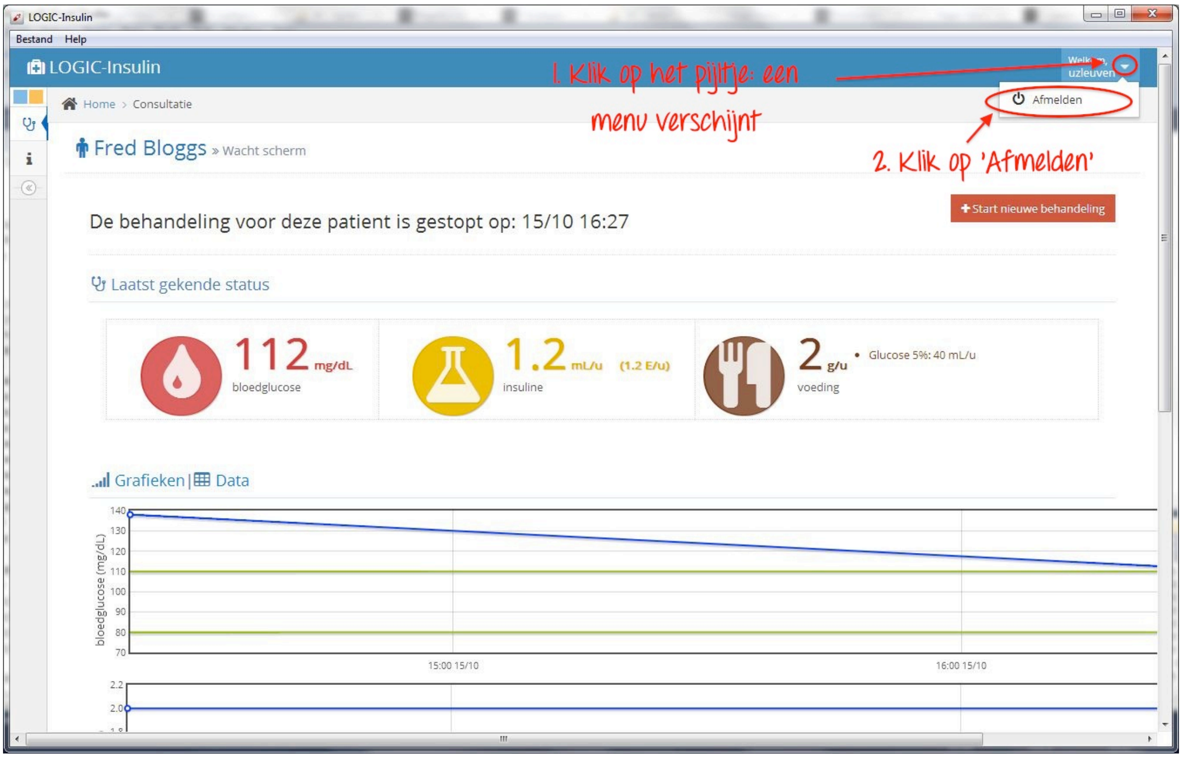

Figure 8.1. Afmelden

Chapter 9. Praktische situaties

1. **Opname nieuwe patiënt**:
   - Typisch grote drukte: Wacht met insuline (bv. opstart 1 uur na opname niet erg).
   - Vraag eerst advies aan de software, start dan pas insulinepomp op.
   - Als patiënt reeds insuline krijgt van tijdens operatie, dan mag je die "negeren" (aangezien belangrijke info kan ontbreken: bv. hoelang loopt infuus aan dat debiet?). Bij voorkeur LOGIC-Insulin starten zonder die voorkennis.
2. **Probeer zoveel mogelijk het insuline-advies op te volgen**. Graag vermijden van "onnodige" correcties (bv. correctie van 2.6 E/u naar 2.5 E/u). Corrigeer enkel als je een ernstige hypoglycemie verwacht (bv. omwille van een voorheen verkeerd aangesloten insuline-infuus).
3. **Gelieve veranderingen van de voeding in te voeren**: hetzij via de knop *Nieuwe bloedglucose*, hetzij via *Wijzig voeding* (indien voeding zou veranderen vóór het meten van een nieuwe bloedglucose) in het *Wacht* scherm
4. Medicatie opgelost in glucose 5% wordt **niet** beschouwd als "voeding". Bijgevolg moet dit **niet** worden ingevoerd in LOGIC-Insulin.
5. "Correctie" van vorige data-invoer (bv. verkeerde voeding ingevuld). Dit is mogelijk indien je deze correctie uitvoert binnen de 30 minuten (!). Klik in het *Wacht* scherm op *Nieuwe bloedglucose*. De software zal vragen of je een nieuwe waarde wil ingeven of of je een correctie wil maken. Kies voor de correctie en geef alle waarden opnieuw in via het *Consultatie* scherm Het retro-actief wijzigen/invoeren van data langer dan een half uur geleden is niet mogelijk.
6. Ernstige hypoglycemie. LOGIC-Insulin adviseert ook de glucosebolussen. Indien je de gemeten bloedglucose **niet** vertrouwt (bv. onverwachte hypo, mogelijks te wijten aan sensorfout?), **gelieve toch eerste de glucosebolus toe te dienen**. Pas nadien kan je eventueel een controlemeting uitvoeren.
7. Geef enkel **arteriële** bloedglucose-metingen in; dus geen veneuze metingen!
8. Indien een extra bloedgas beschikbaar zou zijn (omwille van een andere reden dan de bloedglucose), mag je die extra bloedglucose-waarde zeker invoeren in LOGIC-Insulin. Het tijdsinterval voor de volgende glycemie zal in de meeste gevallen worden aangepast.
9. Crash computer: Gebruik LOGIC-Insulin tijdelijk vanop een andere computer. Zoek de patiënt via zijn patient ID of naam in *Patiënt* overzicht scherm. Controleer nadien,

bv. bij het invoeren van nieuwe patiëntgegevens, of je bent aangelogd bij de juiste patiënt.

Glossarium

gekoppelde patiënt	Wanneer een patiënt gekoppeld wordt aan een computer, zal de LOGIC-Insulin software -na het inloggen- automatisch het *Wacht* scherm tonen van deze patiënt.

Appendix 6: Identification of any tissues of animal origin

We here declare that the medical device under study is not manufactured utilising tissues of animal origin.

D. Mesotten
07-01-2014

Prof. Dr. Dieter Mesotten

Signature Clinical Investigator

Appendix 7: Informed consent (UZ Leuven)

Three different informed consents are applicable:

1. Informed consent for patient
2. Informed consent for legal representative of patient
3. Informed consent for patient after consenting by legal representative

The three forms are included in the following pages.

UZ LEUVEN

INTENSIEVE GENEESKUNDE

Adressogram

Studie "Strikte bloedsuikercontrole: verpleegkundige controle versus adviserend computeralgoritme" (LOGIC-2 studie)

Nurse-directed versus algorithm-assisted Tight Blood Glucose Control in adult critically ill patients: the LOGIC-2 multi-centre randomised controlled trial

Informed consent formulier

Versienummer 2
16-07-2013

Geachte Mevrouw, Geachte Heer,

Naar aanleiding van een uitgebreide heelkundige ingreep of ernstige ziekte zal u opgenomen worden op de afdeling Intensieve Zorgen. Het medisch en verpleegkundig team van de dienst Intensieve Geneeskunde zal er alles aan doen om uw toestand zo snel mogelijk te verbeteren. Om de overlevingskansen en de kwaliteit van overleving te optimaliseren, is het uitermate belangrijk dat de bloedsuikerspiegel normaal wordt gehouden. Dit houdt in dat deze bloedsuikerspiegel frequent zal gecontroleerd worden en indien te hoog, zal gecorrigeerd worden met een insuline-infuus.

Tot op heden gebeurde deze aanpassing van het insuline-infuus door de verpleegkundige, gebaseerd op een infuusschema en op eigen ervaring. Dit gebeurt met goed resultaat. Om de controle van de bloedsuikerspiegel in de toekomst nog te verbeteren en om toe te laten u nog beter te verzorgen, werd er aan de KU Leuven een computerprogramma (LOGIC-Insulin) ontwikkeld dat advies geeft bij het aanpassen van het insuline-infuus. Het LOGIC-Insulin computerprogramma berekent een advies voor insuline-dosage bij elke bepaling van de bloedsuikerspiegel of wanneer de voedingstoediening sterk gaat veranderen. LOGIC-Insulin streeft ernaar zo snel mogelijk normale bloedsuikerspiegels te bereiken en tegelijkertijd te lage en te hoge bloedsuikerwaarden te vermijden. De voorgestelde insuline-dosage wordt door de verpleegkundige, op basis van zijn/haar expertise, geëvalueerd. De verpleegkundige behoudt te alle tijden het recht om het advies indien nodig te corrigeren.

Te lage bloedsuikerspiegels (hypoglycemie) vormen een belangrijk risico dat typisch geassocieerd is met het normaliseren van de bloedsuikerspiegels. De overbehandeling van hypoglycemie kan eveneens aanleiding geven tot sterke schommelingen van de bloedsuikerspiegels (hoge variabiliteit). Zowel te lage, te hoge (hyperglycemie) alsook sterk schommelende bloedsuikerspiegels worden geassocieerd met een minder gunstig genezingsproces van kritieke ziekte. Bij de klassieke manier van werken, tracht de verpleegkundige op basis van zijn/haar expertise hypoglycemie, hyperglycemie en hoge glucosevariabiliteit te vermijden. Dezelfde doelstelling wordt gehanteerd bij het LOGIC-Insulin computerprogramma. Een aanpassing van de hoeveelheid toe te dienen insuline of eventueel een extra toe te dienen glucosehoeveelheid wordt geadviseerd. Een alarm zal tevens waarschuwen wanneer de periode voor een nieuwe bepaling van de bloedsuikerspiegel wordt overschreden.

Mogelijk voordeel van deze studie is dat dosering van insuline efficiënter en veiliger verloopt. De behandeling met het LOGIC-Insulin computerprogramma zal ten slotte gestaakt worden in geval van herhaaldelijke hypoglycemie en oncorrigeerbare hyperglycemie.

De studie gebeurt enkel tijdens het verblijf op de intensieve zorgenafdeling en legt u geen beperking op. Bij ontslag uit de intensieve zorgenafdeling en bij het ziekenhuisontslag zullen we eveneens een vragenlijst i.v.m. de levenskwaliteit bezorgen. Het invullen hiervan vraagt slechts enkele minuten.

We vragen dan hierbij ook uw toestemming voor deelname aan deze studie. De studie zal 1550 patiënten rekruteren en heeft gelijktijdig plaats in het UZ Leuven, het Jessa Ziekenhuis in Hasselt en het AMC in Amsterdam, Nederland. Indien u akkoord bent, zullen we uw bloedsuikerspiegel normaliseren hetzij op de klassieke manier, hetzij op advies van het computerprogramma. Deze toewijzing gebeurt door middel van toevalstrekking.

Deze studie brengt geen enkele extra kost met zich mee. U kan steeds vragen om de studie stop te zetten indien u dat wenst. Gedurende het hele verblijf op de Intensieve Zorgen - afdeling krijgt u uiteraard alle nodige zorgen en behandelingen volgens de huidige stand van de geneeskunde. De medische gegevens zullen gecodeerd worden. Na de studie zullen deze worden verwerkt in een wetenschappelijke publicatie. Strikte bewaring van de anonimiteit wordt hierbij gegarandeerd.

Conform de Belgische wet inzake experimenten op de menselijke persoon van 7 mei 2004 is de opdrachtgever van het onderzoek zelfs foutloos, aansprakelijk voor alle schade die de deelnemer of, in geval van overlijden, zijn rechthebbenden oplopen en die rechtstreeks dan wel onrechtstreeks verband houdt met het experiment. De opdrachtgever heeft een verzekering afgesloten die deze aansprakelijkheid dekt. Indien u schade zou oplopen ten gevolge van uw deelname aan deze studie zal die schade bijgevolg worden vergoed conform de Belgische wet inzake experimenten op de menselijke persoon van 7 mei 2004. De gegevens voor de verzekering zijn: Vanbreda Risk & Benefits NV, Plantin en Moretuslei 297, 2140 Antwerpen (polisnummer 299.053.700)

Deze studie werd goedgekeurd door de Commissie voor Medische Ethiek van de UZ Leuven, die als centrale commissie fungeert, na raadpleging van de ethische commissies

van de andere deelnemende ziekenhuizen. Deze studie wordt uitgevoerd volgens de richtlijnen voor de goede klinische praktijk (ICH/GCP) en volgens de meest recente versie van de verklaring van Helsinki opgesteld ter bescherming van mensen deelnemend aan klinische studies. In geen geval dient u de goedkeuring door de Commissie voor Medische Ethiek te beschouwen als een aansporing tot deelname aan deze studie.

Indien u bijkomende inlichtingen wenst, kan u met uw vragen steeds terecht bij een van de onderzoekers. Bij klachten kan u zich steeds wenden tot de ombudsdienst.

Wij danken u alvast voor uw bereidwillige medewerking.

Met de meeste hoogachting,

Prof. Dr. Dieter Mesotten (coördinator)
Adjunct-kliniekhoofd

Prof. Dr. Greet Van den Berghe
Diensthoofd

Dienst intensieve geneeskunde

UZ Leuven, Herestraat 49, 3000 Leuven
Tel: 016-34 40 21, Fax: 016-34 40 15, Email: intensieve_geneeskunde@uzleuven.be

Ombudsdienst UZ Leuven

UZ Leuven, Herestraat 49, 3000 Leuven
T 016 34 48 18, Fax 016 34 46 55, Email: ombudsdienst@uzleuven.be

Toestemmingsverklaring

Ik heb de informatiebrief voor de patiënten gelezen. Ik kon aanvullende vragen stellen. Mijn vragen zijn genoeg beantwoord. Ik had genoeg tijd om te beslissen of ik meedoe.

Ik weet dat meedoen helemaal vrijwillig is. Ik weet dat ik op ieder moment kan beslissen om toch niet mee te doen. Daarvoor hoef ik geen reden te geven.

Ik geef toestemming om mijn verwijzende specialist in te lichten over deelname aan het onderzoek.

Ik geef toestemming om mijn gegevens te gebruiken, voor de doelen die in de informatiebrief staan.

Ik geef toestemming om mijn gegevens nog maximaal 20 jaar na afloop van dit onderzoek te bewaren.

Ik geef toestemming voor deelname aan het onderzoek.

Naam patiënt: ………………………………………………………………………………………

Handtekening:………………………………………………………………………………………

Datum: ………………………………………………………………………………………………….

Ik ondergetekende, bevestig dat ik mondeling de nodige informatie heb gegeven over deze klinische studie, dat ik een kopie heb gegeven van de informatie- en toestemmingsfolder die door de verschillende partijen werd getekend, dat ik bereid ben om zo nodig alle aanvullende vragen te beantwoorden en dat ik geen druk op de patiënt heb uitgeoefend om aan de studie deel te nemen. Ik verklaar dat ik werk volgens de ethische principes die worden beschreven in de Verklaring van Helsinki en de Belgische wet van 7/5/2004 over proeven op mensen.

Naam arts:…………………………………………………………………………………………….

Handtekening:……………………………………………………………………………………….

Datum:…………………………………………………………………………………………….….

INTENSIEVE GENEESKUNDE

Adressogram

Studie "Strikte bloedsuikercontrole: verpleegkundige controle versus adviserend computeralgoritme" (LOGIC-2 studie)

Nurse-directed versus algorithm-assisted Tight Blood Glucose Control in adult critically ill patients: the LOGIC-2 multi-centre randomised controlled trial

Informed consent formulier — Versienummer 2
Wettelijke vertegenwoordiger — 16-07-2013

Geachte Mevrouw, Geachte Heer,

Recent werd een familielid van u opgenomen op de afdeling Intensieve Zorgen met een ernstig medisch probleem. Uiteraard zal het medisch en verpleegkundig team van de dienst Intensieve Geneeskunde er alles aan doen om de toestand van uw familielid zo snel mogelijk te verbeteren. Om de overlevingskansen en de kwaliteit van overleving te verbeteren, is het uitermate belangrijk dat de bloedsuikerspiegel normaal wordt gehouden. Dit houdt in dat deze bloedsuikerspiegel frequent zal gecontroleerd worden en indien te hoog, zal gecorrigeerd worden met een insuline-infuus.

Tot op heden gebeurde deze aanpassing van het insuline-infuus door de verpleegkundige, gebaseerd op een infuusschema en op eigen ervaring. Dit gebeurt met goed resultaat. Om de controle van de bloedsuikerspiegel in de toekomst nog te verbeteren en om toe te laten uw familielid nog beter te verzorgen, werd er aan de KU Leuven een computerprogramma (LOGIC-Insulin) ontwikkeld dat advies geeft bij het aanpassen van het insuline-infuus. Het LOGIC-Insulin computerprogramma berekent een advies voor insuline-dosage bij elke bepaling van de bloedsuikerspiegel of wanneer de voedingstoediening sterk gaat veranderen. LOGIC-Insulin streeft ernaar zo snel mogelijk normale bloedsuikerspiegels te bereiken en tegelijkertijd te lage en te hoge bloedsuikerwaarden te vermijden. De voorgestelde insuline-dosage wordt door de verpleegkundige, op basis van zijn/haar expertise, geëvalueerd. De verpleegkundige behoudt te alle tijden het recht om het advies indien nodig te corrigeren.

Te lage bloedsuikerspiegels (hypoglycemie) vormen een belangrijk risico dat typisch geassocieerd is met het normaliseren van de bloedsuikerspiegels. De overbehandeling van hypoglycemie kan eveneens aanleiding geven tot sterke schommelingen van de bloedsuikerspiegels (hoge variabiliteit). Zowel te lage, te hoge (hyperglycemie) alsook sterk schommelende bloedsuikerspiegels worden geassocieerd met een minder gunstig genezingsproces van kritieke ziekte. Bij de klassieke manier van werken, tracht de verpleegkundige op basis van zijn/haar expertise hypoglycemie, hyperglycemie en hoge glucosevariabiliteit te vermijden. Dezelfde doelstelling wordt gehanteerd bij het LOGIC-Insulin computerprogramma. Een aanpassing van de hoeveelheid toe te dienen insuline of eventueel een extra toe te dienen glucosehoeveelheid wordt geadviseerd. Een alarm zal tevens waarschuwen wanneer de periode voor een nieuwe bepaling van de bloedsuikerspiegel wordt overschreden.

Mogelijk voordeel van deze studie is dat dosering van insuline efficiënter en veiliger verloopt. De behandeling met het LOGIC-Insulin computerprogramma zal ten slotte gestaakt worden in geval van herhaaldelijke hypoglycemie en oncorrigeerbare hyperglycemie.

De studie gebeurt enkel tijdens het verblijf op de intensieve zorgenafdeling en legt u of uw naaste geen beperking op. Bij ontslag uit de intensieve zorgenafdeling en bij het ziekenhuisontslag zullen we eveneens een vragenlijst i.v.m. de levenskwaliteit bezorgen. Het invullen hiervan vraagt slechts enkele minuten.

We vragen dan hierbij ook uw toestemming om uw familielid deel te laten nemen aan deze studie. De studie zal 1550 patiënten rekruteren en heeft gelijktijdig plaats in het UZ Leuven, het Jessa Ziekenhuis in Hasselt en het AMC in Amsterdam, Nederland. Indien u akkoord bent, zullen we de bloedsuikerspiegel bij uw familielid normaliseren hetzij op de klassieke manier, hetzij op advies van het computerprogramma. Deze toewijzing gebeurt door middel van toevalstrekking.

Deze studie brengt geen enkele extra kost met zich mee. U kan steeds vragen om de studie stop te zetten indien u dat wenst. Gedurende het hele verblijf op de Intensieve Zorgen - afdeling krijgt uw familielid uiteraard alle nodige zorgen en behandelingen volgens de huidige stand van de geneeskunde. De medische gegevens zullen gecodeerd worden. Na de studie zullen deze worden verwerkt in een wetenschappelijke publicatie. Strikte bewaring van de anonimiteit wordt hierbij gegarandeerd.

Conform de Belgische wet inzake experimenten op de menselijke persoon van 7 mei 2004 is de opdrachtgever van het onderzoek zelfs foutloos, aansprakelijk voor alle schade die de deelnemer of, in geval van overlijden, zijn rechthebbenden oplopen en die rechtstreeks dan wel onrechtstreeks verband houdt met het experiment. De opdrachtgever heeft een verzekering afgesloten die deze aansprakelijkheid dekt. Indien u schade zou oplopen ten gevolge van uw deelname aan deze studie zal die schade bijgevolg worden vergoed conform de Belgische wet inzake experimenten op de menselijke persoon van 7 mei 2004. De gegevens voor de verzekering zijn: Vanbreda Risk & Benefits NV, Plantin en Moretuslei 297, 2140 Antwerpen (polisnummer 299.053.700)

Deze studie werd goedgekeurd door de Commissie voor Medische Ethiek van de UZ Leuven, die als centrale commissie fungeert, na raadpleging van de ethische commissies van de andere deelnemende ziekenhuizen.

Bijzondere bepalingen voor de bescherming van personen van wie de toestemming niet kan worden verkregen wegens hoogdringendheid.
De wet van 7 mei 2004 inzake experimenten op de menselijke persoon legt vast aan welke voorwaarden moeten voldaan zijn indien de toestemming van de patiënt niet kon verkregen worden wegens hoogdringendheid. Het gaat om een levensbedreigende toestand die tot ernstige en blijvende letsels kan leiden, of de dood. Een gunstig advies werd gegeven door de Commissie Medische Ethiek van UZ/KU Leuven. Deze Commissie sprak zich uitdrukkelijk uit over de uitzondering op de regel van de geïnformeerde toestemming voorafgaand aan het experiment. De wet bepaalt ook dat u/de patiënt toestemming dient te geven van zodra u/de patiënt daartoe in staat bent/is, en dat u/de patiënt toestemming geeft voor het verder verzamelen van studiegegevens. Een vertegenwoordiger dient toestemming te geven van zodra het mogelijk is contact met deze persoon op te nemen.

Deze studie wordt uitgevoerd volgens de richtlijnen voor de goede klinische praktijk (ICH/GCP) en volgens de meest recente versie van de verklaring van Helsinki opgesteld ter bescherming van mensen deelnemend aan klinische studies. In geen geval dient u de goedkeuring door de Commissie voor Medische Ethiek te beschouwen als een aansporing tot deelname aan deze studie.

Indien u bijkomende inlichtingen wenst, kan u met uw vragen steeds terecht bij een van de onderzoekers. Bij klachten kan u zich steeds wenden tot de ombudsdienst.

Wij danken u alvast voor uw bereidwillige medewerking.

Met de meeste hoogachting,

Prof. Dr. Dieter Mesotten (coördinator)
Adjunct-kliniekhoofd

Prof. Dr. Greet Van den Berghe
Diensthoofd

Dienst intensieve geneeskunde

UZ Leuven, Herestraat 49, 3000 Leuven
Tel: 016-34 40 21, Fax: 016-34 40 15, Email: intensieve_geneeskunde@uzleuven.be

Ombudsdienst UZ Leuven

UZ Leuven, Herestraat 49, 3000 Leuven
T 016 34 48 18, Fax 016 34 46 55, Email: ombudsdienst@uzleuven.be

Toestemmingsverklaring - wettelijk vertegenwoordigers

Ik heb de informatiebrief voor de wettelijke vertegenwoordiger gelezen. Ik kon aanvullende vragen stellen. Mijn vragen zijn genoeg beantwoord. Ik heb voldoende tijd gehad om te beslissen of de betreffende patiënt meedoet.

Ik weet dat meedoen helemaal vrijwillig is. Ik weet dat ik op ieder moment kan beslissen dat deze persoon toch niet meedoet. Daarvoor hoef ik geen reden te geven.

Ik geef toestemming om de verwijzende specialist van betreffende patiënt in te lichten over deelname aan het onderzoek.

Ik geef toestemming om de gegevens te gebruiken, voor de doelen die in de informatiebrief staan.

Ik geef toestemming om de gegevens van deze persoon nog maximaal 20 jaar na afloop van dit onderzoek te bewaren.

Ik geef toestemming voor deelname van betreffende patiënt aan dit onderzoek. De toestemming van uw familielid zelf zal gevraagd worden van zodra zijn/haar toestand dit toelaat.

Naam patiënt: ……………………………………………………………………………………

Naam en relatie tot patiënt van ondergetekende:…………………………………………….....
....

Datum: ……………………………………………………………………………………………

Handtekening:…………………………………………………………………………………

Ik ondergetekende, bevestig dat ik mondeling de nodige informatie heb gegeven over deze klinische studie, dat ik een kopie heb gegeven van de informatie- en toestemmingsfolder die door de verschillende partijen werd getekend, dat ik bereid ben om zo nodig alle aanvullende vragen te beantwoorden en dat ik geen druk op de patiënt heb uitgeoefend om aan de studie deel te nemen. Ik verklaar dat ik werk volgens de ethische principes die worden beschreven in de Verklaring van Helsinki en de Belgische wet van 7/5/2004 over proeven op mensen.

Naam Arts:……………………………………………………………………………..………

Handtekening:……………………………………………………………………………..…..

Datum:…………………………………………………………………………………..…....

UZ LEUVEN

Adressogram

INTENSIEVE GENEESKUNDE

Studie "Strikte bloedsuikercontrole: verpleegkundige controle versus adviserend computeralgoritme" (LOGIC-2 studie)

Nurse-directed versus algorithm-assisted Tight Blood Glucose Control in adult critically ill patients: the LOGIC-2 multi-centre randomised controlled trial

Informed consent formulier
Patiënt achteraf

Versienummer 1
16-07-2013

Geachte Mevrouw, Geachte Heer,

Kort na uw opname op de intensieve zorgenafdeling heeft uw wettelijke vertegenwoordiger (bijvoorbeeld een familielid) toestemming gegeven voor deelname aan deze studie. Via deze brief willen we uw toestemming vragen voor het eerder doen van dat onderzoek.

Het medisch en verpleegkundig team van de dienst Intensieve Geneeskunde heeft er alles aan gedaan om uw toestand zo snel mogelijk te verbeteren. Om de overlevingskansen en de kwaliteit van overleving te optimaliseren, is het uitermate belangrijk dat de bloedsuikerspiegel normaal wordt gehouden. Dit houdt in dat deze bloedsuikerspiegel frequent gecontroleerd wordt en indien te hoog, gecorrigeerd met een insuline-infuus. Tot op heden gebeurde deze aanpassing van het insuline-infuus door de verpleegkundige, gebaseerd op een infuusschema en op eigen ervaring. Dit gebeurt met goed resultaat. Om de controle van de bloedsuikerspiegel in de toekomst nog te verbeteren en om toe te laten u nog beter te verzorgen, werd er aan de KU Leuven een computerprogramma (LOGIC-Insulin) ontwikkeld dat advies geeft bij het aanpassen van het insuline-infuus. Het LOGIC-Insulin computerprogramma berekent een advies voor insuline-dosage bij elke bepaling van de bloedsuikerspiegel of wanneer de voedingstoediening sterk gaat veranderen. LOGIC-Insulin streeft ernaar zo snel mogelijk normale bloedsuikerspiegels te bereiken en tegelijkertijd te lage en te hoge bloedsuikerwaarden te vermijden. De voorgestelde insuline-dosage wordt door de verpleegkundige, op basis van zijn/haar expertise, geëvalueerd. De verpleegkundige behoudt te alle tijden het recht om het advies indien nodig te corrigeren.

Te lage bloedsuikerspiegels (hypoglycemie) vormen een belangrijk risico dat typisch geassocieerd is met het normaliseren van de bloedsuikerspiegels. De overbehandeling van hypoglycemie kan eveneens aanleiding geven tot sterke schommelingen van de bloedsuikerspiegels (hoge variabiliteit). Zowel te lage, te hoge (hyperglycemie) alsook sterk schommelende bloedsuikerspiegels worden geassocieerd met een minder gunstig genezingsproces van kritieke ziekte. Bij de klassieke manier van werken, tracht de verpleegkundige op basis van zijn/haar expertise hypoglycemie, hyperglycemie en hoge glucosevariabiliteit te vermijden. Dezelfde doelstelling wordt gehanteerd bij het LOGIC-Insulin computerprogramma. Een aanpassing van de hoeveelheid toe te dienen insuline of eventueel een extra toe te dienen glucosehoeveelheid wordt geadviseerd. Een alarm zal tevens waarschuwen wanneer de periode voor een nieuwe bepaling van de bloedsuikerspiegel wordt overschreden.

Mogelijk voordeel van deze studie is dat dosering van insuline efficiënter en veiliger verloopt. De behandeling met het LOGIC-Insulin computerprogramma zal ten slotte gestaakt worden in geval van herhaaldelijke hypoglycemie en oncorrigeerbare hyperglycemie.

De studie gebeurt enkel tijdens het verblijf op de intensieve zorgenafdeling en legt u geen beperking op. Bij ontslag uit de intensieve zorgenafdeling en bij het ziekenhuisontslag zullen we eveneens een vragenlijst i.v.m. de levenskwaliteit bezorgen. Het invullen hiervan vraagt slechts enkele minuten.

De studie zal 1550 patiënten rekruteren en heeft gelijktijdig plaats in het UZ Leuven, het Jessa Ziekenhuis in Hasselt en het AMC in Amsterdam, Nederland. Uw bloedsuikerspiegel werd genormaliseerd hetzij op de klassieke manier, hetzij op advies van het computerprogramma. Deze toewijzing gebeurde door middel van toevalstrekking.

Deze studie brengt geen enkele extra kost met zich mee. U kan steeds vragen om de studie stop te zetten indien u dat wenst. Gedurende het hele verblijf op de Intensieve Zorgen - afdeling krijgt u uiteraard alle nodige zorgen en behandelingen volgens de huidige stand van de geneeskunde. De medische gegevens zullen gecodeerd worden. Na de studie zullen deze worden verwerkt in een wetenschappelijke publicatie. Strikte bewaring van de anonimiteit wordt hierbij gegarandeerd.

Conform de Belgische wet inzake experimenten op de menselijke persoon van 7 mei 2004 is de opdrachtgever van het onderzoek zelfs foutloos, aansprakelijk voor alle schade die de deelnemer of, in geval van overlijden, zijn rechthebbenden oplopen en die rechtstreeks dan wel onrechtstreeks verband houdt met het experiment. De opdrachtgever heeft een verzekering afgesloten die deze aansprakelijkheid dekt. Indien u schade zou oplopen ten gevolge van uw deelname aan deze studie zal die schade bijgevolg worden vergoed conform de Belgische wet inzake experimenten op de menselijke persoon van 7 mei 2004. De gegevens voor de verzekering zijn: Vanbreda Risk & Benefits NV, Plantin en Moretuslei 297, 2140 Antwerpen (polisnummer 299.053.700)

Deze studie werd goedgekeurd door de Commissie voor Medische Ethiek van de UZ Leuven, die als centrale commissie fungeert, na raadpleging van de ethische commissies van de andere deelnemende ziekenhuizen.

Bijzondere bepalingen voor de bescherming van personen van wie de toestemming niet kan worden verkregen wegens hoogdringendheid.
De wet van 7 mei 2004 inzake experimenten op de menselijke persoon legt vast aan welke voorwaarden moeten voldaan zijn indien de toestemming van de patiënt niet kon verkregen worden wegens hoogdringendheid. Het gaat om een levensbedreigende toestand die tot ernstige en blijvende letsels kan leiden, of de dood. Een gunstig advies werd gegeven door de Commissie Medische Ethiek van UZ/KU Leuven. Deze Commissie sprak zich uitdrukkelijk uit over de uitzondering op de regel van de geïnformeerde toestemming voorafgaand aan het experiment. De wet bepaalt ook dat u/de patiënt toestemming dient te geven van zodra u/de patiënt daartoe in staat bent/is, en dat u/de patiënt toestemming geeft voor het verder verzamelen van studiegegevens. Een vertegenwoordiger dient toestemming te geven van zodra het mogelijk is contact met deze persoon op te nemen.

Deze studie wordt uitgevoerd volgens de richtlijnen voor de goede klinische praktijk (ICH/GCP) en volgens de meest recente versie van de verklaring van Helsinki opgesteld ter bescherming van mensen deelnemend aan klinische studies. In geen geval dient u de goedkeuring door de Commissie voor Medische Ethiek te beschouwen als een aansporing tot deelname aan deze studie.

Indien u bijkomende inlichtingen wenst, kan u met uw vragen steeds terecht bij een van de onderzoekers. Bij klachten kan u zich steeds wenden tot de ombudsdienst.

Wij danken u alvast voor uw bereidwillige medewerking.

Met de meeste hoogachting,

Prof. Dr. Dieter Mesotten (coördinator)
Adjunct-kliniekhoofd

Prof. Dr. Greet Van den Berghe
Diensthoofd

Dienst intensieve geneeskunde

UZ Leuven, Herestraat 49, 3000 Leuven
Tel: 016-34 40 21, Fax: 016-34 40 15, Email: intensieve_geneeskunde@uzleuven.be

Ombudsdienst UZ Leuven

UZ Leuven, Herestraat 49, 3000 Leuven
T 016 34 48 18, Fax 016 34 46 55, Email: ombudsdienst@uzleuven.be

---

Toestemmingsverklaring

Ik heb de informatiebrief voor de patiënten gelezen. Ik kon aanvullende vragen stellen. Mijn vragen zijn genoeg beantwoord. Ik had genoeg tijd om te beslissen of ik meedoe.

Ik weet dat meedoen helemaal vrijwillig is. Ik weet dat ik op ieder moment kan beslissen om toch niet mee te doen. Daarvoor hoef ik geen reden te geven.

Ik geef toestemming om mijn verwijzende specialist in te lichten over deelname aan het onderzoek.

Ik geef toestemming om mijn gegevens te gebruiken, voor de doelen die in de informatiebrief staan.

Ik geef toestemming om mijn gegevens nog maximaal 20 jaar na afloop van dit onderzoek te bewaren.

Ik geef toestemming voor deelname aan het onderzoek.

Naam patiënt: ……………………………………………………………………………………

Handtekening:……………………………………………………………………………………

Datum: ……………………………………………………………………………………………

Ik ondergetekende, bevestig dat ik mondeling de nodige informatie heb gegeven over deze klinische studie, dat ik een kopie heb gegeven van de informatie- en toestemmingsfolder die door de verschillende partijen werd getekend, dat ik bereid ben om zo nodig alle aanvullende vragen te beantwoorden en dat ik geen druk op de patiënt heb uitgeoefend om aan de studie deel te nemen. Ik verklaar dat ik werk volgens de ethische principes die worden beschreven in de Verklaring van Helsinki en de Belgische wet van 7/5/2004 over proeven op mensen.

Naam
Arts:…………………………………………………………………………………………

Handtekening:……………………………………………………………………………………

Datum:……………………………………………………………………………………………

Appendix 8: Informed consent (Jessa Hospital Hasselt)

Three different informed consents are applicable:

1. Informed consent for patient
2. Informed consent for legal representative of patient
3. Informed consent for patient after consenting by legal representative

The three forms are included in the following pages.

Adressogram

Dienst Anesthesie en Intensieve Geneeskunde

Studie "Strikte bloedsuikercontrole: verpleegkundige controle versus adviserend computeralgoritme" (LOGIC-2 studie)

Nurse-directed versus algorithm-assisted Tight Blood Glucose Control in adult critically ill patients: the LOGIC-2 multi-centre randomised controlled trial

Informed consent formulier

Versienummer 2
16-07-2013

Geachte Mevrouw, Geachte Heer,

Naar aanleiding van een uitgebreide heelkundige ingreep of ernstige ziekte zal u opgenomen worden op de afdeling Intensieve Zorgen. Het medisch en verpleegkundig team van de dienst Intensieve Geneeskunde zal er alles aan doen om uw toestand zo snel mogelijk te verbeteren. Om de overlevingskansen en de kwaliteit van overleving te optimaliseren, is het uitermate belangrijk dat de bloedsuikerspiegel normaal wordt gehouden. Dit houdt in dat deze bloedsuikerspiegel frequent zal gecontroleerd worden en indien te hoog, zal gecorrigeerd worden met een insuline-infuus.

Tot op heden gebeurde deze aanpassing van het insuline-infuus door de verpleegkundige, gebaseerd op een infuusschema en op eigen ervaring. Dit gebeurt met goed resultaat. Om de controle van de bloedsuikerspiegel in de toekomst nog te verbeteren en om toe te laten u nog beter te verzorgen, werd er aan de KU Leuven een computerprogramma (LOGIC-Insulin) ontwikkeld dat advies geeft bij het aanpassen van het insuline-infuus. Het LOGIC-Insulin computerprogramma berekent een advies voor insuline-dosage bij elke bepaling van de bloedsuikerspiegel of wanneer de voedingstoediening sterk gaat veranderen. LOGIC-Insulin streeft ernaar zo snel mogelijk normale bloedsuikerspiegels te bereiken en tegelijkertijd te lage en te hoge bloedsuikerwaarden te vermijden. De voorgestelde insuline-dosage wordt door de verpleegkundige, op basis van zijn/haar expertise, geëvalueerd. De verpleegkundige behoudt te alle tijden het recht om het advies indien nodig te corrigeren.

Te lage bloedsuikerspiegels (hypoglycemie) vormen een belangrijk risico dat typisch geassocieerd is met het normaliseren van de bloedsuikerspiegels. De overbehandeling van hypoglycemie kan eveneens aanleiding geven tot sterke schommelingen van de bloedsuikerspiegels (hoge variabiliteit). Zowel te lage, te hoge (hyperglycemie) alsook sterk schommelende bloedsuikerspiegels worden geassocieerd met een minder gunstig genezingsproces van kritieke ziekte. Bij de klassieke manier van werken, tracht de verpleegkundige op basis van zijn/haar expertise hypoglycemie, hyperglycemie en hoge glucosevariabiliteit te vermijden. Dezelfde doelstelling wordt gehanteerd bij het LOGIC-Insulin computerprogramma. Een aanpassing van de hoeveelheid toe te dienen insuline of eventueel een extra toe te dienen glucosehoeveelheid wordt geadviseerd. Een alarm zal tevens waarschuwen wanneer de periode voor een nieuwe bepaling van de bloedsuikerspiegel wordt overschreden.

Mogelijk voordeel van deze studie is dat dosering van insuline efficiënter en veiliger verloopt. De behandeling met het LOGIC-Insulin computerprogramma zal ten slotte gestaakt worden in geval van herhaaldelijke hypoglycemie en oncorrigeerbare hyperglycemie.

De studie gebeurt enkel tijdens het verblijf op de intensieve zorgenafdeling en legt u geen beperking op. Bij ontslag uit de intensieve zorgenafdeling en bij het ziekenhuisontslag zullen we eveneens een vragenlijst i.v.m. de levenskwaliteit bezorgen. Het invullen hiervan vraagt slechts enkele minuten.

We vragen dan hierbij ook uw toestemming voor deelname aan deze studie. De studie zal 1550 patiënten rekruteren en heeft gelijktijdig plaats in het UZ Leuven, het Jessa Ziekenhuis in Hasselt en het AMC in Amsterdam, Nederland. Indien u akkoord bent, zullen we uw bloedsuikerspiegel normaliseren hetzij op de klassieke manier, hetzij op advies van het computerprogramma. Deze toewijzing gebeurt door middel van toevalstrekking.

Deze studie brengt geen enkele extra kost met zich mee. U kan steeds vragen om de studie stop te zetten indien u dat wenst. Gedurende het hele verblijf op de Intensieve Zorgen -afdeling krijgt u uiteraard alle nodige zorgen en behandelingen volgens de huidige stand van de geneeskunde. De medische gegevens zullen gecodeerd worden. Na de studie zullen deze worden verwerkt in een wetenschappelijke publicatie. Strikte bewaring van de anonimiteit wordt hierbij gegarandeerd.

Conform de Belgische wet inzake experimenten op de menselijke persoon van 7 mei 2004 is de opdrachtgever van het onderzoek zelfs foutloos, aansprakelijk voor alle schade die de deelnemer of, in geval van overlijden, zijn rechthebbenden oplopen en die rechtstreeks dan wel onrechtstreeks verband houdt met het experiment. De opdrachtgever heeft een verzekering afgesloten die deze aansprakelijkheid dekt. Indien u schade zou oplopen ten gevolge van uw deelname aan deze studie zal die schade bijgevolg worden vergoed conform de Belgische wet inzake experimenten op de menselijke persoon van 7 mei 2004. De gegevens voor de verzekering zijn: Vanbreda Risk & Benefits NV, Plantin en Moretuslei 297, 2140 Antwerpen (polisnummer 299.053.700)

Deze studie werd goedgekeurd door de Commissie voor Medische Ethiek van de UZ Leuven, die als centrale commissie fungeert, na raadpleging van de ethische commissies van de andere deelnemende ziekenhuizen. Deze studie wordt uitgevoerd volgens de richtlijnen voor de goede klinische praktijk (ICH/GCP) en volgens de meest recente versie van de verklaring van Helsinki opgesteld ter bescherming van mensen deelnemend aan klinische studies. In geen geval dient u de goedkeuring door de Commissie voor Medische Ethiek te beschouwen als een aansporing tot deelname aan deze studie.

Indien u bijkomende inlichtingen wenst, kan u met uw vragen steeds terecht bij een van de onderzoekers.

Wij danken u alvast voor uw bereidwillige medewerking.

Met de meeste hoogachting,

Prof. Dr. Dieter Mesotten (coördinator)
Adjunct-kliniekhoofd

Prof. Dr. Greet Van den Berghe
Diensthoofd

Dienst intensieve geneeskunde
UZ Leuven
Herestraat 49
3000 Leuven
Tel: 016-344021

Dr. Jasperina Dubois
Lokale coördinator LOGIC-2

Dr. Aimé Van Assche
Diensthoofd

Dienst intensieve geneeskunde
Jessa Ziekenhuis (Campus Virga Jessa)
Stadsomvaart 11
3500 Hasselt
Tel: 011-308971

Toestemmingsverklaring

Ik heb de informatiebrief voor de patiënten gelezen. Ik kon aanvullende vragen stellen. Mijn vragen zijn genoeg beantwoord. Ik had genoeg tijd om te beslissen of ik meedoe.

Ik weet dat meedoen helemaal vrijwillig is. Ik weet dat ik op ieder moment kan beslissen om toch niet mee te doen. Daarvoor hoef ik geen reden te geven.

Ik geef toestemming om mijn verwijzende specialist in te lichten over deelname aan het onderzoek.

Ik geef toestemming om mijn gegevens te gebruiken, voor de doelen die in de informatiebrief staan.

Ik geef toestemming om mijn gegevens nog maximaal 20 jaar na afloop van dit onderzoek te bewaren.

Ik geef toestemming voor deelname aan het onderzoek.

Naam patiënt: …………………………………………………………………………………………

Handtekening:…………………………………………………………………………………………

Datum: …………………………………………………………………………………………………..

Ik ondergetekende, bevestig dat ik mondeling de nodige informatie heb gegeven over deze klinische studie, dat ik een kopie heb gegeven van de informatie- en toestemmingsfolder die door de verschillende partijen werd getekend, dat ik bereid ben om zo nodig alle aanvullende vragen te beantwoorden en dat ik geen druk op de patiënt heb uitgeoefend om aan de studie deel te nemen. Ik verklaar dat ik werk volgens de ethische principes die worden beschreven in de Verklaring van Helsinki en de Belgische wet van 7/5/2004 over proeven op mensen.

Naam Arts:……………………………………………………………………………………..……

Handtekening:…………………………………………………………………………………….…..

Datum:……………………………………………………………………………………………..…

Adressogram

Dienst Anesthesie en Intensieve Geneeskunde

Studie "Strikte bloedsuikercontrole: verpleegkundige controle versus adviserend computeralgoritme" (LOGIC-2 studie)

Nurse-directed versus algorithm-assisted Tight Blood Glucose Control in adult critically ill patients: the LOGIC-2 multi-centre randomised controlled trial

Informed consent formulier Versienummer 2
Wettelijke vertegenwoordiger 16-07-2013

Geachte Mevrouw, Geachte Heer,

Recent werd een familielid van u opgenomen op de afdeling Intensieve Zorgen met een ernstig medisch probleem. Uiteraard zal het medisch en verpleegkundig team van de dienst Intensieve Geneeskunde er alles aan doen om de toestand van uw familielid zo snel mogelijk te verbeteren. Om de overlevingskansen en de kwaliteit van overleving te verbeteren, is het uitermate belangrijk dat de bloedsuikerspiegel normaal wordt gehouden. Dit houdt in dat deze bloedsuikerspiegel frequent zal gecontroleerd worden en indien te hoog, zal gecorrigeerd worden met een insuline-infuus.

Tot op heden gebeurde deze aanpassing van het insuline-infuus door de verpleegkundige, gebaseerd op een infuusschema en op eigen ervaring. Dit gebeurt met goed resultaat. Om de controle van de bloedsuikerspiegel in de toekomst nog te verbeteren en om toe te laten uw familielid nog beter te verzorgen, werd er aan de KU Leuven een computerprogramma (LOGIC-Insulin) ontwikkeld dat advies geeft bij het aanpassen van het insuline-infuus. Het LOGIC-Insulin computerprogramma berekent een advies voor insuline-dosage bij elke bepaling van de bloedsuikerspiegel of wanneer de voedingstoediening sterk gaat veranderen. LOGIC-Insulin streeft ernaar zo snel mogelijk normale bloedsuikerspiegels te bereiken en tegelijkertijd te lage en te hoge bloedsuikerwaarden te vermijden. De voorgestelde insuline-dosage wordt door de verpleegkundige, op basis van zijn/haar expertise, geëvalueerd. De verpleegkundige behoudt te alle tijden het recht om het advies indien nodig te corrigeren.

Te lage bloedsuikerspiegels (hypoglycemie) vormen een belangrijk risico dat typisch geassocieerd is met het normaliseren van de bloedsuikerspiegels. De overbehandeling van hypoglycemie kan eveneens aanleiding geven tot sterke schommelingen van de bloedsuikerspiegels (hoge variabiliteit). Zowel te lage, te hoge (hyperglycemie) alsook sterk schommelende bloedsuikerspiegels worden geassocieerd met een minder gunstig genezingsproces van kritieke ziekte. Bij de klassieke manier van werken, tracht de verpleegkundige op basis van zijn/haar expertise hypoglycemie, hyperglycemie en hoge glucosevariabiliteit te vermijden. Dezelfde doelstelling wordt gehanteerd bij het LOGIC-Insulin computerprogramma. Een aanpassing van de hoeveelheid toe te dienen insuline of eventueel een extra toe te dienen glucosehoeveelheid wordt geadviseerd. Een alarm zal tevens waarschuwen wanneer de periode voor een nieuwe bepaling van de bloedsuikerspiegel wordt overschreden.

Mogelijk voordeel van deze studie is dat dosering van insuline efficiënter en veiliger verloopt. De behandeling met het LOGIC-Insulin computerprogramma zal ten slotte gestaakt worden in geval van herhaaldelijke hypoglycemie en oncorrigeerbare hyperglycemie.

De studie gebeurt enkel tijdens het verblijf op de intensieve zorgenafdeling en legt u of uw naaste geen beperking op. Bij ontslag uit de intensieve zorgenafdeling en bij het ziekenhuisontslag zullen we eveneens een vragenlijst i.v.m. de levenskwaliteit bezorgen. Het invullen hiervan vraagt slechts enkele minuten.

We vragen dan hierbij ook uw toestemming om uw familielid deel te laten nemen aan deze studie. De studie zal 1550 patiënten rekruteren en heeft gelijktijdig plaats in het UZ Leuven, het Jessa Ziekenhuis in Hasselt en het AMC in Amsterdam, Nederland. Indien u akkoord bent, zullen we de bloedsuikerspiegel bij uw familielid normaliseren hetzij op de klassieke manier, hetzij op advies van het computerprogramma. Deze toewijzing gebeurt door middel van toevalstrekking.

Deze studie brengt geen enkele extra kost met zich mee. U kunt steeds vragen om de studie stop te zetten indien u dat wenst. Gedurende het hele verblijf op de Intensieve Zorgen - afdeling krijgt uw familielid uiteraard alle nodige zorgen en behandelingen volgens de huidige stand van de geneeskunde. De medische gegevens zullen gecodeerd worden. Na de studie zullen deze worden verwerkt in een wetenschappelijke publicatie. Strikte bewaring van de anonimiteit wordt hierbij gegarandeerd.

Conform de Belgische wet inzake experimenten op de menselijke persoon van 7 mei 2004 is de opdrachtgever van het onderzoek zelfs foutloos, aansprakelijk voor alle schade die de deelnemer of, in geval van overlijden, zijn rechthebbenden oplopen en die rechtstreeks dan wel onrechtstreeks verband houdt met het experiment. De opdrachtgever heeft een verzekering afgesloten die deze aansprakelijkheid dekt. Indien U schade zou oplopen ten gevolge van uw deelname aan deze studie zal die schade bijgevolg worden vergoed conform de Belgische wet inzake experimenten op de menselijke persoon van 7 mei 2004. De gegevens voor de verzekering zijn: Vanbreda Risk & Benefits NV, Plantin en Moretuslei 297, 2140 Antwerpen (polisnummer 299.053.700)

Deze studie werd goedgekeurd door de Commissie voor Medische Ethiek van de UZ Leuven, die als centrale commissie fungeert, na raadpleging van de ethische commissies van de andere deelnemende ziekenhuizen.

Bijzondere bepalingen voor de bescherming van personen van wie de toestemming niet kan worden verkregen wegens hoogdringendheid.
De wet van 7 mei 2004 inzake experimenten op de menselijke persoon legt vast aan welke voorwaarden moeten voldaan zijn indien de toestemming van de patiënt niet kon verkregen worden wegens hoogdringendheid. Het gaat om een levensbedreigende toestand die tot ernstige en blijvende letsels kan leiden, of de dood. Een gunstig advies werd gegeven door de Commissie Medische Ethiek van UZ/KU Leuven. Deze Commissie sprak zich uitdrukkelijk uit over de uitzondering op de regel van de geïnformeerde toestemming voorafgaand aan het experiment. De wet bepaalt ook dat u/de patiënt toestemming dient te geven van zodra u/de patiënt daartoe in staat bent/is, en dat u/de patiënt toestemming geeft voor het verder verzamelen van studiegegevens. Een vertegenwoordiger dient toestemming te geven van zodra het mogelijk is contact met deze persoon op te nemen.

Deze studie wordt uitgevoerd volgens de richtlijnen voor de goede klinische praktijk (ICH/GCP) en volgens de meest recente versie van de verklaring van Helsinki opgesteld ter bescherming van mensen deelnemend aan klinische studies. In geen geval dient u de goedkeuring door de Commissie voor Medische Ethiek te beschouwen als een aansporing tot deelname aan deze studie.

Indien u bijkomende inlichtingen wenst, kan u met uw vragen steeds terecht bij een van de onderzoekers.

Wij danken u alvast voor uw bereidwillige medewerking.

Met de meeste hoogachting,

Prof. Dr. Dieter Mesotten (coördinator)
Adjunct-kliniekhoofd

Prof. Dr. Greet Van den Berghe
Diensthoofd

Dienst intensieve geneeskunde
UZ Leuven
Herestraat 49
3000 Leuven
Tel: 016-344021

Dr. Jasperina Dubois
Lokale coördinator LOGIC-2

Dr. Aimé Van Assche
Diensthoofd

Dienst intensieve geneeskunde
Jessa Ziekenhuis (Campus Virga Jessa)
Stadsomvaart 11
3500 Hasselt
Tel: 011-308971

Toestemmingsverklaring - wettelijk vertegenwoordigers

Ik heb de informatiebrief voor de wettelijke vertegenwoordiger gelezen. Ik kon aanvullende vragen stellen. Mijn vragen zijn genoeg beantwoord. Ik heb voldoende tijd gehad om te beslissen of de betreffende patiënt meedoet.

Ik weet dat meedoen helemaal vrijwillig is. Ik weet dat ik op ieder moment kan beslissen dat deze persoon toch niet meedoet. Daarvoor hoef ik geen reden te geven.

Ik geef toestemming om de verwijzende specialist van betreffende patiënt in te lichten over deelname aan het onderzoek.

Ik geef toestemming om de gegevens te gebruiken, voor de doelen die in de informatiebrief staan.

Ik geef toestemming om de gegevens van deze persoon nog maximaal 20 jaar na afloop van dit onderzoek te bewaren.

Ik geef toestemming voor deelname van betreffende patiënt aan dit onderzoek. De toestemming van uw familielid zelf zal gevraagd worden van zodra zijn/haar toestand dit toelaat.

Naam patiënt: ……………………………………………………………………………………

Naam en relatie tot patiënt van ondergetekende:……………………………………………....
....

Datum: ………………………………………………………………………………………….

Handtekening:……………………………………………………………………………………

Ik ondergetekende, bevestig dat ik mondeling de nodige informatie heb gegeven over deze klinische studie, dat ik een kopie heb gegeven van de informatie- en toestemmingsfolder die door de verschillende partijen werd getekend, dat ik bereid ben om zo nodig alle aanvullende vragen te beantwoorden en dat ik geen druk op de patiënt heb uitgeoefend om aan de studie deel te nemen. Ik verklaar dat ik werk volgens de ethische principes die worden beschreven in de Verklaring van Helsinki en de Belgische wet van 7/5/2004 over proeven op mensen.

Naam Arts:……………………………………………………………………………..………

Handtekening:………………………………………………………………………….……..

Datum:…………………………………………………………………………………..…...

Adressogram

Dienst Anesthesie en Intensieve Geneeskunde

Studie “Strikte bloedsuikercontrole: verpleegkundige controle versus adviserend computeralgoritme” (LOGIC-2 studie)

Nurse-directed versus algorithm-assisted Tight Blood Glucose Control in adult critically ill patients: the LOGIC-2 multi-centre randomised controlled trial

Informed consent formulier
Patiënt achteraf

Versienummer 1
16-07-2013

Geachte Mevrouw, Geachte Heer,

Kort na uw opname op de intensieve zorgenafdeling heeft uw wettelijke vertegenwoordiger (bijvoorbeeld een familielid) toestemming gegeven voor deelname aan deze studie. Via deze brief willen we uw toestemming vragen voor het eerder doen van dat onderzoek.

Het medisch en verpleegkundig team van de dienst Intensieve Geneeskunde heeft er alles aan gedaan om uw toestand zo snel mogelijk te verbeteren. Om de overlevingskansen en de kwaliteit van overleving te optimaliseren, is het uitermate belangrijk dat de bloedsuikerspiegel normaal wordt gehouden. Dit houdt in dat deze bloedsuikerspiegel frequent gecontroleerd wordt en indien te hoog, gecorrigeerd met een insuline-infuus. Tot op heden gebeurde deze aanpassing van het insuline-infuus door de verpleegkundige, gebaseerd op een infuusschema en op eigen ervaring. Dit gebeurt met goed resultaat. Om de controle van de bloedsuikerspiegel in de toekomst nog te verbeteren en om toe te laten u nog beter te verzorgen, werd er aan de KU Leuven een computerprogramma (LOGIC-Insulin) ontwikkeld dat advies geeft bij het aanpassen van het insuline-infuus. Het LOGIC-Insulin computerprogramma berekent een advies voor insuline-dosage bij elke bepaling van de bloedsuikerspiegel of wanneer de voedingstoediening sterk gaat veranderen. LOGIC-Insulin streeft ernaar zo snel mogelijk normale bloedsuikerspiegels te bereiken en tegelijkertijd te lage en te hoge bloedsuikerwaarden te vermijden. De voorgestelde insuline-dosage wordt door de verpleegkundige, op basis van zijn/haar expertise, geëvalueerd. De verpleegkundige behoudt te alle tijden het recht om het advies indien nodig te corrigeren.

Te lage bloedsuikerspiegels (hypoglycemie) vormen een belangrijk risico dat typisch geassocieerd is met het normaliseren van de bloedsuikerspiegels. De overbehandeling van hypoglycemie kan eveneens aanleiding geven tot sterke schommelingen van de bloedsuikerspiegels (hoge variabiliteit). Zowel te lage, te hoge (hyperglycemie) alsook sterk schommelende bloedsuikerspiegels worden geassocieerd met een minder gunstig genezingsproces van kritieke ziekte. Bij de klassieke manier van werken, tracht de verpleegkundige op basis van zijn/haar expertise hypoglycemie, hyperglycemie en hoge glucosevariabiliteit te vermijden. Dezelfde doelstelling wordt gehanteerd bij het LOGIC-Insulin computerprogramma. Een aanpassing van de hoeveelheid toe te dienen insuline of eventueel een extra toe te dienen glucosehoeveelheid wordt geadviseerd. Een alarm zal tevens waarschuwen wanneer de periode voor een nieuwe bepaling van de bloedsuikerspiegel wordt overschreden.

Mogelijk voordeel van deze studie is dat dosering van insuline efficiënter en veiliger verloopt. De behandeling met het LOGIC-Insulin computerprogramma zal ten slotte gestaakt worden in geval van herhaaldelijke hypoglycemie en oncorrigeerbare hyperglycemie.

De studie gebeurt enkel tijdens het verblijf op de intensieve zorgenafdeling en legt u geen beperking op. Bij ontslag uit de intensieve zorgenafdeling en bij het ziekenhuisontslag zullen we eveneens een vragenlijst i.v.m. de levenskwaliteit bezorgen. Het invullen hiervan vraagt slechts enkele minuten.

De studie zal 1550 patiënten rekruteren en heeft gelijktijdig plaats in het UZ Leuven, het Jessa Ziekenhuis in Hasselt en het AMC in Amsterdam, Nederland. Uw bloedsuikerspiegel werd genormaliseerd hetzij op de klassieke manier, hetzij op advies van het computerprogramma. Deze toewijzing gebeurde door middel van toevalstrekking.

Deze studie brengt geen enkele extra kost met zich mee. U kan steeds vragen om de studie stop te zetten indien u dat wenst. Gedurende het hele verblijf op de Intensieve Zorgen - afdeling krijgt u uiteraard alle nodige zorgen en behandelingen volgens de huidige stand van de geneeskunde. De medische gegevens zullen gecodeerd worden. Na de studie zullen deze worden verwerkt in een wetenschappelijke publicatie. Strikte bewaring van de anonimiteit wordt hierbij gegarandeerd.

Conform de Belgische wet inzake experimenten op de menselijke persoon van 7 mei 2004 is de opdrachtgever van het onderzoek zelfs foutloos, aansprakelijk voor alle schade die de deelnemer of, in geval van overlijden, zijn rechthebbenden oplopen en die rechtstreeks dan wel onrechtstreeks verband houdt met het experiment. De opdrachtgever heeft een verzekering afgesloten die deze aansprakelijkheid dekt. Indien u schade zou oplopen ten gevolge van uw deelname aan deze studie zal die schade bijgevolg worden vergoed conform de Belgische wet inzake experimenten op de menselijke persoon van 7 mei 2004. De gegevens voor de verzekering zijn: Vanbreda Risk & Benefits NV, Plantin en Moretuslei 297, 2140 Antwerpen (polisnummer 299.053.700)

Deze studie werd goedgekeurd door de Commissie voor Medische Ethiek van de UZ Leuven, die als centrale commissie fungeert, na raadpleging van de ethische commissies van de andere deelnemende ziekenhuizen.

Bijzondere bepalingen voor de bescherming van personen van wie de toestemming niet kan worden verkregen wegens hoogdringendheid.
De wet van 7 mei 2004 inzake experimenten op de menselijke persoon legt vast aan welke voorwaarden moeten voldaan zijn indien de toestemming van de patiënt niet kon verkregen worden wegens hoogdringendheid. Het gaat om een levensbedreigende toestand die tot ernstige en blijvende letsels kan leiden, of de dood. Een gunstig advies werd gegeven door de Commissie Medische Ethiek van UZ/KU Leuven. Deze Commissie sprak zich uitdrukkelijk uit over de uitzondering op de regel van de geïnformeerde toestemming voorafgaand aan het experiment. De wet bepaalt ook dat u/de patiënt toestemming dient te geven van zodra u/de patiënt daartoe in staat bent/is, en dat u/de patiënt toestemming geeft voor het verder verzamelen van studiegegevens. Een vertegenwoordiger dient toestemming te geven van zodra het mogelijk is contact met deze persoon op te nemen.

Deze studie wordt uitgevoerd volgens de richtlijnen voor de goede klinische praktijk (ICH/GCP) en volgens de meest recente versie van de verklaring van Helsinki opgesteld ter bescherming van mensen deelnemend aan klinische studies. In geen geval dient u de goedkeuring door de Commissie voor Medische Ethiek te beschouwen als een aansporing tot deelname aan deze studie.

Indien u bijkomende inlichtingen wenst, kan u met uw vragen steeds terecht bij een van de onderzoekers.

Wij danken u alvast voor uw bereidwillige medewerking.

Met de meeste hoogachting,

Prof. Dr. Dieter Mesotten (coördinator)
Adjunct-kliniekhoofd

Prof. Dr. Greet Van den Berghe
Diensthoofd

Dienst intensieve geneeskunde
UZ Leuven
Herestraat 49
3000 Leuven
Tel: 016-344021

Dr. Jasperina Dubois
Lokale coördinator LOGIC-2

Dr. Aimé Van Assche
Diensthoofd

Dienst intensieve geneeskunde
Jessa Ziekenhuis (Campus Virga Jessa)
Stadsomvaart 11
3500 Hasselt
Tel: 011-308971

Toestemmingsverklaring

Ik heb de informatiebrief voor de patiënten gelezen. Ik kon aanvullende vragen stellen. Mijn vragen zijn genoeg beantwoord. Ik had genoeg tijd om te beslissen of ik meedoe.

Ik weet dat meedoen helemaal vrijwillig is. Ik weet dat ik op ieder moment kan beslissen om toch niet mee te doen. Daarvoor hoef ik geen reden te geven.

Ik geef toestemming om mijn verwijzende specialist in te lichten over deelname aan het onderzoek.

Ik geef toestemming om mijn gegevens te gebruiken, voor de doelen die in de informatiebrief staan.

Ik geef toestemming om mijn gegevens nog maximaal 20 jaar na afloop van dit onderzoek te bewaren.

Ik geef toestemming voor deelname aan het onderzoek.

Naam patiënt: ……………………………………………………………………………………

Handtekening:…………………………………………………………………………………….

Datum: …………………………………………………………………………………………….

Ik ondergetekende, bevestig dat ik mondeling de nodige informatie heb gegeven over deze klinische studie, dat ik een kopie heb gegeven van de informatie- en toestemmingsfolder die door de verschillende partijen werd getekend, dat ik bereid ben om zo nodig alle aanvullende vragen te beantwoorden en dat ik geen druk op de patiënt heb uitgeoefend om aan de studie deel te nemen. Ik verklaar dat ik werk volgens de ethische principes die worden beschreven in de Verklaring van Helsinki en de Belgische wet van 7/5/2004 over proeven op mensen.

Naam Arts:…………………………………………………………………………………..……

Handtekening:……………………………………………………………………………….…….

Datum:……………………………………………………………………………………….…..…

Appendix 9: Insurance document

vanbreda
RISK & BENEFITS

Attest aansprakelijkheidsverzekering

Ondergetekende bevestigt hiermee dat een contract burgerlijke aansprakelijkheid op naam van **Katholieke Universiteit Leuven** werd onderschreven via de bemiddeling van Vanbreda Risk & Benefits NV, Plantin en Moretuslei 297, 2140 Antwerpen en op basis van de volgende gegevens:

1. **Verzekeraar(s)**
   Amlin Corporate Insurance

2. **Contractnummer**
   299.053.700

3. **Geldigheidsperiode**
   01/01/2014 – 31/12/2014

4. **Voorwerp van de dekking**
   Clinical trials waarvoor K.U.Leuven/U.Z.Leuven sponsor is en die in overeenstemming zijn met de Wet Experimenten op de menselijke persoon dd. 7/5/2004.

5. **Verzekerde waarborgen**
   per trial: 2.500.000,00 EUR

Antwerpen, 3 oktober 2013

in opdracht van

Jan Van Hecke
Deputy Director

F. Peniquet
Deputy Director

NB: Deze inlichtingen hebben een algemeen karakter. Voor een volledig beeld van de bestaande dekkingen is het noodzakelijk de polis grondig door te nemen.

sca

2.90.000 / 3526564 (0811)

Appendix 10: Notification fee payment

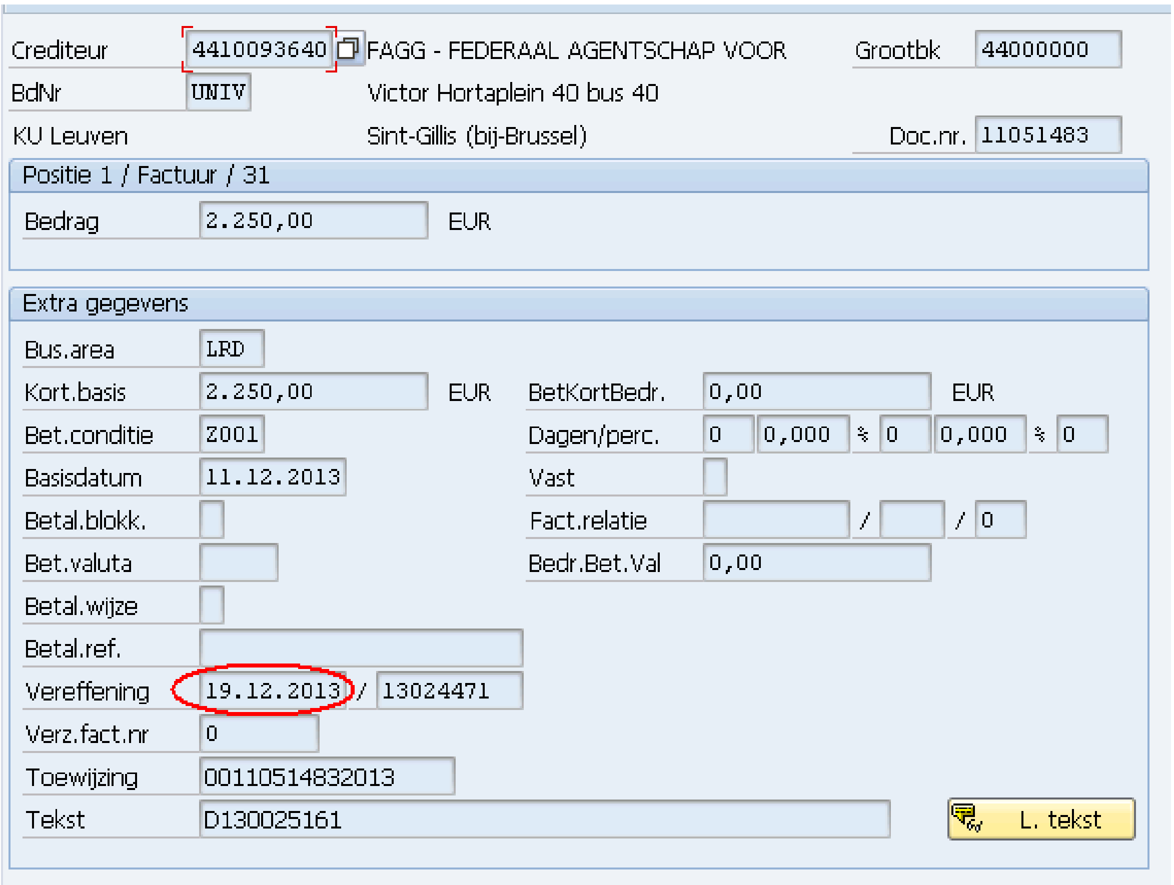

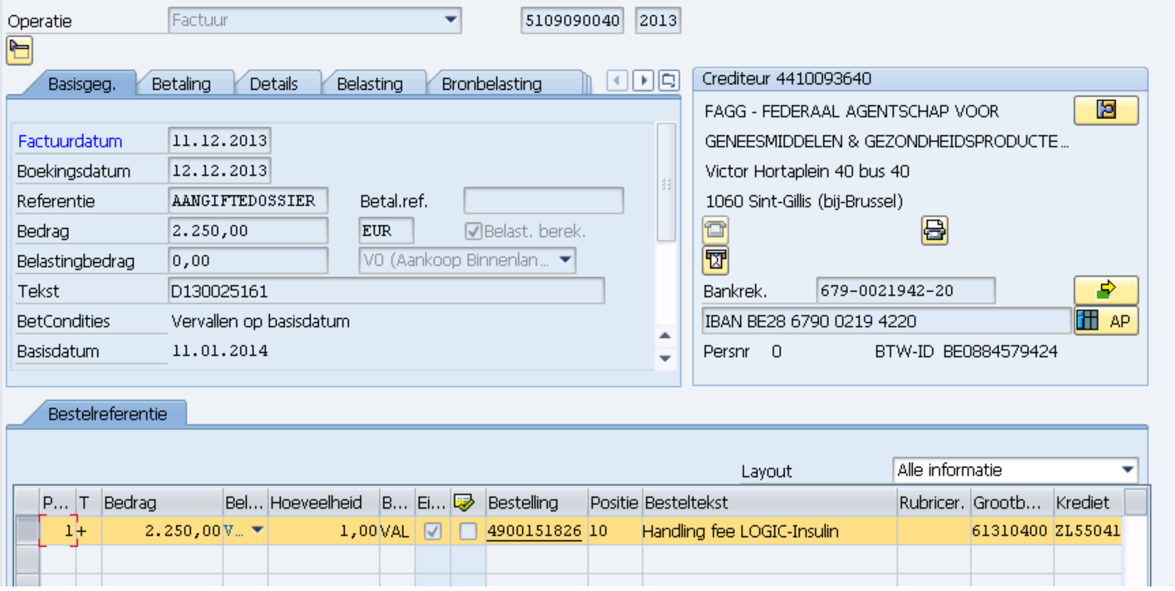

References

1. Bagshaw SM, Egi M, George C, Bellomo R. Early blood glucose control and mortality in critically ill patients in Australia. *Crit Care Med.* Dec 26 2009.

2. Kosiborod M, Rathore SS, Inzucchi SE, et al. Admission glucose and mortality in elderly patients hospitalized with acute myocardial infarction: implications for patients with and without recognized diabetes. *Circulation.* Jun 14 2005;111(23):3078-3086.

3. Sinnaeve PR, Steg PG, Fox KA, et al. Association of elevated fasting glucose with increased short-term and 6-month mortality in ST-segment elevation and non-ST-segment elevation acute coronary syndromes: the Global Registry of Acute Coronary Events. *Arch Intern Med.* Feb 23 2009;169(4):402-409.

4. Van den Berghe G, Wouters P, Weekers F, et al. Intensive insulin therapy in the critically ill patients. *N Engl J Med.* Nov 8 2001;345(19):1359-1367.

5. Van den Berghe G, Wilmer A, Hermans G, et al. Intensive insulin therapy in the medical ICU. *N Engl J Med.* Feb 2 2006;354(5):449-461.

6. Vlasselaers D, Milants I, Desmet L, et al. Intensive insulin therapy for patients in paediatric intensive care: a prospective, randomised controlled study. *Lancet.* Feb 14 2009;373(9663):547-556.

7. Krinsley JS. Effect of an intensive glucose management protocol on the mortality of critically ill adult patients. *Mayo Clin Proc.* Aug 2004;79(8):992-1000.

8. Furnary AP, Cheek DB, Holmes SC, Howell WL, Kelly SP. Achieving tight glycemic control in the operating room: lessons learned from 12 years in the trenches of a paradigm shift in anesthetic care. *Semin Thorac Cardiovasc Surg.* Winter 2006;18(4):339-345.

9. Reed CC, Stewart RM, Sherman M, et al. Intensive insulin protocol improves glucose control and is associated with a reduction in intensive care unit mortality. *J Am Coll Surg.* May 2007;204(5):1048-1054; discussion 1054-1045.

10. Eslami S, Abu-Hanna A, de Keizer NF, et al. Implementing glucose control in intensive care: a multicenter trial using statistical process control. *Intensive Care Med.* Sep 2010;36(9):1556-1565.

11. Schultz MJ, Harmsen RE, Korevaar JC, et al. Adoption and implementation of the original strict glycemic control guideline is feasible and safe in adult critically ill patients. *Minerva Anestesiol.* Sep 2012;78(9):982-995.

12. Brunkhorst FM, Engel C, Bloos F, et al. Intensive insulin therapy and pentastarch resuscitation in severe sepsis. *N Engl J Med.* Jan 10 2008;358(2):125-139.

13. Finfer S, Chittock DR, Su SY, et al. Intensive versus conventional glucose control in critically ill patients. *N Engl J Med.* Mar 26 2009;360(13):1283-1297.

14. Preiser JC, Devos P, Ruiz-Santana S, et al. A prospective randomised multi-centre controlled trial on tight glucose control by intensive insulin therapy in adult intensive care units: the Glucontrol study. *Intensive Care Med.* Oct 2009;35(10):1738-1748.

15. Van den Berghe G, Bouillon R, Mesotten D. Glucose control in critically ill patients. *N Engl J Med.* Jul 2 2009;361(1):89; author reply 91-82.

16. Van den Berghe G, Schetz M, Vlasselaers D, et al. Clinical review: Intensive insulin therapy in critically ill patients: NICE-SUGAR or Leuven blood glucose target? *J Clin Endocrinol Metab.* Sep 2009;94(9):3163-3170.

17. Campbell M, Fitzpatrick R, Haines A, et al. Framework for design and evaluation of complex interventions to improve health. *BMJ.* Sep 16 2000;321(7262):694-696.

18. Scott MG, Bruns DE, Boyd JC, Sacks DB. Tight glucose control in the intensive care unit: are glucose meters up to the task? *Clin Chem.* Jan 2009;55(1):18-20.

19. Van Herpe T, De Moor B, Van den Berghe G. Towards closed-loop glycaemic control. *Best Pract Res Clin Anaesthesiol.* Mar 2009;23(1):69-80.

20. Fahy BG, Sheehy AM, Coursin DB. Glucose control in the intensive care unit. *Crit Care Med.* May 2009;37(5):1769-1776.

21. Vogelzang M, van der Horst IC, Nijsten MW. Hyperglycaemic index as a tool to assess glucose control: a retrospective study. *Crit Care.* Jun 2004;8(3):R122-127.

22. Van Herpe T, De Brabanter J, Beullens M, De Moor B, Van den Berghe G. Glycemic penalty index for adequately assessing and comparing different blood glucose control algorithms. *Crit Care.* 2008;12(1):R24.

23. Finfer S, Wernerman J, Preiser JC, et al. Clinical review: Consensus recommendations on measurement of blood glucose and reporting glycemic control in critically ill adults. *Critical Care.* 2013;17

24. Moghissi ES, Korytkowski MT, DiNardo M, et al. American Association of Clinical Endocrinologists and American Diabetes Association consensus statement on inpatient glycemic control. *Endocr Pract.* Jul-Aug 2009;15(4):353-369.

25. Van den Berghe G, Wilmer A, Milants I, et al. Intensive insulin therapy in mixed medical/surgical intensive care units: benefit versus harm. *Diabetes.* Nov 2006;55(11):3151-3159.

26. Mesotten D, Gielen M, Sterken C, et al. Neurocognitive development of children 4 years after critical illness and treatment with tight glucose control: a randomized controlled trial. *JAMA.* Oct 24 2012;308(16):1641-1650.

27. Finfer S, Liu B, Chittock DR, et al. Hypoglycemia and risk of death in critically ill patients. *N Engl J Med.* Sep 20 2012;367(12):1108-1118.

28. Krinsley JS, Schultz MJ, Spronk PE, et al. Mild hypoglycemia is independently associated with increased mortality in the critically ill. *Crit Care.* 2011;15(4):R173.

29. Dortch MJ, Mowery NT, Ozdas A, et al. A computerized insulin infusion titration protocol improves glucose control with less hypoglycemia compared to a manual titration protocol in a trauma intensive care unit. *JPEN J Parenter Enteral Nutr.* Jan-Feb 2008;32(1):18-27.

30. Cordingley JJ, Vlasselaers D, Dormand NC, et al. Intensive insulin therapy: enhanced Model Predictive Control algorithm versus standard care. *Intensive Care Med.* Jan 2009;35(1):123-128.

31. Mesotten D, Van den Berghe G. Glycemic targets and approaches to management of the patient with critical illness. *Curr Diab Rep.* Feb 2012;12(1):101-107.

32. Van Herpe T, Mesotten D, Wouters PJ, et al. LOGIC-insulin algorithm-guided versus nurse-directed blood glucose control during critical illness: the LOGIC-1 single-center, randomized, controlled clinical trial. *Diabetes Care.* Feb 2013;36(2):188-194.

33. Deedwania P, Kosiborod M, Barrett E, et al. Hyperglycemia and acute coronary syndrome: a scientific statement from the American Heart Association Diabetes Committee of the Council on Nutrition, Physical Activity, and Metabolism. *Circulation.* Mar 25 2008;117(12):1610-1619.

34. Cryer PE. The barrier of hypoglycemia in diabetes. *Diabetes.* Dec 2008;57(12):3169-3176.

35. Faustino EV, Bogue CW. Relationship between hypoglycemia and mortality in critically ill children. *Pediatr Crit Care Med.* Nov 2010;11(6):690-698.

36. Meyfroidt G, Keenan DM, Wang X, Wouters PJ, Veldhuis JD, Van den Berghe G. Dynamic characteristics of blood glucose time series during the course of critical illness: effects of intensive insulin therapy and relative association with mortality. *Crit Care Med.* Apr 2010;38(4):1021-1029.

37. Krinsley JS. Glycemic variability: a strong independent predictor of mortality in critically ill patients. *Crit Care Med.* Nov 2008;36(11):3008-3013.

38. Vanhorebeek I, Gielen M, Boussemaere M, et al. Glucose Dysregulation and Neurological Injury Biomarkers in Critically Ill Children. *J Clin Endocrinol Metab.* Jul 28 2010;95(10):4669-4679.

39. Van den Berghe G, Mesotten D, Vanhorebeek I. Intensive insulin therapy in the intensive care unit. *CMAJ.* Apr 14 2009;180(8):799-800.

40. Kosiborod M, Inzucchi SE, Goyal A, et al. Relationship between spontaneous and iatrogenic hypoglycemia and mortality in patients hospitalized with acute myocardial infarction. *JAMA.* Apr 15 2009;301(15):1556-1564.

41. Vanderheyden S, Casaer MP, Kesteloot K, et al. Early versus late parenteral nutrition in ICU patients: cost analysis of the EPaNIC trial. *Crit Care.* May 25 2012;16(3):R96.
